# Identifying gaps in the continuum of care for hypertension and diabetes in two Indian communities

**DOI:** 10.1186/s12913-017-2796-9

**Published:** 2017-12-27

**Authors:** Rose Gabert, Marie Ng, Ruchi Sogarwal, Miranda Bryant, R. V. Deepu, Claire R. McNellan, Sunil Mehra, Bryan Phillips, Marissa Reitsma, Blake Thomson, Shelley Wilson, Alexandra Wollum, Emmanuela Gakidou, Herbert C. Duber

**Affiliations:** 1 Institute for Health Metrics and Evaluation, University of Washington, 2301 5th Ave, Suite 600, Seattle, WA 98121 USA; 2 Department of Emergency Medicine, University of Washington, Seattle, WA USA; 3 MAMTA Health Institute for Mother and Child, New Delhi, India

**Keywords:** Effective coverage, Diabetes, Hypertension, Cardiovascular disease, India, Continuum of care

## Abstract

**Background:**

Non-communicable diseases (NCDs) represent the largest, and fastest growing, burden of disease in India. This study aimed to quantify levels of diagnosis, treatment, and control among hypertensive and diabetic patients, and to describe demand- and supply-side barriers to hypertension and diabetes diagnosis and care in two Indian districts, Shimla and Udaipur.

**Methods:**

We conducted household and health facility surveys, as well as qualitative focus group discussions and interviews. The household survey randomly sampled individuals aged 15 and above in rural and urban areas in both districts. The survey included questions on NCD knowledge, history, and risk factors. Blood pressure, weight, height, and blood glucose measurements were obtained. The health facility survey was administered in 48 health care facilities, focusing on NCD diagnosis and treatment capacity, including staffing, equipment, and pharmaceuticals. Qualitative data was collected through semi-structured key informant interviews with health professionals and public health officials, as well as focus groups with patients and community members.

**Results:**

Among 7181 individuals, 32% either reported a history of hypertension or were found to have a systolic blood pressure ≥ 140 mmHg and/or diastolic ≥90 mmHg. Only 26% of those found to have elevated blood pressure reported a prior diagnosis, and just 42% of individuals with a prior diagnosis of hypertension were found to be normotensive. A history of diabetes or an elevated blood sugar (Random blood glucose (RBG) ≥200 mg/dl or fasting blood glucose (FBG) ≥126 mg/dl) was noted in 7% of the population. Among those with an elevated RBG/FBG, 59% had previously received a diagnosis of diabetes. Only 60% of diabetics on treatment were measured with a RBG <200 mg/dl. Lower-level health facilities were noted to have limited capacity to measure blood glucose as well as significant gaps in the availability of first-line pharmaceuticals for both hypertension and diabetes.

**Conclusions:**

We found high rates of uncontrolled diabetes and undiagnosed and uncontrolled hypertension. Lower level health facilities were constrained by capacity to test, monitor and treat diabetes and hypertension. Interventions aimed at improving patient outcomes will need to focus on the expanding access to quality care in order to accommodate the growing demand for NCD services.

**Electronic supplementary material:**

The online version of this article (10.1186/s12913-017-2796-9) contains supplementary material, which is available to authorized users.

## Background

Recent decades have shown unprecedented focus, and significant progress, in tackling infectious diseases. At the same time, increased longevity, physical inactivity, dietary changes, and other social, cultural, and environmental factors have had the combined effect of dramatically increasing the prevalence of cardiometabolic disorders such as hypertension and diabetes mellitus. [1, 2] In many countries, including most low-and middle-income countries (LMICs), this combination of factors has led to an epidemiologic transition where non-communicable diseases (NCDs) have now surpassed infectious diseases as the largest contributor to disease burden.

The World Health Organization (WHO) has projected that cardiovascular disease (CVD) alone will account for nearly a quarter of global deaths by 2030. [3] Similarly, the International Diabetes Federation estimated that the number of individuals living with diabetes would increase from approximately 65 million in 2013 to 100 million by 2035. [4] Despite the increasing burden of NCDs, both research and the allocation of resources to combat NCDs remain limited. [5, 6]

India, the second most populous country in the world, is one example of this rapid change in NCD burden. In 2013, NCDs were estimated to account for 52% of India’s total disease burden, a stark contrast to 1990, when they accounted for just 32%. [2] Following the WHO *Global action plan for the prevention and control of NCDs 2013–2020*, India became the first country to set national targets and specific indicators to measure progress towards preventing and controlling NCDs. [7, 8] Meeting India’s stated target of a 25% reduction in premature mortality from NCDs by 2025 will require a multi-faceted approach designed to improve clinical care, strengthen community-level knowledge of NCDs, and ultimately improve patient outcomes.

Building a successful NCD program necessitates an understanding of current barriers throughout the entire spectrum of NCD care—from knowledge of risk factors through disease control. Prior studies have shown that a large proportion of those living with diabetes or hypertension are either unaware of their condition, do not receive treatment for their condition, or live with uncontrolled disease despite receiving treatment. [9–14] A recent meta-analysis and systematic review suggested a large magnitude of undiagnosed, untreated or uncontrolled hypertensive individuals in communities throughout India. [15] To date, no study has comprehensively evaluated the continuum of care for hypertension and diabetes in India, including key barriers to care.

To address this knowledge gap and identify solutions to the growing burden of NCDs, Medtronic Foundation launched the HealthRise project to improve care for NCDs, with a specific focus on hypertension and diabetes. This 5-year initiative seeks to identify barriers, and to implement and evaluate innovative community-based strategies empowering front line health workers to test, treat, and help patients successfully manage CVD and diabetes before complications arise. In addition to direct intervention, HealthRise is engaging public and private stakeholders to advocate for increased action on NCDs, and rigorously evaluating interventions that have the potential to contribute to a growing body of evidence around NCD management in high-burden settings. The project focuses on select underserved communities with high levels of undiagnosed and poorly managed hypertension and diabetes in four countries: India, South Africa, Brazil, and the United States.

Although the primary goal of the project is to develop an evidence base for community-based interventions, the first step in that process is to identify baseline characteristics and barriers to care within the select communities of interest. This information will be utilized as part of the intervention development process to ensure appropriate focus on identified targets within the continuum of care. In this paper, we present the results of a hypertension and diabetes needs assessment conducted in two Indian districts.

## Methods

### Theoretical framework

**Fig. 1:**
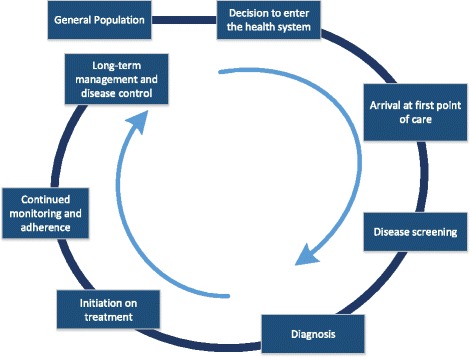
The Continuum of Care Framework

We used an analytical framework that would broadly capture information on both supply- and demand-side barriers to diagnosis, treatment, and management of hypertension and diabetes. The continuum of care is a comprehensive approach to identify potential barriers at each stage of seeking, accessing, and receiving consistent, appropriate and quality care. We used a continuum of care framework to understand the gaps between individuals and the health care system and prioritize the most critical challenges in service delivery and access. We also sought to quantify the proportion of patients along each step in the continuum of care, focusing largely on individuals with undiagnosed disease, diagnosed but untreated disease, and treated but inadequately controlled disease. (Fig. 1).

### Study setting and design

As a community-based initiative, small geographies (Shimla, in Himachal Pradesh, and Udaipur, in Rajasthan) were selected to study local gaps along the continuum of care. These sites were selected a priori in collaboration with government stakeholders based on the presence of a strong tertiary health care system, previous successful implementation of other community-based health programs at the district-level, and active engagement of state- and district-level leadership. [16] In addition, these two sites represent a diverse range of challenges (including poverty, low education, and limited access to care) faced by the local community and its health system. In 2011, the population of Shimla was 814,010 individuals, 75% of whom lived in rural areas; in Udaipur, the 2011 population was 3,068,420, with 80% living in rural areas. Literacy rates were higher in Shimla (84%) than in Udaipur (62%). [17, 18]

This mixed-methods needs assessment utilized a household survey (Additional file 1), a health facility survey (Additional file 2), and qualitative focus group discussions (Additional file 3) and interviews (Additional file 4). Each is described in further detail below.

#### Household survey

The household survey (Additional file 1) was conducted by MAMTA Health Institute for Mother and Child in 2013 through a partnership with Medtronic Foundation. The survey instrument collected information on sociodemographic background, risk factors, medical history, and knowledge, attitudes, and practices related to NCDs. Anthropometric data, including measurements of blood pressure, blood glucose (random or fasting), height, and weight were also collected.

The household survey aimed to sample 4400 individuals aged 15 and above in each district with at least 220 individuals in each 10-year age group for both sexes. For both districts, half the sample was drawn from areas designated as “urban wards” and half from “rural villages” according to the 2011 India census definitions. In urban wards a three stage sampling method was used. Systematic random sampling was used to select 22 wards as the primary sampling units (PSUs). Each selected ward was then divided into segments having approximately 250 households and one segment was selected at random from these. Finally, within each selected segment, 80 households were selected at random. In rural villages, a two-stage method was used. First, 22 rural villages were chosen at random as the PSUs, followed by random selection of approximately 80 households within each of the selected villages. For both urban and rural households one individual in the age range 15–54 was chosen per household along with all of the individuals in the age group 55 and above. The final sample yielded 7181 individuals across the two districts. [16]

#### Health facility survey

The health facility survey (Additional file 2) was designed by the Institute for Health Metrics and Evaluation at the University of Washington and adapted to the local context by IGMC-Shimla and GfK Mode. GfK Mode translated the instrument into Hindi. The instrument collected information regarding facility-based resources, finances, and volume of services and procedures provided. Additionally, the instrument captured information about NCD-specific services, pharmaceuticals, stock-outs, and personnel capacity using a combination of the WHO Package of essential NCD interventions for primary health care and the Government of India Ministry of Health and Family Welfare Indian Public Health Standards (IPHS) 2012. [19, 20]

Health facility sampling was designed to include facilities from both the public and private sector and each of the four tiers of public service delivery. The public health system is divided into four sequential levels of care-providing platforms: tertiary care centers such as District or Zonal hospitals and Medical Colleges that provide comprehensive care including surgical and in-patient care; secondary care centers called Community Health Centers (CHCs) that provide standard laboratory investigations, diagnostics, and disease management along with some in-patient care; and finally Primary Health Centers (PHCs) and Sub-Centers (SCs) providing care closest to patients in rural communities and referring up the chain of facilities for more complicated cases. Public health facilities were selected using a stratified and staged random sample by first randomly sampling among CHCs. A random sample of PHCs reporting to selected CHCs were then chosen, followed by random selection of SCs from within the pool of SCs reporting to the selected PHCs. Private facilities were sampled at random with the objective of selecting approximately equal numbers of clinics and hospitals. The final sample included a total of 48 facilities (Table 1). Sampling frames were obtained from the Chief Medical Officers of each district, including a list of all public facilities in the district sorted by facility tier and a list of all private facilities registered by the Chief Medical Officer.

**Table 1:** Facility sample by district and platform

Platform	Shimla	Udaipur
Tertiary Hospitals (Including District Hospitals, Zonal Hospitals)	2	2
Community Health Center (CHC)	2	2
Primary Health Center (PHC)	4	5
Sub-Center (SC)	8	8
Private Hospital	4	5
Private Clinic	3	3
Total	23	25

Following a one-week training and pilot of the survey instrument, health facility assessments were conducted in Udaipur and Shimla between December 2014 and January 2015.

#### Qualitative interviews and focus groups

Qualitative data were collected through focus group discussions with community members and patients with NCDs (Additional file 3), and semi-structured individual interviews (Additional file 4) with health officials and healthcare providers, including doctors, nurses, and community health workers. In total, 25 individual interviews and 17 focus group discussions were conducted. Twenty interviews and focus group discussions were held in Shimla and 22 were held in Udaipur in order to capture differences between the two districts (Table 2). Qualitative data was collected by trained research assistants in both Shimla and Udaipur between December 2014 and February 2015.

**Table 2:** Interview and focus group participants

Interviewees	Shimla	Udaipur
ASHAs	3 interviews	2 interviews / 1 FGD
Healthcare Providers: PHC doctors, Public GP, Private GP, cardiology specialist, diabetes specialist	5 interviews	5 interviews
Health Officials	2 interviews	3 interviews
NGO Officials	1 interview	1 interview
Nurses	1 interview	2 interviews
General Community Members	4 FGD	4 FGD
NCD Patients	4 FGD	4 FGD
Total	12 interviews / 8 FGD	13 interviews / 9 FGD

### Data management and analysis

Quantitative data (household and health facility survey) was uploaded via a secure encrypted data transfer system. Qualitative data was transcribed and translated into English and resulting files were shared via secure encrypted data transfer. All data was stored on password-protected servers.

Quantitative data is presented descriptively. Prevalence of risk factors and conditions and the proportion of individuals with disease in each category on the continuum of care were directly calculated as percentages using household survey data. Facility resources were benchmarked against IPHS tier-specific guidelines to determine where personnel or supplies did not meet national standards and the results are presented descriptively as percentages of facilities meeting appropriate standards. All quantitative data were analyzed using Stata version 13.1 (StataCorp. 2013. Stata Statistical Software: Release 13. College Station, TX).

The framework approach was used to analyze qualitative data. [21] Initially, a representative sample of interviews and focus group discussions were reviewed. During this process open coding was used to classify participant responses. After reading and coding the initial group of interviews, a working thematic framework was developed based on emerging themes. The remaining interviews were coded based on the working thematic framework. Data were assessed collectively in order to identify convergent themes, separately by district to ascertain differences between Udaipur and Shimla, and separately by respondent category in order to identify additional divergent themes.

## Results

Sociodemographic data summarizing the household study sample for both districts is presented in Table 3. Survey respondents in both districts were slightly more likely to be female. Those in Shimla tended to have higher household incomes and higher levels of educational attainment. While the overwhelming majority of Shimla respondents were Hindu, 13% of respondents in Udaipur identified as Muslim.

**Table 3:** Household survey respondent characteristics

	Shimla (*n* = 3398)	Udaipur (*n* = 3783)
Female	1792 (53%)	2202 (58%)
Age		
15–30	1125 (33%)	1180 (31%)
31–45	1148 (34%)	1133 (30%)
46–60	768 (23%)	857 (23%)
60+	357 (11%)	613 (16%)
Household Income		
< 5000 rupees	937 (28%)	1805 (48%)
5001–10,000 rupees	854 (25%)	932 (25%)
10,001–15,000 rupees	575 (17%)	362 (10%)
> 15,000 rupees	1032 (30%)	684 (18%)
Education		
No formal education	535 (16%)	1168 (31%)
1st-8th Class	544 (16%)	1065 (28%)
9th–12th Class	1463 (43%)	927 (25%)
University or above	856 (25%)	623 (17%)
Religion		
Hindu	3276 (96%)	3264 (86%)
Muslim	37 (1%)	482 (13%)
Other	85 (3%)	37 (1%)
Caste		
Scheduled caste	649 (19%)	445 (12%)
Scheduled tribe	45 (1%)	725 (19%)
Other backward class	45 (1%)	1035 (27%)
Other	2659 (78%)	1578 (42%)

### Disease and risk factor prevalence

Table 4 outlines the prevalence of diabetes and hypertension, as well as two key modifiable CVD risk factors, weight and tobacco use. Hypertension was highly prevalent in both Shimla (33%) and Udaipur (31%), with a slight male predominance. Diabetes was much less common; 6% of the population in Shimla, and 9% in Udaipur, either reported a history of diabetes or were found to have elevated blood glucose suggestive of diabetes. We found that 38% of the population in Shimla, and 30% of the population in Udaipur, was overweight or obese. Women in both districts were much more likely to be overweight and obese than men. While less than 5% of all women ever reported using tobacco, nearly 40% of men had used tobacco, with slightly higher rates in Udaipur than Shimla.

**Table 4:** Prevalence of cardiometabolic risk factors, hypertension and diabetes

		Shimla			Udaipur		
		Female	Male	Total	Female	Male	Total
Disease Prevalence							
	Hypertension	554 (31%)	572 (36%)	1126 (33%)	686 (31%)	502 (32%)	1188 (31%)
	Diabetes	95 (5%)	98 (6%)	193 (6%)	156 (7%)	142 (9%)	298 (8%)
Tobacco							
	Current Use	25 (1%)	450 (28%)	475 (14%)	115 (5%)	591 (37%)	706 (19%)
	Ever Used	31 (2%)	510 (32%)	541 (16%)	140 (6%)	681 (43%)	821 (22%)
Weight							
	Obese	256 (15%)	71 (5%)	327 (10%)	253 (12%)	77 (5%)	330 (9%)
	Overweight	765 (45%)	463 (31%)	1228 (38%)	701 (34%)	371 (25%)	1072 (30%)

### Gaps in the continuum of care

**Fig. 2:**
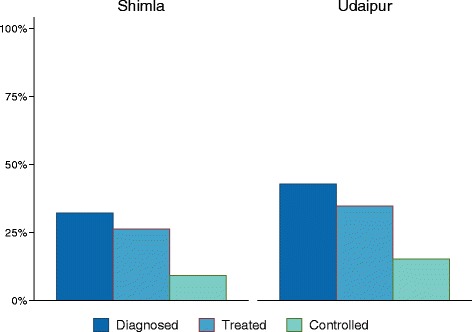
Hypertension Continuum of Care. Percentage of patients who have received a diagnosis of hypertension, initiated therapy, and had normal blood pressure measurements among all individuals with a history of hypertension or elevated blood pressure at time of the survey

Figure 2 outlines the continuum of care for hypertension. Overall, we found that 23% of individuals in Shimla, and 18% in Udaipur, were found to have elevated blood pressures (systolic >140 mmHg or diastolic >90 mmHg) and no prior diagnosis of hypertension. This represents well over half of all patients found to have elevated blood pressures or a reported history of hypertension at the time of the survey. Similar to the disease prevalence findings, men were more likely than women to have elevated blood pressure without a diagnosis of hypertension (30% vs 21% in Shimla and 24% vs. 19% in Udaipur).

A history of hypertension was reported by 11% of individuals in Shimla, and 13% in Udaipur. Among those who had received a prior diagnosis of hypertension, 82% in Shimla and 81% in Udaipur, reported being on pharmacologic treatment. Normal blood pressures were found in 36% and 46% of patients with a prior diagnosis of hypertension in Shimla and Udaipur. Among those with a previous diagnosis, men were less likely than women to have controlled hypertension in both Shimla (26% vs. 30%) and Udaipur (34% vs. 37%).

**Fig. 3:**
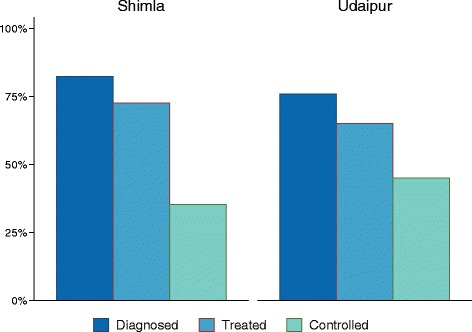
Diabetes Continuum of Care. Percentage of patients who have received a diagnosis of diabetes, initiated therapy, and had normal RBG/FBG among all individuals with a history of diabetes or elevated RBG/FBG at time of the survey

The continuum of care for diabetes patients is presented in Fig. 3. Overall, random blood glucose (RBG) greater ≥200 mg/dl or fasting blood glucose (FBG) ≥126 mg/dl were found in less than 5% of adults surveyed, with slightly higher percentages among men than women. Compared to hypertension, a larger percentage of individuals with an elevated biomarker had already received a prior diagnosis. Among those with elevated RBG or FBG, 41% did not have a prior diagnosis of diabetes. Looking specifically at individuals with a prior diagnosis of diabetes, 88% in Shimla and 86% in Udaipur reported being on pharmacologic treatment. Among previously diagnosed diabetic patients, 49% and 69% were noted to have abnormal RBG or FBG in Shimla and Udaipur, respectively.

### Barriers to diagnosis and disease monitoring

Focus group discussions with patients and community members revealed that individuals were often unlikely to seek care at a health facility until debilitating symptoms presented. Many individuals reported being unaware of the asymptomatic nature of hypertension and the early stages of diabetes. Moreover, patients and community members highlighted the perceived lack of diagnostic equipment and testing capabilities at public facilities as an explanation for not seeking routine check-ups with a primary care provider. Finally, being referred to private laboratories was seen as a further expense in terms of time, travel, and prohibitively high pricing of tests in the private market.

Interviews with providers in the public system concurred, stating that patients were frequently referred to private institutions or higher levels of care for diagnostic testing when supplies were short**.** Quantitatively, we noted important gaps in the availability of diagnostic equipment. Table 5 shows the percentage of facilities found to have key NCD equipment on the day of the survey. Although sphygnomonometers were present at all public facilities, many facilities did not have all requisite equipment per IPHS-mandated guidelines. In fact, none of the public hospitals in Shimla and only one in Udaipur met the IPHS guideline. Among CHCs, half met the BP apparatus guidelines in Shimla, and none in Udaipur. PHCs and SCs generally met IPHS standards, though facilities in Shimla generally had less NCD equipment than those in Udaipur. It should also be noted that glucometers and test strips were not available at many of the lower-level public facilities. However, they are not explicitly required at these levels of care per IPHS guidelines.

**Table 5:** Percentage of facilities with key NCD equipment on day of survey

Platform type	Public Hospital	CHC	PHC	SC	Private Hospital	Private Clinic
Shimla						
Adult Scale	**100**	**50**	**100**	**100**	100	67
BP Apparatus	**100**	**100**	**100**	**100**	100	67
Glucometer	**50**	50	50	0	100	33
Glucometer Test Strips	**50**	50	75	0	100	33
Blood Chemistry Analyzer	**50**	100	–	–	100	0
Udaipur						
Adult Scale	**100**	**100**	**80**	**100**	100	67
BP Apparatus	**100**	**100**	**100**	**100**	100	100
Glucometer	**100**	100	100	0	100	67
Glucometer Test Strips	**100**	100	100	0	80	67
Blood Chemistry Analyzer	**100**	50	–	–	60	67

Bold numbers indicate required equipment as per IPHS guidelines. Blood chemistry analyzers were not asked about at the PHC or SC level

### Barriers to treatment and disease control

A closer look at facility-based pharmaceutical supplies finds important gaps in the availability of required pharmaceuticals (Table 6). First-line medications for diabetes management were available in the majority of public facilities, although sulphonylureas and insulin were lacking in Shimla public hospitals. PHCs, a key point of entry and continued care for patients with NCDs, were generally found to have insufficient supplies of both hypertension and diabetes medications, although the PHCs in Udaipur performed slightly better than the PHCs in Shimla. Interestingly, while private hospitals in Shimla tended to be better stocked than public facilities, the opposite was true in Udaipur.

**Table 6:** Pharmaceutical availability at health facilities on day of survey

	Platform type	Public Hospital	CHC	PHC	SHC	Private Hospital	Private Clinic
Shimla							
Hypertension	Calcium Channel Blockers	**100**	**100**	**50**	0	100	0
	Beta Blocker	**100**	**100**	**25**	0	75	0
	ACE Inhibitors	**50**	**100**	**50**	0	75	0
Diabetes	Biguanides	**100**	**100**	**75**	0	75	0
	Sulphonylureas	**0**	**100**	**50**	0	75	0
	Insulin	**50**	**0**	**0**	0	75	0
High Cholesterol	Statin	**50**	**0**	0	0	75	0
Other	Salicylate (aspirin)	**50**	**50**	**25**	0	75	0
	Diuretic	**100**	**0**	**0**	0	75	0
Udaipur							
Hypertension	Beta Blocker	**100**	**100**	**60**	0	60	0
	Calcium Channel Blockers	**100**	**100**	**60**	0	40	0
	ACE Inhibitors	**100**	**0**	**0**	0	40	0
Diabetes	Biguanides	**100**	**100**	**80**	0	40	0
	Sulphonylureas	**100**	**50**	**60**	0	40	0
	Insulin	**100**	**0**	**20**	0	40	0
High Cholesterol	Statin	**100**	**0**	0	0	40	0
Other	Diuretic	**100**	**100**	**80**	0	40	0
	Salicylate (aspirin)	**50**	**50**	**40**	0	40	0

Bold numbers indicate required pharmaceutical as per IPHS guidelines

Stock-outs of drugs in the public health system were also highlighted as a major barrier to NCD management in focus group discussions with patients and community members as well as interviews with providers in the public health system. As with diagnostic services, many patients and providers reported that after receiving a prescription in a public facility, patients may be forced to go to a private pharmacy or chemist to access the required pharmaceuticals, adding barriers of increased time, travel, and medication cost.

Additionally, many public providers voiced concern with the lack of time they are able to spend with patients to properly educate them about their condition. They often referenced insufficient time to explain medications or provide counselling regarding healthy behaviors and lifestyle modifications. This observation corresponded with patients’ reporting long wait times and insufficient time with providers at public facilities.

## Discussion

Our study of adult populations in Shimla and Udaipur found high rates of key cardiometabolic risk factors, undiagnosed and uncontrolled hypertension, and uncontrolled diabetes. Similar to adult populations in other LMICs, the prevalence of tobacco use and obesity was found to be high in both Shimla and Udaipur. [22–25] Moreover, we observed that while men were far more likely to have used tobacco than women, women were twice as likely to be obese, a skewed distribution of risk factors also observed in many prior studies conducted in LMICs, including India. [23, 26]

In both Shimla and Udaipur, we found hypertension to be common, yet the majority of individuals with elevated blood pressures reported no history of a prior diagnosis. Our hypertension prevalence estimates are slightly higher than previous studies in India, but minor variation is not surprising due to a combination of sociodemographic, educational, geographic and other potential differences in study populations. [15, 27–29] Several studies in India have estimated similarly low levels of disease status awareness; a 2014 meta-analysis estimated that just 25% of rural and 42% of urban Indians with hypertension were aware of their status. [15] These findings reinforce the need to improve health education and access to appropriate diagnostic services for hypertension in order to identify a disease that is often silent until it leads to acute cardiovascular events.

In contrast, individuals with an elevated blood glucose were much more likely to have received a diagnosis, probably due to a higher frequency of manifesting symptoms. Qualitatively, we found that many people waited until they were symptomatic to seek treatment, again suggesting the need for improved health education and outreach services. This finding is consistent with other studies that have demonstrated screening for those without symptoms is essential to improve rates of early diagnosis, and this barrier is well-documented in India and other LMICs. [16, 17] Given the lack of glucometers at SCs, and in Shimla PHCs, this would suggest that facilities require both supplies and training in order to reinforce asymptomatic screening among those at risk. A revision of the 2012 IPHS guidelines making glucometers mandatory at all health facilities would represent a significant step in this direction, perhaps leading to higher rates of diagnosis and decreasing the incidence of patients bypassing lower-level facilities in favor of higher-level facilities with larger stores of pharmaceuticals and diagnostic tests.

As in other studies, once patients received a diagnosis of hypertension or diabetes, they frequently initiated treatment. [11–13, 15, 29] This is an important finding as it suggests that patients in communities with traditionally high rates of alternative medicine were willing to initiate allopathic treatment. At the same time, we note significant challenges to ongoing care. Although tertiary care facilities, and to a lesser extent CHCs, were well-stocked with pharmaceuticals, PHCs were not. This gap necessitates patients obtaining medications at outside pharmacies or seeking care at higher-level facilities. [30–32] A focus on improving the availability of common, IPHS-required medications for the maintenance of chronic conditions at PHCs, and possibly expanding to SCs and beyond, has the potential to medication availability, adherence, and disease control.

Other important, non-pharmaceutical, barriers to ongoing hypertension and diabetes care were also observed. Specifically, we note a theme of significant limitations in the availability and duration of patient-physician consultations. Duration was most commonly limited by high patient volumes, coupled with limited physician and nursing human resources. As a result, health education was often given little attention, or completely overlooked. Furthermore, transportation availability and cost were often mentioned as important barriers to care, even once patients had initiated treatment. For chronic diseases such as hypertension and diabetes, ongoing monitoring is critical. Patients unable to seek care on a regular basis will often fall short of achieving optimal medical therapy, and fail to meet treatment goals. Poor rates of hypertension and diabetes control in both Shimla and Udaipur will require strategies that provide for increased health education opportunities and improved access to care.

The HealthRise project will attempt to address key challenges throughout the entire continuum of care by utilizing task-shifting strategies and increasing community involvement and empowerment. Intervention in Shimla and Udaipur will use CHWs and Accredited Social Health Activists (ASHAs), an important lower-level community-oriented provider within the Indian public health system, as an extension of the formal health care system to improve testing, treatment and control for diabetes and hypertension. Through a decentralized, community-based model, CHWs and ASHAs have already been proven to be effective at improving HIV prevention and maternal and child health outcomes throughout India. [33–35] [36, 37] Previously successful interventions focused on point-of-care diagnostic testing and improving access to HIV pharmaceuticals have the potential to be adapted and fill an important void in NCD care as well. [38]

This study should be viewed within its limitations. First, data was collected as a one-time, cross-sectional measure of risk factors, diabetes, and hypertension. Unfortunately, the response rate for the household survey was unreported, and if high, could lead to non-response bias. Second, time and budget constraints only allowed for blood sugar and blood pressure measurements to occur at the time of household survey administration. This is an important limitation, as the diagnosis of hypertension requires a second blood pressure measurement within two weeks after the initial reading, and a single random measurement of hyperglycemia itself does not uniformly equate to a diagnosis of diabetes. This would suggest that our results are likely an overestimation of disease prevalence. However, in the Indian context, especially in communities with poor access to care, it is likely that additional repeated measures will not always be performed. Finally, qualitative data was collected by convenience sample, and therefore perceived barriers to care may not represent the study population. On the other hand, our findings are consistent with previously published literature, suggesting their validity.

## Conclusions

NCDs represent the largest burden of disease in India, with expectations that it will continue to grow. Among residents in two Indian districts, Shimla and Udaipur, we found high rates of undiagnosed and uncontrolled hypertension, and uncontrolled diabetes. We identified numerous barriers to care, including access to chronic disease screening and care, and testing equipment and pharmaceutical availability at lower level health facilities. Based on these findings, a greater emphasis on building diagnostic capacity and treatment services at PHCs and SCs, and a focus on extending the formal health care system through community health workers to improve health education and access are strategies worth pursuing in an effort to improve outcomes. This baseline assessment will be essential for evaluating the HealthRise interventions, and provide a growing evidence base for NCD care in India.

## Additional files

Additional file 1: Household survey. (PDF 839 kb)

Additional file 2: Health facility survey. (PDF 2323 kb)

Additional file 3: Focus group discussion protocols for patients and community members. (PDF 889 kb)

Additional file 4: Key informant interview protocols for physicians, health system officials/policymakers and nurses/ASHAs. (PDF 943 kb)

## Abbreviations

ASHA: Accredited Social Health Activist
BP: Blood Pressure
CHC: Community health center
CHW: Community health worker
CVD: Cardiovascular disease
DBP: Diastolic blood pressure
HIV/AIDS: Human-immunodeficiency virus/Acquired Immune Deficiency Syndrome
IPHS: Indian Public Health Standards
LMIC: Low- and middle-income countries
NCD: Non-communicable diseases
PHC: Primary health center
PSU: Primary sampling unit
SBP: Systolic blood pressure
SC: Sub-Center
WHO: World Health Organization
