# Supplementary material for: Identifying gaps in the continuum of care for hypertension and diabetes in two Indian communities
Source: BMC Health Serv Res. 2017 Dec 27;17:846. doi: 10.1186/s12913-017-2796-9 (PMC5746011; doi:10.1186/s12913-017-2796-9)
Supplement: Supplementary file 1 — Household survey. (PDF 839 kb) [file 12913_2017_2796_MOESM1_ESM.pdf]

IIHMR

MAMTA
Health Institute for Mother and Child

Medtronic Foundation India NCD Program

Situational analysis of Non Communicable Diseases

INTRODUCTION AND INFORMED CONSENT

Namaste, My name is _______________. We are conducting a situational analysis of Non Communicable Diseases in Shimla and Udaipur districts. This survey is being conducted by MAMTA Health Institute for Mother and Child, New Delhi. Department of Community Medicine, I.G. Medical College, Shimla and R.N.T. Medical College, Udaipur are the local partners in the study.

The main purpose of the survey is to assess the current situation of the NCDs among the population of aged 15 years and above, especially focusing on Diabetes and Heart Diseases. We will use the findings of the assessment for designing intervention strategies to improve the public and private health services. As part of this study, we would ask you some questions related to your lifestyle choices, medical history and treatment seeking behavior. The whole process would take about 30-35 minutes to complete. We would appreciate your participation in this survey.

Everything that you report during the interview will be kept strictly confidential.

Participation in this survey is voluntary and you can choose not to answer any question and can stop the interview at any time. There will not be any consequences if you decide to drop out of the study. However, we hope that you will participate in the study since your participation is important to the results.

At this time, do you want to ask me anything about the study or intervention?

ANSWER ANY QUESTIONS AND ADDRESS RESPONDENT'S CONCERNS.

In case you need more information about the study, you may contact:

MAMTA HEALTH INSTITUTE FOR MOTHER AND CHILD
B-5, G.K. Enclave -II, New Delhi-110048
Ph: 011-29220210, 29220220

I would request you to give your consent. Signature of the Respondent/Guardian

**RESPONDENT AGREES**	1 →	**BEGIN INTERVIEW**
**RESPONDENT DOES NOT AGREE**	2 →	**END**

Name and Signature of Investigator with date

Name and Signature of the Supervisor with date

**Serial No:**	**Date of Interview: ____/_____/_______**
**Name of the Respondent:**	**Name of the Head of Household:**
**House Hold no:**	**Landmark:**
**Village/Ward name:**	**Tehsil/Town name :**
**District name :**	**Respondent's Contact No:**

**SECTION – 1: SOCIO-DEMOGRAPHIC PROFILE**<br>**Please circle the appropriate response**			**SKIP TO**
1.	Age (in completed years)	____________________(in years)	
2.	Sex	1. Male<br>2. Female	
3.	Religion	1. Hindu<br>2. Muslim<br>3. Christian<br>4. Other (Specify)_____________________	
4.	Caste	1. Schedule caste<br>2. Schedule tribe<br>3. Other Backward class<br>4. Others	
5.	Qualification	1. Illiterate/no formal education<br>2. 1st -5th class<br>3. 6th -8th class<br>4. 9th -10th class<br>5. 10th -12th class<br>6. Graduate and Above	
6.	Marital Status	1. Never Married<br>2. Currently Married<br>3. Married but *gauna* not performed<br>4. Widow/ Divorced/ Separated	
7.	Occupation Status	1. Professional (administrative/managerial)<br>2. Clerical/Sales<br>3. Service<br>4. Shopkeeper/Business<br>5. Skilled/unskilled manual worker<br>6. Agriculture worker<br>7. Domestic worker<br>8. Unemployed<br>9. Student<br>10. Housewife<br>11. Other **(SPECIFY)**……………………………………	
8.	Family Monthly Income (Rs.)	____________________(in Rs.)	

**SECTION – 2: NCD RISK FACTORS**			
**PART – A: DIETARY HABITS**			
**<u>The next 5 questions are about foods you eat, drinking and eating habits</u>.**			
9.	Type of Diet	1. Vegetarian<br>2. Eggetarian<br>3. Non-Vegetarian	

10.	During the past 30 days, how many times per day did you usually eat fruit, such as apple, mango, banana, pineapple, papaya, jackfruit, guava, or chikoo?	1. I did not eat fruit during the past 30 days<br>2. 1 time per day<br>3. 2 times per day<br>4. 3 times per day<br>5. 4 times per day<br>6. 5 or more times per day<br>7. Other **(SPECIFY)**__________________	
11.	During the past 30 days, how many times per day did you usually eat vegetables, such as cauliflower, ladyfinger, pumpkin, brinjal, cabbage, spinach, peas, tomato, cucumber or beans?	1. I did not eat vegetables during the past 30 days<br>2. 1 time per day<br>3. 2 times per day<br>4. 3 times per day<br>5. 4 times per day<br>6. 5 or more times per day<br>7. Other (specify) ________________________	
12.	During the past 30 days, how many times per day did you usually drink carbonated soft drinks, such as Cola or cold drinks?	1. I did not drink carbonated soft drinks during the past 30 days<br>2. 1 time per day<br>3. 2 times per day<br>4. 3 times per day<br>5. 4 times per day<br>6. 5 or more times per day<br>7. Other **(SPECIFY)**________________________	
13.	During the past 7 days, on how many days did you eat at a fast food restaurant, or at those serving quick meals (e.g. Samosas, patties, burgers, noodles, tikkis, or ice creams)?	1. 0 days<br>2. 1 day<br>3. 2 days<br>4. 3 days<br>5. 4 days<br>6. 5 days<br>7. 6 days<br>8. 7 days	
**PART – B: PHYSICAL ACTIVITY**			
i. **Physical activity is any activity that increases your heart and respiration rate. Physical activity can be done in sports, playing with friends, or walking for examples running, biking, dancing, or football. ADD UP ALL THE TIME YOU SPEND IN PHYSICAL ACTIVITY EACH DAY.**			
14.	During a **usual** week, on how many days are you physically active for a total of at least 60 minutes per day?	1. 0 days<br>2. 1 day<br>3. 2 days<br>4. 3 days<br>5. 4 days<br>6. 5 days<br>7. 6 days<br>8. 7 days	
15.	How much time do you spend during a **usual** day sitting and watching television, playing computer games, talking with friends, or doing other sitting activities, such as listening to music?	1. Less than 1 hour per day<br>2. 1 to 2 hours per day<br>3. 3 to 4 hours per day<br>4. 5 to 6 hours per day<br>5. 7 to 8 hours per day<br>6. More than 8 hours per day	
16.	Do you regularly practice yogic exercise/yoga?	1. Yes<br>2. No →	→ PART C
17.	If yes, how many days in a week?	1. 0 days<br>2. 1 day<br>3. 2 days<br>4. 3 days	

		5. 4 days<br>6. 5 days<br>7. 6 days<br>8. 7 days	
18.	How much time do you spend doing yoga in a typical day? (in minutes)	___________(in minutes)	
**PART – C: TOBACCO USE**			
**<u>The next 12 questions are about cigarette/bidi and other tobacco use.</u>**			
19.	Which of your family members and friends use tobacco in any form?<br><br>***(More than one answer is possible)***<br>***Please circle all that apply***	A. None<br>B. My father or male guardian<br>C. My mother or female guardian<br>D. Both parents<br>E. Husband/Wife<br>F. Brothers and/or sisters<br>G. Any other family member in same household<br>H. Friends<br>I. I do not know	
20.	Have you ever used tobacco in any form? (smoking, chew, snuff)	1. Smoking<br>2. Chewing<br>3. Never used →	→ PART D
21.	If yes, how old were you when you used tobacco for the first time?	________________(In years)	
22.	What was the reason for initiation of tobacco use?<br>***(More than one answer is possible)***	A. I wanted to see what would happen if I smoke/chew tobacco.<br>B. I just felt tempted to smoke/chew tobacco.<br>C. Friend offered<br>D. I saw parent/relative/sibling chew/smoke tobacco.<br>E. I saw my favorite actor/actress chew/smoke tobacco.<br>F. When I saw tobacco (any form) I just had to try.<br>G. Others **(SPECIFY)**__________________________	
23.	Do you currently smoke?	1. Yes<br>2. No →	→ Q26
24.	Have you ever attempted to stop smoking cigarettes/bidis?	1. Yes<br>2. No →	→ Q26
25.	At which age did you stop smoking or attempt to stop smoking cigarettes/ bidis?	_______________(In years)	
26.	Do you currently chew tobacco?	1. Yes<br>2. No →	→Q29
27.	Have you ever attempted to stop chewing tobacco?	1. Yes<br>2. No →	→ Q29
28.	At which age did you stop chewing or attempt to stop chewing tobacco?	_______________(In years)	
29.	**(Ask only if Q. 24 or Q27 is "1" otherwise SKIP TO PART-D)**<br><br>What were the reasons you wanted to stop tobacco use?<br>***(More than one answer is possible)***	A. Will power<br>B. Felt weakness/ breathless/chronic cough<br>C. Advised by family member/Relatives/friends<br>D. Fear about Health problem<br>E. Suffering from health problem<br>F. Advised by Doctor/Health worker<br>G. Other **(SPECIFY)**_________________________	
30.	What specific cessation method you used/using to quit tobacco use?	1. Medicines<br>2. Counseling/Advice<br>3. Other methods_________________	

PART – D: ALCOHOL USE			
**The next 9 questions ask about the role of alcohol.**			
31.	Which of your family members and friends drink alcohol?<br>***(More than one answer is possible)***	A. None<br>B. My father or male guardian<br>C. My mother or female guardian<br>D. Both parents<br>E. Husband/Wife<br>F. Brothers and/or sisters<br>G. Any other family member in same household<br>H. Friends<br>I. I do not know	
32.	Have you ever used alcohol?	1. Yes<br>2. No →	→ SECTION 3
33.	How old were you when you had first drink?	_______________(In years)	
34.	What were the reasons for initiation of alcohol?<br>***(More than one answer is possible)***	A. I wanted to see what would happen if I drink alcohol.<br>B. I just felt tempted to drink.<br>C. Friend offered<br>D. I felt worried or tense about my relationship with someone else.<br>E. I saw parent/relative/sibling drinking<br>F. I saw my favorite actor/actress drinking<br>G. I felt angry or frustrated, either with myself or because things were not going my way.<br>H. When I saw alcohol I just had to try.<br>I. Others **(SPECIFY)**________________________	
35.	Do you currently drink alcohol?	1. Yes<br>2. No →	→Q37
36.	Have you ever attempted to quit alcohol?	1. Yes<br>2. No →	→Q40
37.	At which age did you stop drinking alcohol or attempt to quit alcohol use?	_______________(In years)	
38.	**(Ask, if Q35 is "2" or Q.36 is "1". Otherwise SKIP TO SECTION 3)**<br>What were the reasons to stop drinking or attempted to stop drinking alcohol use?<br>***(More than one answer is possible)***	1. Will power<br>2. Felt weakness/ breathless/chronic cough<br>3. Advised by family member/Relatives/friends<br>4. Fear about Health problem<br>5. Suffering from health problem<br>6. Advised by Doctor/Health worker<br>7. Other **(SPECIFY)**_____________________	
39.	What specific cessation method you used/using to quit alcohol?	1. Medicines<br>2. Counseling/Advice<br>3. Other methods_________________	
**SECTION -3: KNOWLEDGE ABOUT KEY NCDs**			
40.	Have you ever heard about the following disease/health conditions?<br>**(Read options)**<br>***(More than one answer is possible)***	A. Hypertension (High Blood Pressure)<br>B. Diabetes (High Blood Sugar)<br>C. Heart Disease<br>D. Stroke (Paralytic Attack)<br>E. Chronic Kidney Disease<br>F. None →	→ Q52

41.	From where/whom did you got the information about the disease/health conditions?<br>***(More than one answer is possible)***<br>***(Please put 1 for YES , 2 for NO & 3 if NOT APPLICABLE)***		

**Source of information**	High Blood Pressure	High Blood Sugar	Heart Disease	Paralysis Attack	Chronic Kidney Disease
TV/Radio					
School teacher					
Friend/Relatives/Sibling/Parents					
Husband/Wife					
Book/Newspaper/Magazine Poster/Banner					
Doctor from Public Health Facility					
ANM/Health Worker					
ASHA/Anganwadi Worker					
Doctor from Private Health Facility					
Community events (drama/rally)					
Pharmacist					
Others (SPECIFY)_____________					

	**If the answer is reported to any of the diseases in Q. 40, then ask the respective questions from Q42 to Q51. Else go to Q52.**		
42.	What are the symptoms of High Blood Pressure you are aware of?<br>***(More than one answer is possible)***	A. Severe headaches<br>B. Severe anxiety<br>C. Shortness of breath<br>D. Nosebleeds<br>E. Blood Spots in eyes<br>F. Facial flushing<br>G. Dizziness<br>H. Others **(SPECIFY)**_______________<br>I. Do not know/Not aware	
43.	What are the risk factors of High Blood Pressure you are aware of?<br>***(More than one answer is possible)***	A. Family history<br>B. Lack of physical activity<br>C. High salt intake<br>D. Unhealthy diet<br>E. Overweight and obesity<br>F. Stress<br>G. Drink alcohol<br>H. Others **(SPECIFY)**_______________<br>I. Do not know/Not aware	
44.	What are the symptoms of Diabetes you are aware of?<br>***(More than one answer is possible)***	A. Increased thirst<br>B. Increased appetite<br>C. Fatigue<br>D. Increased Unrination, especially at night<br>E. Weight loss<br>F. Blurred vision<br>G. Sores that do not heal<br>H. Others **(SPECIFY)**_______________<br>I. Do not know/Not aware	
45.	What are the risk factors of Diabetes you are aware of?<br>***(More than one answer is possible)***	A. Family history<br>B. Lack of Physical Activity<br>C. Unhealthy diet<br>D. Overweight and obesity<br>E. Stress<br>G. Drink alcohol<br>H. Others **(SPECIFY)**_______________<br>I. Do not know/Not aware	

46.	What are the symptoms of Heart Disease you are aware of?<br>***(More than one answer is possible)***	A. Undue fatigue<br>B. Pain or discomfort in the jaw, neck, or back.<br>C. Feeling weak, light-headed, or faint.<br>D. Chest pain or discomfort.<br>E. Pain or discomfort in arms or shoulder.<br>F. Shortness of breath<br>G. Others **(SPECIFY)**_______________<br>H. Do not know/Not aware	
47.	What are the risk factors of Heart Disease you are aware of?<br>***(More than one answer is possible)***	A. Smoking<br>B. High blood pressure<br>C. High blood cholesterol<br>D. Diabetes<br>E. Being overweight or obese<br>F. Unhealthy diet<br>G. Physical inactivity<br>H. Stress<br>I. Drink alcohol<br>J. Others **(SPECIFY)**_______________<br>K. Do not know/Not aware	
48.	What are the symptoms of chronic kidney disease you are aware of?<br>***(More than one answer is possible)***	A. Urinate less than normal.<br>B. Have swelling on feet / face.<br>C. Feel very tired.<br>D. Lose your appetite or have an unexpected weight loss<br>E. Feel nauseated or vomit.<br>F. Be either very sleepy or unable to<br>G. Have or trouble thinking straight.<br>H. Others **(SPECIFY)**_______________	
49.	What are the risk factors of chronic kidney disease you are aware of?<br>***(More than one answer is possible)***	A. Diabetes<br>B. High blood pressure<br>C. Kidney infection / stone<br>D. Heart disease<br>E. Smoking<br>F. Obesity<br>G. High cholesterol<br>H. Family history of kidney disease<br>I. Age 65 or older<br>J. Others **(SPECIFY)**_______________<br>K. Do not know/Not aware	
50.	What are the symptoms of stroke you are aware of?<br>***(More than one answer is possible)***	A. Sudden numbness or weakness of the face, arm, or leg.<br>B. Sudden confusion or trouble speaking or understanding others.<br>C. Sudden trouble seeing in one or both eyes.<br>D. Sudden dizziness, trouble walking, or loss of balance or coordination.<br>E. Sudden severe headache with no known cause.<br>F. Paralysis attack<br>G. Others **(SPECIFY)**_______________<br>H. Do not know/Not aware	
51.	What are the risk factors of stroke you are aware of?<br>***(More than one answer is possible)***	A. High Blood Pressure<br>B. High Cholesterol<br>C. Heart disease<br>D. Diabetes<br>E. Overweight and obesity<br>F. Previous stroke	

		G. Others **(SPECIFY)**________________<br>H. Do not know/Not aware	
52.	Have you ever been told by doctor that you have any of the following disease/conditions?<br>***(Read options)***<br>***(More than one answer is possible)***	A. Hypertension (High Blood Pressure)<br>B. Diabetes (High Blood Sugar)<br>C. Hyperlipidemia (High cholesterol)<br>D. Heart Disease<br>E. Stroke (Paralytic Attack)<br>F. Chronic Kidney Disease<br>G. None	
53.	**ONLY IN CASE OF EVER MARRIED WOMEN WHO HAVE EVER EXPERIENCED ATLEAST ONE PREGNANCY**<br>Have you ever been told by doctor that you have any of the following disease?<br>**(Read options)**<br>***(More than one answer is possible)***	A. High Blood Pressure during pregnancy<br>B. High Blood Sugar during pregnancy<br>C. Hyperlipidemia (High cholesterol)<br>D. Heart Disease<br>E. Stroke (Paralytic Attack)<br>F. Chronic Kidney Disease<br>G. None	
**If the answer is reported to any of the choices in Q. 52 or Q 53, then GO TO SECTION-4 related disease part 'OTHERWISE' GO TO SECTION-5.**			
**SECTION 4: MEDICAL HISTORY**			
**PART –A : HYPERTENSION (HIGH BLOOD PRESSURE)**			
54.	At what age were you diagnosed for Hypertension (high Blood Pressure)?	__________________(in Years)	
55.	Where were you diagnosed?<br>***(More than one answer is possible)***	**Public Sector**<br>A. Medical College / Tertiary Hospital<br>B. District Hospital<br>C. Dispensary<br>D. UHC/UHP/UFWC<br>E. CHC/ Rural Hospital<br>F. PHC<br>G. Sub center<br>H. AYUSH Hospital/Clinic<br>**Private Sector**<br>I. NGO/Trust Hospital/Clinic<br>J. Specialist Hospital/Clinic<br>K. AYUSH Hospital/Clinic<br>L. Other **(SPECIFY)**____________________________	
56.	What was the reason to prefer/decide to visit the health facility for diagnosis?<br>***(More than one answer is possible)***	A. Referred by doctor<br>B. Referred by ANM / Health Worker<br>C. Facilitated/Motivated by NGO/CBO<br>D. Motivated by Parents/Siblings<br>E. Self Motivated<br>F. Availability of Specialist<br>G. Quality of Services<br>H. Other **(SPECIFY)**____________________________	
57.	Is your family member aware that you are suffering from the disease / health problem?	1. Yes<br>2. No	
58.	Have you consulted anyone for advice or treatment for this problem before visiting this facility?	1. Yes<br>2. No →	→ Q60
59.	To whom did you consult before taking decision to visit the health facility?<br>***(More than one answer is possible)***	A. Parents/Siblings<br>B. Relatives/Friends<br>C. Husband/Wife<br>D. ANM/Health Worker/Nurse<br>E. Doctor from PHC/CHC/Rural Hospital<br>F. Doctor from Private Health Facility<br>G. Doctor from AYUSH Hospital/Clinic from	

		Public/Private Sector<br>H. Other **(SPECIFY)**___________________________	
60.	Who has taken the final decision regarding the visit to the health facility?	1. Self<br>2. Husband/Wife<br>3. Parents<br>4. Jointly with family members<br>5. Other **(SPECIFY)**______________________	
61.	Do you receive adequate support from your family member in prevention/management of the disease?	1. Yes<br>2. No→	→Q63
62.	What kind of support do you receive from your family member in prevention/management of the disease?<br>***(More than one answer is possible)***	A. Facilitate / Motivate for regular check-up /consultation with the doctor<br>B. Motivate to take regular medicines<br>C. Financial support<br>D. Care during critical illness<br>E. Frequent support to adopt healthy lifestyle<br>F. Monitoring clinical symptoms<br>G. Help in making decision regarding treatment<br>H. Support to cope-up from the stress<br>I. Other **(SPECIFY)**______________________	
63.	What are the treatments/advice you are currently receiving for raised (high) blood pressure?<br>***(More than one answer is possible)***	A. Allopathic-Oral drug (medication)<br>B. AYUSH-Oral drug (medication)<br>C. Injectables<br>D. Salt restriction<br>E. Special Prescribed diet<br>F. Advice or treatment to lose weight<br>G. Advise to start or do more exercises<br>H. Stop/reduce tobacco/alcohol consumption<br>I. None<br>J. Others **(SPECIFY)**________________	
64.	Who prescribed the above treatment/advice?<br>***(More than one answer is possible)***	A. Allopathic Doctor<br>B. AYUSH Doctor<br>C. Pharmacist<br>D. ANM/Health worker<br>E. Friend/Neighbour<br>F. Other **(SPECIFY)**___________________	
65.	What type of treatment you have tried for high blood pressure in the past?<br>***(More than one answer is possible)***	A. Allopathic<br>B. Ayurvedic<br>C. Yoga<br>D. Unani<br>E. Sidha<br>F. Homeopathy<br>G. Home remedies<br>H. Others **(SPECIFY)** ________________________	
66.	How regular are you in taking your medicines as prescribed?	1. As prescribed by the doctor/health worker →<br>2. Take medicine only when I feel unwell<br>3. Medicine discontinued<br>4. Never taken any medication	→Q68
67.	What was the reason of discontinuation of the medicines?<br>***(More than one answer is possible)***	A. Cannot tolerate the medicines<br>B. I have recovered<br>C. Too expensive<br>D. Complicated procedure for care seeking<br>E. Do not trust medical care<br>F. No reason<br>G. Other (**SPECIFY**)________________________	

68.	How often you visit your doctor?	1.As prescribed by the doctor/health worker →<br>2.When I feel unwell<br>3. Visits discontinued<br>4. Other (**SPECIFY**)_________________________	→Q70
69.	What was the reason of Discontinuation of the regular visit to doctor?<br>***(More than one answer is possible)***	A. Cannot afford<br>B. Cannot tolerate the medicines<br>C. I have recovered<br>D. No specialist Doctor available<br>E. Long distance<br>F. Too expensive<br>G. Complicated procedure for care seeking<br>H. Long waiting time<br>I. Too sick to visit the facility<br>J. Do not trust medical care<br>K. Not aware about health facility<br>L. No reason<br>M. Other (**SPECIFY**)_________________________	
70.	List the expenditure incurred towards the condition (high blood pressure).		

**Expenditure**	**Amount spent in Rupees (on an average in a month)**
Regular visit to doctor (fees)	
Transportation cost to visit doctor	
Medication (Average amount spent per month for the above mentioned condition)	
Regular laboratory tests	
Others(specify)	
**TOTAL EXPENDITURE INCURRED**	

71.	What was the source of payment for the above expenditure?<br>***(More than one answer is possible)***	A. Own saving<br>B. Family member paid<br>C. Employer paid<br>D. Borrowed from friend, relative & employer<br>E. Borrowed from bank<br>F. Sold house, land or other assests<br>G. Health Insurance<br>H. Others **(SPECIFY)**________________	
72.	To what extent you were satisfied with the services / treatment attained from the facility?	1. Very Satisfied<br>2. Satisfied<br>3. Indifferent<br>4. Dissatisfied<br>5. Very Dissatisfied	
**PART –B : DIABETES (HIGH BLOOD SUGAR)**			
73.	At what age did you have your Diabetes (high Blood Sugar)?	_______________(In years)	
74.	Where were you diagnosed?<br>***(More than one answer is possible)***	**Public Sector**<br>A. Medical College / Tertiary Hospital<br>B. District Hospital<br>C. Dispensary<br>D. UHC/UHP/UFWC<br>E. CHC/ Rural Hospital<br>F. PHC<br>G. Sub center<br>H. AYUSH Hospital/Clinic<br>**Private Sector**<br>I. NGO/Trust Hospital/Clinic<br>J. Specialist Hospital/Clinic	

		K. AYUSH Hospital/Clinic<br>L. Other **(SPECIFY)**___________________________	
75.	What was the reason to prefer/decide to visit the health facility for diagnosis?<br>***(More than one answer is possible)***	A. Referred by doctor<br>B. Referred by ANM / Health Worker<br>C. Facilitated/Motivated by NGO/CBO<br>D. Motivated by Parents/Siblings<br>E. Self Motivated<br>F. Availability of Specialist<br>G. Quality of Services<br>H. Other **(SPECIFY)**___________________________	
76.	Is your family member aware that you are suffering from the disease / health problem?	1. Yes<br>2. No	
77.	Have you consulted anyone for advice or treatment for this problem before visiting this facility?	1. Yes<br>2. No	
78.	To whom did you consult before taking decision to visit the health facility?	1. Parents/Siblings<br>2. Relatives/Friends<br>3. Husband/Wife<br>4. ANM/Health Worker<br>5. Doctor from PHC/CHC/Rural Hospital<br>6. Doctor from Private Health Facility<br>7. Doctor from AYUSH Hospital/Clinic from Public/Private Sector<br>8. Other **(SPECIFY)**___________________________	
79.	Who has taken the final decision regarding the visit to the health facility?	1. Self<br>2. Husband/Wife<br>3. Parents<br>4. Jointly with family members<br>5. Other **(SPECIFY)**_______________________	
80.	Do you receive adequate support from your family member in prevention/management of the disease?	1. Yes<br>2. No →	→ Q82
81.	What kind of support do you receive from your family member in prevention/management of the disease?	A. Facilitate / Motivate for regular check-up /consultation with the doctor<br>B. Motivate to take regular medicines<br>C. Financial support<br>D. Care during critical illness<br>E. Frequent support to adopt healthy lifestyle<br>F. Monitoring clinical symptoms<br>G. Help in making decision regarding treatment<br>H. Support to cope-up from the stress<br>I. Other **(SPECIFY)**_______________________	
82.	What are the treatments/advice you are currently receiving for raised (high) blood sugar?<br>***(More than one answer is possible)***	A. Allopathic-Oral drug (medication)<br>B. AYUSH-Oral drug (medication)<br>C. Injectables<br>D. Sugar restriction<br>E. Special Prescribed diet<br>F. Advice or treatment to lose weight<br>G. Advise to start or do more exercises<br>H. Stop/ reduce Tobacco/alcohol consumption<br>I. None<br>J. Other **(SPECIFY)**____________________	
83.	Who prescribed the above treatment/advice?<br>***(More than one answer is possible)***	A. Allopathic Doctor<br>B. AYUSH Doctor<br>C. Pharmacist<br>D. ANM/Health worker	

		E. Friend/Neighbour<br>F. Other **(SPECIFY)**___________________	
84.	What type of treatment you have tried for high blood sugar in the past?<br>***(More than one answer is possible)***	A. Allopathic<br>B. Ayurvedic<br>C. Yoga<br>D. Unani<br>E. Sidha<br>F. Homeopathy<br>G. Home remedies<br>H. Others **(SPECIFY)**________________	
85.	How regular are you in taking your medicines?	1. As prescribed by the doctor/health worker →<br>2. Take medicine only when I feel unwell<br>3. Medicine discontinued<br>4. Never taken any medication	→Q87
86.	What was the reason of Discontinuation?<br>***(More than one answer is possible)***	A. Cannot tolerate the medicines<br>B. I have recovered<br>C. Too expensive<br>D. Complicated procedure for care seeking<br>E. Do not trust medical care<br>F. No reason<br>G. Other (**SPECIFY**)________________________	
87.	How often you visit your doctor?	1. As prescribed by the doctor/health worker →<br>2. When I feel unwell<br>3. Visits discontinued<br>4. Other (**SPECIFY**)________________________	→ Q89
88.	What was the reason of Discontinuation of the regular visit to doctor?<br>***(More than one answer is possible)***	A. Cannot afford<br>B. Cannot tolerate the medicines<br>C. I have recovered<br>D. No specialist Doctor available<br>E. Long distance<br>F. Too expensive<br>G. Complicated procedure for care seeking<br>H. Long waiting time<br>I. Too sick to visit the facility<br>J. Do not trust medical care<br>K. Not aware about health facility<br>L. No reason<br>M. Other (**SPECIFY**)________________________	
89.	List the expenditure incurred towards the condition (high blood sugar) in a month.		

**Expenditure**	**Amount spent in Rupees (on an average in a month)**
Regular visit to doctor (fees)	
Transportation cost to visit doctor	
Medication (Average amount spent per months for the above mentioned condition)	
Dialysis	
Regular laboratory tests	
Others **(SPECIFY)**________________	
**TOTAL EXPENDITURE INCURRED**	

90.	What was the source of payment for the above expenditure?<br>***(More than one answer is possible)***	A. Own saving<br>B. Family member paid<br>C. Employer paid<br>D. Borrowed from friend, relative & employer<br>E. Borrowed from bank<br>F. Sold house, land or other assests	

		G. Health Insurance<br>H. Others **(SPECIFY)________________**	
91.	To what extent you were satisfied with the services / treatment attained from the facility?	1.Very Satisfied<br>2. Satisfied<br>3. Indifferent<br>4. Dissatisfied<br>5. Very Dissatisfied	
**COMPLICATIONS**			
**B-I : CHRONIC KIDNEY DISEASE**			
92.	At what age were you diagnosed for chronic kidney disease?	_______________(In years)	
93.	Where were you diagnosed?<br>***(More than one answer is possible)***	**Public Sector**<br>A. Medical College / Tertiary Hospital<br>B. District Hospital<br>C. Dispensary<br>D. UHC/UHP/UFWC<br>E. CHC/ Rural Hospital<br>F. PHC<br>G. Sub center<br>H. AYUSH Hospital/Clinic<br>**Private Sector**<br>I. NGO/Trust Hospital/Clinic<br>J. Specialist Hospital/Clinic<br>K. AYUSH Hospital/Clinic<br>L. Other **(SPECIFY)__________________________**	
94.	What was the reason to prefer/decide to visit the health facility for diagnosis?<br>***(More than one answer is possible)***	A. Referred by doctor<br>B. Referred by ANM / Health Worker<br>C. Facilitated/Motivated by NGO/CBO<br>D. Motivated by Parents/Siblings<br>E. Self Motivated<br>F. Availability of Specialist<br>G. Quality of Services<br>H. Other **(SPECIFY)___________________________**	
95.	Is your family member aware that you are suffering from the disease / health problem?	1. Yes<br>2. No	
96.	Have you consulted anyone for advice or treatment for this problem before visiting this facility?	1. Yes<br>2. No	
97.	To whom did you consult before taking decision to visit the health facility?	1. Parents/Siblings<br>2. Relatives/Friends<br>3. Husband/Wife<br>4. ANM/Health Worker<br>5. Doctor from PHC/CHC/Rural Hospital<br>6. Doctor from Private Health Facility<br>7. Doctor from AYUSH Hospital/Clinic from Public/Private Sector<br>8. Other**(SPECIFY)________________________**	
98.	Who has taken the final decision regarding the visit to the health facility?	1. Self<br>2. Husband/Wife<br>3. Parents<br>4. Jointly with family members<br>5. Other **(SPECIFY)_______________________**	
99.	What are the treatments/advice you are currently receiving for Chronic Kidney Disease?	A. Allopathic-Oral drug (medication)<br>B. AYUSH-Oral drug (medication)<br>C. Dialysis	

	(More than one answer is possible)	D. Injectables<br>E. Sugar restriction<br>F. Special Prescribed diet<br>G. Advice or treatment to lose weight<br>H. Advise to start or do more exercises<br>I. Stop/reduce Tobacco/alcohol consumption<br>J. None<br>K. Other **(SPECIFY)**____________________	
100.	**Ask only, if Q. 99 is 'C'.**<br>How frequently do you require dialysis?	1. Once a day<br>2. Once a week<br>3. Twice in a week<br>4. Once a month<br>5. Twice in a month	
101.	Who prescribed the above treatment/advice?<br>***(More than one answer is possible)***	A. Allopathic Doctor<br>B. AYUSH Doctor<br>C. Pharmacist<br>D. ANM/Health worker<br>E. Friend/Neighbour<br>F. Other **(SPECIFY)**____________________	
102.	What type of treatment you have tried for chronic kidney disease in the past?<br>***(More than one answer is possible)***	A. Allopathic<br>B. Ayurvedic<br>C. Yoga<br>D. Unani<br>E. Sidha<br>F. Homeopathy<br>G. Home remedies<br>H. Other **(SPECIFY)**___________________________	
103.	How regular are you in taking your medicines?	1. As prescribed by the doctor/health worker →<br>2. Take medicine only when I feel unwell<br>3. Medicine discontinued<br>4. Never taken any medication	→Q105
104.	What was the reason of Discontinuation?<br>***(More than one answer is possible)***	A. Cannot tolerate the medicines<br>B. I have recovered<br>C. Too expensive<br>D. Complicated procedure for care seeking<br>E. Do not trust medical care<br>F. No reason<br>G. Other (**SPECIFY**)_________________________	
105.	How often you visit your doctor?	1. As prescribed by the doctor/health worker →<br>2. When I feel unwell<br>3. Visit discontinued<br>4. Other (**SPECIFY**)_________________________	→Q107
106.	What was the reason of Discontinuation of the regular visit to doctor?<br>*(More than one answer is possible)*	A. Cannot afford<br>B. Cannot tolerate the medicines<br>C. I have recovered<br>D. No specialist Doctor available<br>E. Long distance<br>F. Too expensive<br>G. Complicated procedure for care seeking<br>H. Long waiting time<br>I. Too sick to visit the facility<br>J. Do not trust medical care<br>K. Not aware about health facility<br>L. No reason<br>M. Other **(SPECIFY)**_________________________	
**B-II: FOOT ULCERS AND AMPUTATION**			

107.	Have you ever had a non-healing ulcer/sore in the foot that took more than 4 weeks to heal?	1. Yes<br>2. No	
108.	Have you had an amputation due to complication of diabetes?	1. Yes<br>2. No →	→ Part B III
109.	If Yes when?	Month__________ Year_____________	
110.	Level of amputation	1. Toe<br>2. Below ankle<br>3. Below knee<br>4. Above knee	
111.	Are you taking any preventive measures to avoid future complication?	1. Yes<br>2. No →	→ Part B III
112.	What are the preventive measures you are taking?	1. Wear footwear always<br>2. Attend injuries immediately<br>3. Keep blood sugar level under control<br>4. Other **(SPECIFY)**_________________________	
**B-III : EYES**			
113.	Do you have difficulty with your eyesight other than your ordinary power glasses (spectacles)?	1. Yes<br>2. No →	→ Part D
114.	Were you told that your poor eyesight is due to complications of diabetes?	1. Yes<br>2. No →	→ Part D
115.	What was the diagnosis?<br>(Confirm from records)	1. Diabetic Retinopathy<br>2. Cataract<br>3. Other Others **(SPECIFY)**________________	
116.	Have you undergone laser therapy (Photocoagulation) at anytime?	1. Yes<br>2. No	
117.	Do you have medical records or prescriptions?	1. Yes<br>2. No	
118.	List the expenditure incurred towards the condition (chronic kidney disease) in a month.		

**Expenditure**	**Amount spent in Rupees (on an average in a month)**
Regular visit to eye doctor (fees)	
Transportation cost to visit doctor	
Medication (Average amount spent per months for the above mentioned condition)	
Regular laboratory tests/ Procedure	
Laser treatment	
Others(specify)	
**TOTAL EXPENDITURE INCURRED**	

119.	What was the source of payment for the above expenditure?<br>***(More than one answer is possible)***	A. Own saving<br>B. Family member paid<br>C. Employer paid<br>D. Borrowed from friend, relative & employer<br>E. Borrowed from bank<br>F. Sold house, land or other assests<br>G. Health Insurance<br>H. Others **(SPECIFY)**_______________	
**PART – C : HEART DISEASE**			
120.	At what age were you diagnosed with Heart disease?	______________(in Years)	

121.	What did the doctor say it was?<br><br>***(More than one answer is possible)***<br><br>***(If "A" go to Q 122 otherwise Skip to Q 125)***	A. Heart attack<br>B. Pain in chest (Angina)<br>C. Heart failure<br>D. Valve disease<br>E. Hole in the heart (congenital heart disease)<br>F. Others **(SPECIFY)**_______________<br>G. Not informed about the nature of the problem	
122.	If had heart attack, at what age did you have the 1st heart attack?	________________ (in Years)	
123.	Were you hospitalized for treatment?	1. Yes<br>2. No	
124.	Did you have any repeat attacks?	1. Yes<br>2. No	
125.	Where were you diagnosed?<br>***(More than one answer is possible)***	**Public Sector**<br>A. Medical College / Tertiary Hospital<br>B. District Hospital<br>C. Dispensary<br>D. UHC/UHP/UFWC<br>E. CHC/ Rural Hospital<br>F. PHC<br>G. Sub centre<br>H. AYUSH Hospital/Clinic<br>**Private Sector**<br>I. NGO/Trust Hospital/Clinic<br>J. Specialist Hospital/Clinic<br>K. AYUSH Hospital/Clinic<br>L. Other **(SPECIFY)**________________________	
126.	What was the reason to prefer/decide to visit the health facility for diagnosis?<br>***(More than one answer is possible)***	A. Referred by doctor<br>B. Referred by ANM / Health Worker<br>C. Facilitated/Motivated by NGO/CBO<br>D. Motivated by Parents/Siblings<br>E. Self Motivated<br>F. Availability of Specialist<br>G. Quality of Services<br>H. Other **(SPECIFY)**______________________	
127.	Is your family member aware that you are suffering from the disease / health problem?	1. Yes<br>2. No	
128.	Have you consulted anyone for advice or treatment for this problem before visiting this facility?	1. Yes<br>2. No	
129.	To whom did you consult before taking decision to visit the health facility?	1. Parents/Siblings<br>2. Relatives/Friends<br>3. Husband/Wife<br>4. ANM/Health Worker<br>5. Doctor from PHC/CHC/Rural Hospital<br>6. Doctor from Private Health Facility<br>7. Doctor from AYUSH Hospital/Clinic from Public/Private Sector<br>8. Other(**SPECIFY)**______________________	
130.	Who has taken the final decision regarding the visit to the health facility?	1. Self<br>2. Husband/Wife<br>3. Parents<br>4. Jointly with family members<br>5. Other **(SPECIFY)**_____________________	
131.	What are the treatments/advice you are currently receiving for Heart Disease?	A. Allopathic-Oral drug (medication)<br>B. AYUSH-Oral drug (medication)	

	(More than one answer is possible)	C. Injectables<br>D. Salt restriction<br>E. Special Prescribed diet<br>F. Advice or treatment to lose weight<br>G. Advise to start or do more exercises<br>H. Stop/reduce Tobacco/alcohol consumption<br>I. None<br>J. Other **(SPECIFY)**___________________	
132.	Who prescribed the above treatment/advice?<br>***(More than one answer is possible)***	A. Allopathic Doctor<br>B. AYUSH Doctor<br>C. Pharmacist<br>D. ANM/Health worker<br>E. Friend/Neighbour<br>F. Other **(SPECIFY)**___________________	
133.	What type of treatment you have tried for heart disease in the past?<br>***(More than one answer is possible)***	A. Allopathic<br>B. Ayurvedic<br>C. Yoga<br>D. Unani<br>E. Sidha<br>F. Homeopathy<br>G. Home remedies<br>H. Other **(SPECIFY)**_________________________	
134.	How regular are you in taking your medicines as prescribed?	1. As prescribed by the doctor/health worker →<br>2. Take medicine only when I feel unwell<br>3. Medicine discontinued<br>4. Never taken any medication	→Q136
135.	What was the reason of Discontinuation?<br>***(More than one answer is possible)***	A. Cannot tolerate the medicines<br>B. I have recovered<br>C. Too expensive<br>D. Complicated procedure for care seeking<br>E. Do not trust medical care<br>F. No reason<br>G. Other (**SPECIFY**)________________________	
136.	How often you visit your doctor?	1. As prescribed by the doctor/health worker →<br>2. When I feel unwell<br>3. Visits discontinued<br>4. Other (**SPECIFY**)________________________	→Q138
137.	What was the reason of Discontinuation of the regular visit to doctor?<br>*(More than one answer is possible)*	A. Cannot afford<br>B. Cannot tolerate the medicines<br>C. I have recovered<br>D. No specialist Doctor available<br>E. Long distance<br>F. Too expensive<br>G. Complicated procedure for care seeking<br>H. Long waiting time<br>I. Too sick to visit the facility<br>J. Do not trust medical care<br>K. Not aware about health facility<br>L. No reason<br>M. Other **(SPECIFY)**________________________	

138.	List the expenditure incurred towards the condition (heart disease) in a month.		

Expenditure	Amount spent in Rupees (on an average in a month)
Regular visit to doctor (fees)	
Transportation cost to visit doctor	
Medication (Average amount spent per months for the above mentioned condition)	
Regular laboratory tests	
ECG and other advanced investigations	
Others(specify)	
**TOTAL EXPENDITURE INCURRED**	

139.	What was the source of payment for the above expenditure?<br>***(More than one answer is possible)***	A. Own saving<br>B. Family member paid<br>C. Employer paid<br>D. Borrowed from friend, relative & employer<br>E. Borrowed from bank<br>F. Sold house, land or other assests<br>G. Health Insurance<br>H. Other (Specify)	
140.	To what extend you were satisfied with the services / treatment attained from the facility?	1. Very Satisfied<br>2. Satisfied<br>3. Indifferent<br>4. Dissatisfied<br>5. Very Dissatisfied	
**PART – D : STROKE**			
141.	At what age did you have Stroke (Paralysis)?	______________(in Years)	
142.	Is there a residual disability in any part of the body? Does it involve the following?<br>***(More than one answer is possible)***	1. Paralysis of leg/foot<br>2. Paralysis of arm/ hand<br>3. Weakness in leg/ foot<br>4. Weakness in arm/hand<br>5. Paralysis of face<br>6. Defect of speech<br>7. Defect of vision<br>8. Urinary incontinence<br>9. Any other weakness<br>10. Others **(SPECIFY)**______________________	
143.	Where were you diagnosed?<br>***(More than one answer is possible)***	**Public Sector**<br>A. Medical College / Tertiary Hospital<br>B. District Hospital<br>C. Dispensary<br>D. UHC/UHP/UFWC<br>E. CHC/ Rural Hospital<br>F. PHC<br>G. Sub centre<br>H. AYUSH Hospital/Clinic<br>**Private Sector**<br>I. NGO/Trust Hospital/Clinic<br>J. Specialist Hospital/Clinic<br>K. AYUSH Hospital/Clinic<br>L. Other **(SPECIFY)**________________________	
144.	What was the reason to prefer/decide to visit the health facility for diagnosis?<br>***(More than one answer is possible)***	A. Referred by doctor<br>B. Referred by ANM / Health Worker<br>C. Facilitated/Motivated by NGO/CBO	

		D. Motivated by Parents/Siblings<br>E. Self Motivated<br>F. Availability of Specialist<br>G. Quality of Services<br>H. Other **(SPECIFY)**______________________	
145.	Is your family member aware that you are suffering from the disease / health problem?	1. Yes<br>2. No	
146.	Have you consulted anyone for advice or treatment for this problem before visiting this facility?	1. Yes<br>2. No	
147.	To whom did you consult before taking decision to visit the health facility?	1. Parents/Siblings<br>2. Relatives/Friends<br>3. Husband/Wife<br>4. ANM/Health Worker<br>5. Doctor from PHC/CHC/Rural Hospital<br>6. Doctor from Private Health Facility<br>7. Doctor from AYUSH Hospital/Clinic from Public/Private Sector<br>8. Other **(SPECIFY)**_______________________	
148.	Who has taken the final decision regarding the visit to the health facility?	1. Self<br>2. Husband/Wife<br>3. Parents<br>4. Jointly with family members<br>5. Other **(SPECIFY)**______________________	
149.	What are the treatments/advice you are currently receiving for Stroke?<br>***(More than one answer is possible)***	A. Allopathic-Oral drug (medication)<br>B. AYUSH-Oral drug (medication)<br>C. Injectables<br>D. Physiotherapy<br>E. Salt restriction<br>F. Special Prescribed diet<br>G. Advice or treatment to lose weight<br>H. Advise to start or do more exercises<br>I. Stop/reduce Tobacco/alcohol consumption<br>J. None<br>K. Other **(SPECIFY)**___________________	
150.	Who prescribed the above treatment/advice?<br>***(More than one answer is possible)***	A. Allopathic Doctor<br>B. AYUSH Doctor<br>C. Pharmacist<br>D. ANM/Health worker<br>E. Friend/Neighbour<br>F. Other **(SPECIFY)**___________________	
151.	What type of treatment you have tried for Stroke in the past?<br>***(More than one answer is possible)***	A. Allopathic<br>B. Ayurvedic<br>C. Yoga<br>D. Unani<br>E. Sidha<br>F. Homeopathy<br>G. Home remedies<br>H. Other **(SPECIFY)**________________________	
152.	How regular are you in taking your medicines?	1. As prescribed by the doctor/health worker →<br>2. Take medicine only when I feel unwell<br>3. Medicine discontinued<br>4. Never taken any medication	→Q154
153.	What was the reason of Discontinuation?<br>***(More than one answer is possible)***	A. Cannot tolerate the medicines<br>B. I have recovered<br>C. Too expensive	

		D. Complicated procedure for care seeking<br>E. Do not trust medical care<br>F. No reason<br>G. Other (**SPECIFY**)________________________	
154.	How often you visit your doctor?	1. As prescribed by the doctor/health worker →<br>2. When I feel unwell<br>3. Visit discontinued<br>4. Other (**SPECIFY**)________________________	→Q156
155.	What was the reason of Discontinuation of the regular visit to doctor?<br>***(More than one answer is possible)***	A. Cannot afford<br>B. Cannot tolerate the medicines<br>C. I have recovered<br>D. No specialist Doctor available<br>E. Long distance<br>F. Too expensive<br>G. Complicated procedure for care seeking<br>H. Long waiting time<br>I. Too sick to visit the facility<br>J. Do not trust medical care<br>K. Not aware about health facility<br>L. No reason<br>M. Other **(SPECIFY)**________________________	

156. List the expenditure incurred towards the condition (stroke) in a month.

**Expenditure**	**Amount spent in Rupees (on an average in a month)**
Regular visit to doctor (fees)	
Transportation cost to visit doctor	
Medication (Average amount spent per months for the above mentioned condition)	
Regular laboratory tests	
Physiotherapy	
Cost of equipment/divices	
Special investigative procedure e.g. CT Scan, MRI, etc	
Nursing care at home	
Other specify	
**TOTAL EXPENDITURE INCURRED**	

157.	What was the source of payment of the above expenditure?<br>***(More than one answer is possible)***	A. Own saving<br>B. Family member paid<br>C. Employer paid<br>D. Borrowed from friend, relative & employer<br>E. Borrowed from bank<br>F. Sold house, land or other assests<br>G. Health Insurance<br>H. Other**(SPECIFY)**________________________	
158.	To what extent you were satisfied with the services / treatment attained from the facility?	1. Very Satisfied<br>2. Satisfied<br>3. Indifferent<br>4. Dissatisfied<br>5. Very Dissatisfied	
**SECTION – 5: QUALITY OF LIFE**			
159.	Having used various health facilities for different diseases and ailments, do you feel that there is any improvement in your life on the following parameters?<br>***(Read the parameters and its options)***		
**A**	Mobility	1. I have no problems in walking about<br>2. I have some problems in walking about<br>3. I am confined to bed	

**B**	Self-Care (eg. Washing or dressing myself)	1. I have no problems with self-care<br>2. I have some problems washing or dressing myself<br>3. I am unable to wash or dress myself	
**C**	Usual Activities (eg. work, study, housework, family, leisure activities)	1. I have no problem with performing my usual activities<br>2. I have some problems with performing my usual activities<br>3. I am unable to perform my usual activities	
**D**	Pain/ Discomfort	1. I have no pain or discomfort<br>2. I have moderate pain or discomforts<br>3. I have extreme pain or discomforts	
**E**	Anxiety/ Depression	1. I am not anxious or depressed<br>2. I am moderately anxious or depressed<br>3. I am extremely anxious or depressed	

SECTION – 6: FAMILY HISTORY OF DISEASES

160. Family History of Diseases
(More than one answer is possible) (Please record 1=yes, 2=No, 3= Don't know)

Family Members	High Blood Pressure	Heart Disease	Stroke (Paralysis Attack)	High Blood Sugar	Chronic Kidney Disease
None					
Father					
Mother					
Grand Father					
Grand Mother					
Brother					
Sister					
Son					
Daughter					
Any other family member (Specify)					

SECTION -7: HOSPITALIZATION (please verify from the medical records if available with respondent)

161.	Were you hospitalized for any illness since diagnosis of any NCD?	1. Yes<br>2. No →<br>3. Don't remember →	→ End

INSTRUCTION: Please record details for each episode of hospitalization since diagnosis of any NCDs in the following matrix.

S.No.	Date of Hospitalisation	Reason of Hospitalisation	Type of Hospital[1]	Have you undergone any surgery/major procedure?[2]	Type of Treatment Medical/ Surgical[3]	Duration of stay	Total amount spent on treatment (hospitalisation expenses+ medicine purchased+ transport cost to hospital during stay)	Source for payment[4]	If reimbursed from the insurance company, specify the amount (In Rupees)?

[1]**Type of Hospital**
A.Government
B.Private
C.Charity
D. Other (SPECIFY)___

[2] **Surgery/Major Procedure:**
A. Revascularisation/ bypass
B. Valve repair/ replacement
C. Stent
D. Pacemaker
E. Amputation
F. Renal Transplantation
G. Heart Transplantation
H. Retinal Photocoagulation
I. Other (SPECIFY)________

[3] **Type of Treatment:**
A. Medicines
B. Thyombolysis
C. Angiogram
D. Angioplasy
E. Bypass surgery
F. Brachytherapy
G. Pacemaker
H. Heart transplant
I. Amuputation
J. Echo cardiography
K. Neuro imaging
L. Dialysis
M. Kidney-transplant
N. For Observation
O. Other(Specify)______

[4] **Source of payment:**
A. Own saving
B. Family member paid
C. Employer paid
D. Borrowed from friend, relative & employer
E. Borrowed from bank
F. Sold house, land or other assests
G. Health Insurance
H. Cashless hospitalisation
I. Other (Specify)

End the Interview with Thanks

CONSENT FOR ANTHROPOMETRY, BLOOD SUGAR AND BLOOD PRESSURE MEASUREMENTS

As part of this survey, we are also studying level of blood sugar and blood pressure, and physical measurement such as height, weight and waist circumference, as these are the serious health problems related to lifestyle. The Health Worker from the health facility in your area will be doing these measurements. I will tell you the procedure of the measurement of blood sugar and blood pressure.

For blood pressure, a digital monitor will be used. For blood sugar, a sterile swap will be used to

For weight, you will be asked to stand on a weighing machine.

In order to determine your level of blood sugar, we are asking the survey participants to give a few drops of blood. The drops of blood will be collected from your finger. The report of all the above mentioned measurements will be given to you soon after its collection. In case you are screened with raised blood pressure and/or blood sugar, you will be referred to the nearest health facility for further investigation.

Participation in this effort is voluntary and you can choose not to participate. As informed you earlier, the participants name and physical identity will not be disclosed to anyone. This information will only be used for research and planning purpose.

At this time, do you have any questions to ask me?

ANSWER ANY QUESTIONS AND ADDRESS RESPONDENT’S CONCERNS.

IF RESPONDENT AGE IS 15-17 YEARS, READ THE ABOVE PARAGRAPH TO BOTH RESPONDENT AND GUARDIAN AND TAKE CONSENT FROM BOTH.

I hope you will agree to participate in this effort and give few drops of blood.

BOTH RESPONDENT AND GUARDIAN AGREED → 1 ⟶ CONTINUE MEASUREMENT

EITHER OF THEM DID NOT AGREE → 2 ⟶ EXIT INTERVIEW

I would request you to give your consent.

Medtronic Foundation India NCD Program

Situational analysis of Non Communicable Diseases

Date of Measurement: dd___mm____yy____

Respondent's ID Number: ....

Full Name of the Respondent:...

S.No.	Questions	Response	SKIP TO
1	Height	In Centimeters.......................	
2	Weight	In Kilograms..........................	
3	Body Mass Index (BMI) (Need not be calculated in the field)	....Kg/m2	
4	(For Women) Are you pregnant?	1. Yes→<br>2. No	→7
**Waist Measurement**			
5	Waist circumference Reading 1	In Centimeters.......................	
6	Waist circumference Reading 2	In Centimeters.......................	
**Blood Pressure**			
7	B.P. Reading 1	Systolic (mmHg).........................<br>Diastolic (mmHg)........................	
8	B.P. Reading 2	Systolic (mmHg).........................<br>Diastolic (mmHg)........................	
**Blood Sugar Measurement**			
9	Blood Sugar	Fasting..<br>PP...<br>Random..	

Consent of the respondent

Name & Signature

Technician / Nurse/ ANM

<table>
<tr><td colspan="6">MEDTRONIC FOUNDATION SUPPORTED INDIA NCD PROGRAM</td></tr>
<tr><td colspan="6"><u>Referral Card</u>
Date of Referral
(dd/mm/yyyy):</td></tr>
<tr><td colspan="6">Name and Address of the Respondent:</td></tr>
<tr><td colspan="6">Respondent’s ID Number:</td></tr>
<tr><td>Name:</td><td colspan="5">(First Name) (Middle Name) (Last Name)</td></tr>
<tr><td>Age:
(in years)</td><td></td><td colspan="2">Sex: (please encircle)</td><td>Female =1</td><td>Male =2</td></tr>
<tr><td colspan="3">Blood Pressure Reading (mmHg):
Systolic.......... Diastolic............</td><td colspan="3">Blood Sugar (mg/dl):
Fasting.....................PP...................Random.............</td></tr>
<tr><td colspan="6">Any symptoms/Signs:
………
………</td></tr>
<tr><td colspan="6">Name and Address of the Health Facility, referred to:
………
Block…………………………..District………..………………….State……………………………..</td></tr>
<tr><td colspan="6">(Name & Signature)</td></tr>
</table>
