# Supplementary material for: Identifying gaps in the continuum of care for hypertension and diabetes in two Indian communities
Source: BMC Health Serv Res. 2017 Dec 27;17:846. doi: 10.1186/s12913-017-2796-9 (PMC5746011; doi:10.1186/s12913-017-2796-9)
Supplement: Supplementary file 2 — Health facility survey. (PDF 2323 kb) [file 12913_2017_2796_MOESM2_ESM.pdf]

Survey Specifications
HealthRise Facility Survey

ENGLISH/HINDI VERSION

Platform Legend
PHC- Primary Health Center
SHC- Sub Health Centre
CHC – Community Health Center
Hosp- Hospital
ALL – All Health Facility Platforms

Table of Contents

Module 1: Inputs, Finances and Management 1
Section 1: Introduction, Consent and General Information 2
Section 2: Facility Inputs 6
Section 3: Facility Finances 9
Section 4: Facility Revenues 18
Section 5: Facility Staff Characteristics 22
Section 6: Facility Management 28
Module 2: Direct Observation and Facility Capacity 34
Section 1: Laboratory Consumables, Equipment, and Capacity 35
Section 2: Pharmaceuticals 37
Section 3: Medical Consumeables, Equipment, and Capacity 39
Section 4: Facility Characteristics 45
Module 3: Outputs 49
Section 1: General Outpatient Questions 51
Section 2: General Inpatient Questions 52
Section 2: Cardiovascular and Diabetes 54
Section 3: Emergency Care 58
Section 4: Laboratory and Diagnostic Tests 59

Unique Variable Name(s)	Source (Year & Item # if Known)		Item Conditions	Platform	State-Specific Modifications
		**Module 1: Inputs, Finances and Management**			
		**Survey Log-In**			
FAC_DISTRICT		Enter District ID: जिले की आईडी लिखें			
CUSTOMID	IHME	Enter the CUSTOMID for this facility:________ इस केन्द्र का कस्टमआईडी प्रविष्ट करें:________	required;	ALL	none
FIELDSTAFF_ID	IHME	Enter your Interviewer ID: ______________ अपना साक्षात्कारकर्ताआईडी प्रविष्ट करें: ______________	required; text only; page break	ALL	none

Unique Variable Name(s)	Source (Year & Item # if Known)		Item Conditions	Platform	State-Specific Modifications
AUTOID		AutoID			
		**Section 1: Introduction, Consent and General Information परिचय, स्वीकृति एवं सामान्य सूचनाएँ**			
		This section is designed to provide a brief introduction to the survey and to solicit consent from the facility administrator. Please complete this section with a facility administrator or manager.<br>इस अनुभाग को खासतौर पर सर्वे के संक्षिप्त परिचय और केन्द्र के प्रशासक की सहमति प्राप्त करने के लिए तैयार किया गया है। कृपया इस सेक्शन को केन्द्र के प्रशासक या प्रबन्धक की मदद से पूरा करें।			
		**Introduction**<br><br>Instructions for interviewer:<br>All text highlighted in yellow are directions for you and should not be read aloud<br>All text highlighted in green are observations only<br>Never read the options Don't know नहीं जानते , Decline to respond जवाब देने के लिए इंकार, or Not applicable नहीं लागू ; only fill those in when indicated by the respondent.			
TIME_START		Survey Start Time. Please click 'Get Time' button<br>सर्वे शुरू करने का समय। कृपया 'गेट टाइम' बटन पर क्लिक करें<br>[Get Time]____________________			
REPLACEMENT		Is this a replacement facility?<br>क्या यह रिप्लेसमेंट केन्द्र है?<br>(SELECT ONE)<br>☐ 1=Yes हां<br>☐ 0=No नहीं			
REPLACEMENT_NAME		Enter the name of this replacement facility__________<br>इस प्रतिस्थापन केन्द्र का नाम प्रविष्ट करें__________	current.replacement = 1		
REPLACEMENT_ADDRESS		Enter the physical address of this facility_______________<br>इस केन्द्र का भौतिक पता प्रविष्ट करें ______________	current.replacement = 1		
GPS_DEGREES<br>GPS_MIN<br>GPS_SEC		**Facility GPS Coordinates:**<br>केन्द्र के जीपीएस कोआर्डिनेट<br>Pleases enter in the same format as displayed on your GPS Device. कृपया जीपीएस उपकरण पर दर्शाये गये फार्मेट को उसी तरह से भरें।<br>Enter the latitude and longitude लैटीट्यूट और लांगीट्यूड (अक्षांस और देशान्तर) भरें<br><br>Enter the following codes, if necessary: आवश्यक होने पर ये कोड लिखें<br>-1= Error with GPS Device जीपीएस उपकरण में खराबी			
		(blank) / Enter degrees कोण लिखें / Enter minutes मिनट लिखें / Enter seconds सेकेण्ड लिखें			
		Latitude लैटीट्यूट / / /			
		Longitude लांगीट्यूड / / /			
FAC_ELEV		**Altitude in meters:** ______________________________<br>मीटर में ऊंचाई			
		**Consent**			

Unique Variable Name(s)	Source (Year & Item # if Known)		Item Conditions	Platform	State-Specific Modifications
		Is this facility in Shimla or Udaipur? क्या यह केन्द्र शिमला में है या उदयपुर में?<br>☐ 1=Shimla शिमला<br>☐ 2= Udaipur उदयपुर			
		Shimla Consent<br>शिमला के लिए सहमति<br>Read consent aloud to facility administrator<br>केन्द्र के प्रशासक के लिए सहमति तेज आवाज में पढें<br>We are conducting this survey on behalf of IGMC-Shimla and the University of Washington to better understand the burden of cardiovascular disease and diabetes in Shimla.<br>हम लोग आईजीएमसी-शिमला और यूनिवर्सिटी आफ वाशिंगटन की ओर से शिमला में हृदय रोग और डाइबिटीज के बोझ को समझने के लिए यह सर्वे कर रहे हैं।<br><br>Your facility was randomly selected to participate in this study. If you participate, we will ask you questions about facility inputs, activities related to cardiovascular disease and diabetes, and administration.<br>आपके केन्द्र को इस सर्वे में भाग लेने के लिए चुना गया है। यदि आप भाग लेते हैं तो हम आपसे हृदय रोग और डाइबिटीज के बारे में केन्द्र के निवेश औरर प्रशासन से संबंधित सवाल पूछेंगे।<br><br>Information about your facility will be compiled in a dataset that may be made publicly available and provided to researchers for analyses. However, neither your name nor that of any other respondents participating in this study will be included in the dataset or in any report. While the name of your facility will be collected, it will not be included in the publicly-available datasets.<br>आपके केन्द्र की जानकारी को इसी तरह की अन्य जानकारी के साथ मिलाकर सार्वजनिक रूप से उपलब्ध कराया जायेगा और विश्लेषण के लिए शोधकों को दिया जायेगा। लेकिन न तो आपके नाम को और न ही दूसरे उत्तरदाताओं के नाम को जानकारी के समूह में शामिल किया जायेगा और न ही किसी रिपोर्ट में। हालांकि आपके केन्द्र का नाम लिया जायेगा लेकिन इसे सार्वजनिक रूप से उपलब्ध कराये जाने वाले डाटाबेस में शामिल नहीं किया जायेगा।<br><br>As the facility administrator, a research assistant will ask you to find the required documents that pertain to records of activities and finances at the facility; this information will then be recorded electronically using the facility survey.<br>केन्द्र के प्रशासक के रूप में, कोई शोध सहायक आपसे केन्द्र की गतिविधियों और वित्त से संबंधित आवश्यक कागजात देने के लिए कहेगा। फिर इस जानकारी को केन्द्र के सर्वे में इलेक्ट्रानिक रूप से रिकार्ड किया जायेगा।<br><br>The data collected may be used by the Ministry of Health or other supporting organizations for planning service improvement and investment at your facility. Data may also be used for further studies of healthcare costs.<br>एकत्र की गयी जानकारी को स्वास्थ्य मंत्रालय या अन्य सहयोगी संस्थाओं द्वारा आपके केन्द्र की सेवाओं में सुधार और निवेश के लिए इस्तेमाल किया जायेगा। स्वास्थ्य पर होने वाले खर्चों के आगे के सर्वे के लिए भी जानकारी को इस्तेमाल किया जा सकता है।<br><br>We are asking for your help to ensure that the information we collect is accurate. If there are questions for which someone else is the most appropriate person to provide the information, please ask that person to assist you in the completion of this survey.<br>हम यह सुनिश्चित करने के लिए आपका सहयोग चाहते हैं कि एकत्र की गयी जानकारी बिलकुल सही-सही हो। यदि ऐसा कोई सवाल है जिसके लिए कोई दूसरा व्यक्ति सबसे उपयुक्त व्यक्ति है जो जानकारी दे सकता है तो कृपया उस व्यक्ति को इस सर्वे को पूरा करने में सहयोग करने के लिए कहें।			

Unique Variable Name(s)	Source (Year & Item # if Known)		Item Conditions	Platform	State-Specific Modifications
		Udaipur Consent<br>उदयपुर के लिए सहमति<br>Read consent aloud to facility administrator<br>केन्द्र के प्रशासक के लिए सहमति तेज आवाज में पढ़ें<br>We are conducting this survey on behalf of GfK Mode and the University of Washington to better understand the burden of cardiovascular disease and diabetes in Udaipur.<br>हम लोग जीएफके मोड और यूनिवर्सिटी आफ वाशिंगटन की ओर से उदयपुर में हृदय रोग और डाइबिटीज के बोझ को समझने के लिए यह सर्वे कर रहे हैं।<br><br>Your facility was randomly selected to participate in this study. If you participate, we will ask you questions about facility inputs, activities related to cardiovascular disease and diabetes, and administration.<br>हम लोग आईजीएमसी-शिमला और यूनिवर्सिटी आफ वाशिंगटन की ओर से शिमला में हृदय रोगों और डाइबिटीज के बोझ को समझने के लिए यह सर्वे कर रहे हैं।<br><br>Information about your facility will be compiled in a dataset that may be made publicly available and provided to researchers for analyses. However, neither your name nor that of any other respondents participating in this study will be included in the dataset or in any report. While the name of your facility will be collected, it will not be included in the publicly-available datasets.<br>आपके केन्द्र की जानकारी को इसी तरह की अन्य जानकारी के साथ मिलाकर सार्वजनिक रूप से उपलब्ध कराया जायेगा और विश्लेषण के लिए शोधकों को दिया जायेगा। लेकिन न तो आपके नाम को और न ही दूसरे उत्तरदाताओं के नाम को जानकारी के समूह में शामिल किया जायेगा और न ही किसी रिपोर्ट में। हालांकि आपके केन्द्र का नाम लिया जायेगा लेकिन इसे सार्वजनिक रूप से उपलब्ध कराये जाने वाले डाटाबेस में शामिल नहीं किया जायेगा।<br><br>As the facility administrator, a research assistant will ask you to find the required documents that pertain to records of activities and finances at the facility; this information will then be recorded electronically using the facility survey.<br>केन्द्र के प्रशासक के रूप में, कोई शोध सहायक आपसे केन्द्र की गतिविधियों और वित्त से संबंधित आवश्यक कागजात देने के लिए कहेगा। फिर इस जानकारी को केन्द्र के सर्वे में इलेक्ट्रानिक रूप से रिकार्ड किया जायेगा।<br><br>The data collected may be used by the Ministry of Health or other supporting organizations for planning service improvement and investment at your facility. Data may also be used for further studies of healthcare costs.<br>एकत्र की गयी जानकारी को स्वास्थ्य मंत्रालय या अन्य सहयोगी संस्थाओं द्वारा आपके केन्द्र की सेवाओं में सुधार और निवेश के लिए इस्तेमाल किया जायेगा। स्वास्थ्य पर होने वाले खर्चों के आगे के सर्वे के लिए भी जानकारी को इस्तेमाल किया जा सकता है।<br><br>We are asking for your help to ensure that the information we collect is accurate. If there are questions for which someone else is the most appropriate person to provide the information, please ask that person to assist you in the completion of this survey.<br>हम यह सुनिश्चित करने के लिए आपका सहयोग चाहते हैं कि एकत्र की गयी जानकारी बिलकुल सही-सही हो। यदि ऐसा कोई सवाल है जिसके लिए कोई दूसरा व्यक्ति सबसे उपयुक्त व्यक्ति है जो जानकारी दे सकता है तो कृपया उस व्यक्ति को इस सर्वे को पूरा करने में सहयोग करने के लिए कहें।			

Unique Variable Name(s)	Source (Year & Item # if Known)		Item Conditions	Platform	State-Specific Modifications
CONSENT	IHME<br>DHS SPA	Read consent aloud to facility administrator<br>केन्द्र प्रशासक को सहमति पत्र जोर से पढ़ कर सुनायें।<br><br>Do you agree to proceed?<br>क्या आप आगे बढ़ने के लिए सहमत हैं?<br>(SELECT ONE)<br>☐ 1=Yes हां<br>☐ 0=No नहीं		ALL	none
COMMENTS_SECTION1	IHME	Is there an error in our information regarding this facility?<br>क्या इस केन्द्र के संबंध में हमारी जानकारी में कोई अशुद्धि है?<br>☐ 1=Yes हां<br>☐ 0=No नहीं		ALL	none
		Please use this space to provide the correct information. कृपया इस जगह को सही जानकारी देने के लिए इस्तेमाल करें।<br>It is essential that we have the correct information on file for all participating facilities<br>यह जरूरी है कि केन्द्र के सभी प्रतिभागियों के लिए फाइल पर सही जानकारी हो<br>(OPEN TEXT)			
PLATFORM_TYPE	IHME	Health service delivery platform type:<br>स्वास्थ्य सेवा प्रदान करने वाले प्लेटफॉर्म के प्रकार:<br>(SELECT ONE)<br>☐ 1= District Hospital (DH) जिला अस्पताल (डीएच)<br>☐ 2 = Civil Hospital (CH) सिविल अस्पताल (सीएच)<br>☐ 3 = Community Health Centre (CHC) सामुदायिक स्वास्थ्य केन्द्र (सीएचसी)<br>☐ 4 = Primary Health Centre (PHC) प्राथमिक स्वास्थ्य केन्द्र (पीएचसी)<br>☐ 5 = Sub Health Centre (SHC) उपस्वास्थ्य केन्द्र (एसएचसी)<br>☐ 6 = Private Hospital प्राइवेट अस्पताल<br>☐ 7= Private Clinic प्राइवेट क्लीनिक	current.consent = 1	ALL	Country specific platform types (with follow-up questions to define facility type as needed)
FAC_PRIVATE	IHME	Is this a private facility?<br>क्या यह प्राइवेट स्वास्थ्य केन्द्र है?<br><br>☐ 1=Yes हां<br>☐ 0=No नहीं	(current.platform_type = 7)<br>OR<br>(currnet.platform_type = 8)	ALL	none
COMMENTSM1_1		Comments for Section 1: Consent ______________________<br>सेक्शन 1 के लिए टिप्पणी<br><br>(OPEN TEXT)			

Unique Variable Name(s)	Source (Year & Item # if Known)		Item Conditions	Platform	State-Specific Modifications
		**Section 2: Facility Inputs**			
SECTION_2_TITLE	IHME	This section is primarily designed to capture information about this facility's inputs. Please complete this section with a facility administrator, manager or other individual who is well-informed about the inputs to this health facility.<br>इस अनुभाग को खासतौर पर इस केंद्र के इनपुट की जानकारी लेने के लिए बनाया गया है कृपया इस अनुभाग को केंद्र संचालक, प्रबंधक या किसी अन्य व्यक्ति जो कि इस केंद्र के इनपुट के बारे में अच्छी तरह जानता हो, उसके सहयोग से भरें			
FAC_YEARS	IHME	How many years in total has this facility been in existence?<br>यह केन्द्र कुल कितने वर्षों से अस्तित्व में है?<br>(If the administrator is unaware, please enter their best estimate.) यदि व्यवस्थापक को पता नहीं है तो कृपया उनके सर्वश्रेष्ठ अनुमान को भरें<br>☐ 1=ENTER NUMBER OF YEARS: ______<br>वर्षों की संख्या लिखें		ALL	none
FAC_YEARS2	IHME	How many years has this facility been a [PLATFORM_TYPE]<br>यह केन्द्र कितने वर्षों से एक [PLATFORM_TYPE] है<br>☐ 1=ENTER NUMBER OF YEARS: ______<br>वर्षों की संख्या लिखें		ALL	none
		**National Programme for Prevention and Control of Cancer, Diabetes, Cardiovascular Diseases, & Stroke (NPCDCS)**<br>कैंसर, डाइबिटीज, ह्रदय रोग, आघात के रोकथाम और नियंत्रण का राष्ट्रीय कार्यक्रम (एनपीसीडीसीएस)			Specific to States that have implemented NPCDCS
		Have you ever heard of the National Programme for Prevention and Control of Cancer, Diabetes, Cardiovascular Diseases, & Stroke (NPCDCS)?<br>क्या आपने कभी कैंसर, डाइबिटीज, ह्रदय रोग, आघात के रोकथाम और नियंत्रण के राष्ट्रीय कार्यक्रम (एनपीसीडीसीएस) के बारे में सुना है?<br>☐ 1=Yes हां<br>☐ 0=No नहीं<br>☐ -1=Don't know नहीं जानते<br>☐ -2=Decline to respond जवाब देने से इंकार		ALL	
		Has this facility ever made changes or implemented programs in response to the National Programme for Prevention and Control of Cancer, Diabetes, Cardiovascular Diseases, & Stroke (NPCDCS)?<br>क्या इस केन्द्र ने कभी कैंसर, डाइबिटीज, ह्रदय रोग, आघात के रोकथाम और नियंत्रण के राष्ट्रीय कार्यक्रम (एनपीसीडीसीएस) के प्रत्युत्तर में कार्यक्रमों में बदलाव या सुधार किये हैं?<br>☐ 1=Yes हां<br>☐ 0=No नहीं<br>☐ -1=Don't know नहीं जानते<br>☐ -2=Decline to respond जवाब देने से इंकार		ALL	

Unique Variable Name(s)	Source (Year & Item # if Known)		Item Conditions	Platform	State-Specific Modifications
		Please Specify what this facility has done in response to the National Programme for Prevention and Control of Cancer, Diabetes, Cardiovascular Diseases, & Stroke (NPCDCS):___________<br>कृपया स्पष्ट करें कि कैंसर, डाइबिटीज, हृदय रोग, आघात के रोकथाम और नियंत्रण के राष्ट्रीय कार्यक्रम (एनपीसीडीसीएस) के प्रत्युत्तर में केन्द्र क्या ने किया है?<br><br>Enter the following codes, if necessary: आवश्यक होने पर ये कोड लिखें<br>1=Don't know नहीं जानते<br>-2=Decline to respond जवाब देने से इंकार			
		Has this facility received funding from the National Programme for Prevention and Control of Cancer, Diabetes, Cardiovascular Diseases, & Stroke (NPCDCS)?<br>क्या इस केन्द्र ने कैंसर, डाइबिटीज, हृदय रोग, आघात के रोकथाम और नियंत्रण के राष्ट्रीय कार्यक्रम (एनपीसीडीसीएस) से कोई आर्थिक सहायता प्राप्त की है?<br>☐ 1=Yes हां<br>☐ 0=No नहीं<br>☐ -1=Don't know नहीं जानते<br>☐ -2=Decline to respond जवाब देने से इंकार		ALL	
		How much funding have you received from the National Programme for Prevention and Control of Cancer, Diabetes, Cardiovascular Diseases, & Stroke (NPCDCS) in the last year?<br>आपने पिछले वर्ष कैंसर, डाइबिटीज, हृदय रोग, आघात के रोकथाम और नियंत्रण के राष्ट्रीय कार्यक्रम (एनपीसीडीसीएस) से कितनी आर्थिक सहायता प्राप्त की है?<br>(Numeric) ___________________________<br><br>Enter the following codes, if necessary: आवश्यक होने पर ये कोड लिखें<br>1=Don't know नहीं जानते<br>-2=Decline to respond जवाब देने से इंकार		ALL	
PHARM_RECORDS_WHO_T		Who is responsible for the records of indents/orders?<br>इन्डेन्ट/आर्डर के रिकार्ड के लिए कौन व्यक्ति जिम्मेदार है?<br>(SELECT ALL THAT APPLY) (एक से अधिक उत्तर सम्भव)<br>☐ 2= Pharmacist फार्मासिस्ट<br>☐ 1=Facility Accountant केन्द्र का एकाउंटेन्ट<br>☐ Matron मैट्रन<br>☐ Ward Nurse वार्ड नर्स<br>☐ Ward Sister वार्ड सिस्टर<br>☐ 0=Other , please specify अन्य, कृपया स्पष्ट करें<br>☐ -1=Don't know नहीं जानते<br>☐ -2=Decline to respond जवाब देने के लिए इंकार		ALL	none
PHARM_RECORDS_WHO_OSP		Please specify 'other': _______________<br>कृपया 'अन्य' को बतायें			

Unique Variable Name(s)	Source (Year & Item # if Known)					Item Conditions	Platform	State-Specific Modifications
PHARM_RECORDS_PAYMENTS_WHO_T		Who in this facility is responsible for the records of estimated costs of the indents/orders for pharmaceuticals?<br>दवाओं के लिए इन्डेन्ट/आर्डर की अनुमानित लागत के रिकार्ड के लिए इस केन्द्र पर कौन व्यक्ति जिम्मेदार है?<br>(SELECT ALL THAT APPLY) (एक से अधिक उत्तर सम्भव)<br>☐ 2= Pharmacist फार्मासिस्ट<br>☐ 1=Facility Accountant केन्द्र का एकाउंटेन्ट<br>☐ Matron मैट्रन<br>☐ Ward Nurse वार्ड नर्स<br>☐ Ward Sister वार्ड सिस्टर<br>☐ 3=Not kept at facility केन्द्र पर नहीं रखा जाता<br>☐ 0=Other, please specify अन्य, कृपया स्पष्ट करें<br>☐ -1=Don't know नहीं जानते<br>☐ -2=Decline to respond जवाब देने के लिए इंकार					ALL	none
PHARM_RECORDS_PAYMENTS_WHO_OSP		Please specify 'other': _______________<br>कृपया 'अन्य' को बतायें						
INPUT_IU_ITEMS	IHME<br>CSTS<br>DHS SPA<br>Rand<br>WHO SAM	*Display as a table:*<br>Please indicate how many of the following items are available at this facility **TODAY**, and of that total, how many of the following items are functional at this facility today.<br>कृपया बतायें कि **आज** इस केन्द्र पर निम्नलिखित में से कितने सामान उपलब्ध हैं, और उनमें से आज इस केन्द्र पर निम्नलिखित में से कितने सामान काम कर रहे हैं.<br>Enter the following codes, if necessary: आवश्यक होने पर ये कोड लिखें<br>0=None कोई नहीं<br>-1=Don't know नहीं जानते<br>-2=Decline to respond जवाब देने के लिए इंकार				required; set default values to 0	ALL	
		**Item मद**	**Total number available कुल उपलब्ध संख्या**	**Total number owned by facility केन्द्र के पास कुल सामानों की संख्या**	**Total number functional कुल क्रियाशील संख्या**			
IU_ITEMS_AVAIL<br>IU_ITEMS_FUNC	"	Back-up or standby generator or inverter for electricity<br>बिजली के लिए बैकअप या उसकी जगह पर जनरेटर या इन्वर्टर					ALL	none
	"	Landline telephone<br>लैण्डलाइन टेलीफोन					ALL	none
	"	Cellular telephone<br>सेलफोन					ALL	none
	"	Computer<br>कंप्यूटर					ALL	none

Unique Variable Name(s)	Source (Year & Item # if Known)		Item Conditions	Platform	State-Specific Modifications
IU_VEHICLES		Does this facility have any vehicles (this includes 4 and 2 wheeled motorized vehicles)? Please include all vehicles that are used at this facility, including those that may be rented.<br>क्या इस केन्द्र पर कोई वाहन हैं (इसमें चार पहिया और 2 पहिया वाले मोटर वाहन शामिल हैं)? कृपया उन सभी वाहनों को शामिल करें जो इस केन्द्र पर हैं और उनको भी शामिल करें जो किराये पर हों।<br>☐ 1=Yes हां<br>☐ 0=No नहीं<br>☐ -1=Don't know नहीं जानते<br>☐ -2=Decline to respond जवाब देने के लिए इंकार		ALL	none
COMMENTSM1_2		Comments for Section 2: Inputs ___________<br>सेक्शन दो के लिए टिप्पणी : जानकारी			
		**Section 3: Facility Finances**			
		This section is primarily designed to capture information about this facility's finances. Please complete this section with a facility accountant, administrator or other individual who is well-informed about the finances to this health facility.<br>इस अनुभाग को खासतौर पर इस केंद्र के वितीय जानकारी लेने के लिए बनाया गया है कृपया इस अनुभाग को केंद्र के लेखाकार, संचालक या किसी अन्य व्यक्ति जो कि इस केंद्र के वित सम्बंधित जानकारी से अवगत हो, के सहयोग से भरें.			
SAL_REC	IHME	Does this facility keep records of the salaries/wages/benefits paid to staff at this facility?<br>क्या यह केन्द्र कर्मचारियों को मिलने वाले मजदूरी / वेतन/ लाभ का रिकार्ड रखता है?<br>☐ 1=Yes हां<br>☐ 0=No नहीं<br>☐ -1=Don't know नहीं जानते<br>☐ -2=Decline to respond जवाब देने से इंकार	Page break	ALL	
SAL_REC_NO_T	IHME	Where are records of the salaries/wages/benefits paid to staff at this facility kept?<br>इस केन्द्र में कर्मचारियों को मिलने वाले मजदूरी / वेतन/लाभ का रिकार्ड कहाँ रखा जाता है?<br>(SELECT ALL THAT APPLY) (एक से अधिक उत्तर सम्भव)<br>☐ 5= Block CHC ब्लाक सीएचसी<br>☐ 4=CDMO office सीडीएमओ आफिस<br>☐ 0=Other (specify) अन्य, कृपया स्पष्ट करें<br>☐ -1=Don't know नहीं जानते<br>☐ -2=Decline to respond जवाब देने के लिए इंकार	Current.sal_rec	ALL	
SAL_REC_NO_OSP		Please specify 'other': _________________<br>कृपया 'अन्य' को बतायें			
FISCAL_YR		Are the start and end dates of this facility's most recent fiscal year April 2013 - March 2014?<br>क्या इस केन्द्र के शुरुआत और अंत की तारीखें वित्तीय वर्ष अप्रैल 2013 - मार्च 2014 में बिलकुल हाल ही की हैं?<br>☐ 1=Yes हां<br>☐ 0=No नहीं			

Unique Variable Name(s)	Source (Year & Item # if Known)		Item Conditions	Platform	State-Specific Modifications
FISCAL_YR2_MONTH<br>FISCAL_YR2_YEAR		What is the first month of the most recently completed fiscal year?<br>बिलकुल हाल ही में पूरा किये गये वित्त वर्ष का पहला महीना कौन सा है?<br>Month: महीना:________________(January, February, March, April, May, June, July, August, September, October, November, December)<br>(जनवरी, फरवरी, मार्च, अप्रैल, मई, जून, जुलाई, अगस्त, सितम्बर, अक्तूबर, नवम्बर, दिसम्बर)<br>Year: वर्ष:________________(2012, 2013)	current.fiscal_yr = 0		
ATQ_ACC_T		Please indicate if this facility utilizes any of the following accounting systems:<br>कृपया सूचित करें कि क्या यह केंद्र निम्नलिखित लेखा प्रणाली में से किसी एक भी इस्तेमाल करता है:<br>(SELECT ALL THAT APPLY) (एक से अधिक उत्तर सम्भव)<br>☐ 2=Paper-based accounting system कागजी लेखा पद्धति<br>☐ 1=Electronic-based accounting system इलेक्ट्रानिक लेखा पद्धति<br>☐ 0=None of the above इनमें से कोई नहीं<br>☐ -1=Don't know नहीं जानते<br>☐ -2=Decline to respond जवाब देने से इंकार		ALL	None
exp_month_year		Are facility expenses recorded by month or by year?<br>क्या केन्द्र के खर्चे महीने के अनुसार या साल के अनुसार रिकार्ड किये जाते हैं?<br>☐ 1=By Month महीने के अनुसार<br>☐ 2=By Year साल के अनुसार			
INSTRUCT_EXP_AND_FISCAL_YRS	IHME	**Facility Expenses**<br>केन्द्र के खर्चे<br>**Complete the following Facility Expenses extraction for All facilites by clicking the below button.**<br>कृपया नीचे का बटन दबाकर सभी केन्द्रों के इन केन्द्र खर्च को पूरा करें<br>In this section, we are asking about the total amount of money this facility spent during the previous fiscal year. We are attempting to capture the total amount spent on certain categories of expenditures.<br>इस सेक्शन में हम यह पूछ रहे हैं कि इस केन्द्र ने पिछले वित्त वर्ष में कुल कितना धन खर्च किया। हम खर्च की कुछ कैटेगरी पर किये गये कुल खर्च को पता करना चाहते हैं।<br>**Ask to organize the following records:** निम्न रिकार्ड व्यवस्थित करने के बारे में पूछें<br>• Total Expenses कुल खर्च<br>• Infrastructure andUtilities ढांचागत एवं युटिलिटीज<br>• Medical Suppliesnd Equipment मेडिकल सप्लाई और उपकरण<br>• Pharmaceuticals फार्मास्युटिकल्स<br>• Facility Administration and Staff Training केन्द्र का प्रशासन एवं कर्मचारियों का प्रशिक्षण<br>• Non-Medical Services गैर चिकित्सा सेवाएं<br>• Staff (Salaries and Benefits) कर्मचारी (वेतन और लाभ)<br><br>While this section will primarily cover expenditures, in certain cases we have paired expenditure questions with questions covering the input and output associated with these expenses which we hope will facilitate a review of this facility's records.<br>हालांकि इस भाग में मुख्य रुप से खर्च के बारे में पूछा जायेगा लेकिन कुछ मामलों में इन खर्चों के निवेश और लाभ से संबंधित प्रश्न भी पूछे जायेंगे जो इस केन्द्र के रिकार्ड की समीक्षा करने में मदद करेंगे	use calculation to control number of years shown (based on response:FAC_YEARS_TEXT)	ALL	

Unique Variable Name(s)	Source (Year & Item # if Known)			Item Conditions	Platform	State-Specific Modifications
TAB_INSTRUCT_EXP_TOTAL<br><br>EXP_TOTAL	IHME	Please note, for all tables such as the one below, use the "Tab" key to move to the next cell space.<br>कृपया ध्यान दें कि नीचे की सभी तालिकाओं के लिए अगले सेल में जाने के लिए 'टैब' की का प्रयोग करें।<br><br>Report all money spent directly by this facility on the following categories of expenditures for the most recent fiscal year.<br>साल दर साल इस केन्द्र के द्वारा व्यय की निम्न श्रेणियों पर बिलकुल हाल ही में खर्च किए गए धन के बारे में बतायें.<br><br>Enter the following codes, if necessary: यदि आवश्यक हो तो निम्न कोड प्रविष्ट करें, लिखें<br>0=No expenses कोई खर्च नहीं<br>-1=Don't know नहीं जानते<br>-2=Decline to respond जवाब देने से इंकार<br><br>All amounts should be listed in Indian rupees<br>सभी धनराशि रुपये में दी जाय		required;column headings are populated by FY end dates range input into Collection "C_FINANCIAL_YRS";	ALL	Currency
		**Total expenses** कुल खर्च	Last Fiscal Year (Month 1, Month 2, Mont 3, Month 4, Mont 5, Month 6, Month 7, Month 8, Month 9, Month 10, Month 11, Month 12) or (Year 1)<br>पिछले वित्त वर्ष (महीना 1, महीना 2, महीना 3, महीना 4, महीना 5, महीना 6, महीना 7, महीना 8, महीना 9, महीना 10, महीना 11, महीना 12) या (एक साल)	"		
TE_	IHME	**TOTAL amount spent by the facility in the last fiscal year**<br>पिछले वित्त वर्ष में केन्द्र द्वारा खर्च की गयी कुल धनराशि		Ensure that sub-components are at least not more than 100% of reported total. Soft prompt if they are less than 50% of reported total.	ALL	Rajasthan
TAB_INSTRUCT_EXP_IU;<br>EXP_IU		**Infrastructure and utilities** **बुनियादी ढांचा और सुविधाएं**	Last Fiscal Year पिछला वित्त वर्ष	"	All	Rajasthan

Unique Variable Name(s)	Source (Year & Item # if Known)			Item Conditions	Platform	State-Specific Modifications
IU_	IHME	(1) Building and Infrastructure costs. बिल्डिंग और बुनियादी ढांचा लागत<br>Include and add up any of the folowing following expenses the facility had in the past year:<br>पिछले साल में इस केन्द्र द्वारा किये गये इनमें से किसी भी खर्च को शामिल करें और जोडें<br>a. Rent or Mortgage किराया या गिरवी<br>b. Repair or renovation projects मरम्मत या नवीकरण परियोजनायें<br>c. Other Building and Infrastructure Costs such as new windows and other expenses related to the building itself अन्य भवन और ढांचागत खर्च जैसे नयी खिडकी और अन्य भवन से संबंधित खर्च				
	IHME	(2) Capital and maintenance costs (non-medical equipment and assets). Include acquisition costs and maintenance costs. पूँजी और रखरखाव लागत (गैर चिकित्सा उपकरण और संपत्ति). अधिग्रहण खर्च और रखरखाव खर्च को शामिल करें<br>Include and add up any of the folowing following expenses the facility had in the past year:<br>पिछले साल में इस केन्द्र द्वारा किये गये इनमें से किसी भी खर्च को शामिल करें और जोडें<br>a. Transport vehicles. Include acquisition costs and maintenance costs.<br>यातायात वाहन। अधिग्रहण और रखरखाव खर्च को शामिल करें<br>b. Generators जनरेटर<br>c. Landline or cellular telephones लैण्डलाइन और सेलुलर टेलीफोन<br>d. Computers कंप्यूटर<br>e. Refrigerators फ्रिज<br>f. Security systems सुरक्षा प्रणाली<br>g. Textbooks and teaching supplies. टेक्स्टबुक एवं शिक्षण सामग्री<br>h. Any other non-medical equipment costs such as chairs, tables, water storage tank, cash register or others<br>अन्य कोई गैर चिकित्सा उपकरण खर्च जैसे कुर्सी, मेज, पानी एकत्र करने का टैंक, कैश रजिस्ट या अन्य				

Unique Variable Name(s)	Source (Year & Item # if Known)			Item Conditions	Platform	State-Specific Modifications
	IHME	(3) Utilities.<br>युटिलिटीज<br><br>Include and add up any of the folowing following expenses the facility had in the past year:<br>पिछले साल में इस केन्द्र द्वारा किये गये इनमें से किसी भी खर्च को शामिल करें और जोडें<br>a. Water utilities जल युटिलिटि<br>b. Fuel for generator or electricity connection जनरेटर के लिए ईंधन या बिजली कनेक्शन<br>c. Internet connection and computer charges इंटरनेट कनेक्शन एवं कंप्यूटर के खर्चे<br>d. Temperature regulation systems for heating and cooling the facility केन्द्र को गरम और ठंडा रखने के लिए तापमान नियंत्रक प्रणाली<br>e. Mobile Medical Unit contingencies आकस्मिक स्थिति के लिए मोबाइल मेडिकल यूनिट<br>f. Waste Disposal कूडाकरकट निपटान<br>g. Other utility expenses केन्द्र के अन्य खर्च				
		(4) Depreciation ह्रास				
		(5) Bad Debt अशोध्य कर्ज				
		(6) Outreach activities<br>लोगों तक पहुंच के कार्य कलाप				
YEARS_REMODELED		Did the reported amount spent on building costs include a large or major renovation project during the previous fiscal year?<br>क्या बतायी गयी धनराशि को पिछले वित्त वर्ष के दौरान भवन खर्च पर इस्तेमाल किया जिसमें बडे और प्रमुख नवीकरण खर्च शामिल हैं?<br>☐ 1=Yes हां<br>☐ 0=No नहीं<br>☐ -1=Don't know नहीं जानते<br>☐ -2=Decline to respond जवाब से इंकार			ALL	Rajasthan
YEARS_VEHICLES		Did the reported amount spent on capital costs include the purchase of vehicles during any of the previous fiscal year?<br>क्या बतायी गयी धनराशि पूंजीगत लागत पर खर्च की गयी जिसमें पिछले वित्त वर्ष के दौरान खरीदे गये वाहन शामिल हैं?<br>☐ 1=Yes हां<br>☐ 0=No नहीं<br>☐ -1=Don't know नहीं जानते<br>☐ -2=Decline to respond जवाब से इंकार			ALL	Rajasthan

Unique Variable Name(s)	Source (Year & Item # if Known)			Item Conditions	Platform	State-Specific Modifications
TAB_INSTRUCT_EXP_MSE<br>EXP_MSE		**Medical Supplies and equipment चिकित्सा आपूर्ति और उपकरण**	Last Fiscal Year पिछला वित्त वर्ष	"	ALL	Rajasthan
MSE	IHME	(1)Surgical and general medical supplies (consumable goods). **Exclude pharmaceuticals.**<br>सर्जिकल एवं सामान्य चिकित्सा आपूर्ति (कंज्यूमेबल सामान) **फार्मास्युटिकल को छोडकर**<br>Include and add up any of the folowing following expenses the facility had in the past year:<br>पिछले साल में इस केन्द्र द्वारा किये गये इनमें से किसी भी खर्च को शामिल करें और जोडें<br>a. Syringes, bandages, gloves and/or suture kits<br>सिरिंज, बैण्डेज, दस्ताने और या चोट सिलाई किट<br>b. Laboratory reagent<br>प्रयोगशाला रीजेन्ट<br>c. Other consumable surgical and general medical supply related expenses<br>अन्य सर्जिकल कंज्यूमेबल एवं सामान्य चिकित्सा आपूर्ति संबंधी खर्चे				
	IHME	(2) Medical equipment (durable goods). Include acquisition and maintenance costs.<br>चिकित्सा उपकरण (ड्यूरेबल चीजें) अधिग्रहण और रखरखाव खर्च को शामिल करें<br>a. Beds बिस्तर<br>b. Medical machines and monitors चिकित्सा मशीने और मानीटर<br>c. Laboratory equipment (e.g. slides, test tubes etc.) प्रयोगशाला उपकरण (जैसे स्लाइड, टेस्ट ट्यूब आदि)<br>d. Other durable medical equipment expenses<br>अन्य ड्यूरेबल चिकित्सा उपकरण के खर्च				
TAB_INSTRUCT_EXP_PHRM<br>EXP_PHARM		**Pharmaceuticals फर्मास्युटिकल**	Last Fiscal Year पिछला वित्त वर्ष		ALL	Rajasthan
PHRM_	IHME	**TOTAL amount spent by the facility on pharmaceuticals for each year shown.**<br>दिखाये गये प्रति वर्ष में केन्द्र द्वारा फार्मास्युटिकल पर किया गया कुल खर्च		Populate from above		
		**Facility administration and staff training केन्द्र प्रशासन और कर्मचारियों का प्रशिक्षण**	Last Fiscal Year पिछला वित्त वर्ष		ALL	Rajasthan
ATQ_	IHME	(1) Amount spent on taxes (all taxes)<br>टैक्स पर खर्च की गयी राशि (सभी टैक्स)				

Unique Variable Name(s)	Source (Year & Item # if Known)			Item Conditions	Platform	State-Specific Modifications
	IHME	(2) Amount spent on interest on facility loans<br>केन्द्र द्वारा लिये गये कर्ज पर ब्याज के लिए खर्च की राशि				
	IHME	(3) Amount spent on insurance<br>बीमा पर खर्च की गयी राशि				
		(4) Amount spent on staff training (including staff per diems, instructor training fee)<br>कर्मचारियों के प्रशिक्ष्ाण पर खर्च की गयी राशि (कर्मचारियों के रोजाना के खर्च, प्रशिक्षक के प्रशिक्षण शुल्क को शामिल करते हुए)				
		(5) Amount spent on office expenses<br>आफिस के खर्चों पर खर्च की गयी राशि				
		(6) Amount spent on health record systems<br>स्वास्थ्य रिकार्ड सिस्टम पर खर्च की गयी राशि				
		(7) Amount spent on advertising and/or promotional activities.<br>विज्ञापन और/या प्रमोशन गतिविधियों पर खर्च की गयी राशि				
		(8) Other facility administration related expenses<br>केन्द्र के प्रशासन संबंधी अन्य खर्च				
TAB_INSTRUCT_EXP_NM<br>EXP_NONMEDSERVICES	IHME	**Non-medical services गैर चिकित्सा सेवायें**	Last Fiscal Year पिछला वित्त वर्ष		ALL	Rajasthan
NM		(1) Amount spent on patient food, staff food and catering (excluding salaries paid by facility to cantine staff)<br>मरीजों के भोजन, कर्मचारियों के भोजन और खानपान पर खर्च (केन्द्र द्वारा कैन्टीन कर्मचारियों के वेतन अदायगी को छोडकर)				
		(2) Amount spent on uniforms and laundry (excluding salaries paid by facility to laundry staff)<br>यूनीफार्म और कपडों की धुलाई पर खर्च की गयी राशि (लाण्ड्री कर्चारियों के वेतन पर केन्द्र द्वारा किये गये खर्च को छोडकर)				
		(3)Amount spent on housing for staff (excluding salaries paid by facility to housing maintenance or cleaning staff)<br>कर्मचारियों के हाउसिंग पर खर्च की गयी राशि (हाउसिंग रखरखाव या सफाई कर्मचारियों को केन्द्र द्वारा दिये गये वेतन को छोडकर)				
		(4) Amount spent on child care for staff's children (excluding salaries paid by facility directly to child care staff)<br>कर्मचारियों के बच्चों की देखरेख पर खर्च की गयी धनराशि (शिशु देखभाल कर्मचारियों को केन्द्र द्वारा की गयी सीधी वेतन अदायगी को छोडकर)				
		(5) Other non-medical service expenses (excluding salaries paid to staff)<br>अन्य गैर चिकित्सा सेवाओं के खर्च (कर्मचारियों को दिये गये वेतन को छोडकर)				

Unique Variable Name(s)	Source (Year & Item # if Known)			Item Conditions	Platform	State-Specific Modifications
		(7) Amount spent on security services (excluding salaries paid by facility to security staff)<br>सुरक्षा सेवाओं पर खर्च की गयी धनराशि (सुरक्षा कर्मचारियों को केन्द्र द्वारा दिये गये वेतन को छोडकर)				
TAB_INSTRUCT_EXP_PS1<br>EXP_PS1		*Display as a table:*<br><br>**Staff (1)- Total amount spent on all staff**<br>सभी कर्मचारियों पर खर्च की गयी कुल धनराशि<br><br>Please report the total expenditure that this facility has spent during the previous fiscal year. This includes all monetary resources used by this facility on staff (current and former) at this facility including wages, salaries, benefits and allowances over the course of a year, regardless of their source. This will include expenses for regular, ad-hoc, contractual, daily wages or part-time employees.<br>कृपया बतायें कि पिछले वित्त वर्ष के दौरान इस केन्द्र ने कुल कितना खर्च किया। उस कुल व्यय के बारे में बतायें. इसमें इस केन्द्र पर स्टाफ (वर्तमान और पूर्व) पर इस केन्द्र द्वारा प्रयोग किए गए सभी मौद्रिक संसाधन शामिल हैं जिसमें एक वर्ष के दौरान मजदूरी, वेतन, लाभ और भता, बिना उनके स्रोत की परवाह किए शामिल है. इसमें नियमित, तदर्थ, अनुबंध, दैनिक मजदूरी या अंशकालिक कर्मचारियों का खर्च शामिल होगा.				Rajasthan
			Last Fiscal Year पिछला वित्त वर्ष		ALL	Rajasthan
PS1		**TOTAL amount spent on all staff (current and former) at this facility including wages, salaries, benefits and allowances for the previous fiscal year**<br>पिछले वित्त वर्ष में इस केन्द्र के सभी कर्मचारियों (वर्तमान और पूर्व) पर खर्च की गयी कुल राशि जिसमें मजदूरी, वेतन, लाभ और भत्ता शामिल हैं				
TAB_INSTRUCT_EXP_PS1<br><br>EXP_SALARIES1	IHME	*Display as a table:*<br><br>**Staff (2) – Salaries/Wages**<br>कर्मचारी (2) वेतन/मजदूरी<br>For each category listed below, please report the total expenditures that this facility spent during the previous fiscal year. This includes all monetary resources used by this facility on staff salaries over the course of a year.<br>नीचे सूचीबद्ध प्रत्येक श्रेणी के लिए, कृपया इस केन्द्र द्वारा पिछले वित्त वर्ष के दौरान खर्च किया गया कुल व्यय बतायें। इसमें एक वर्ष से अधिक स्टाफ के वेतन पर इस केन्द्र द्वारा प्रयुक्त सभी मौद्रिक संसाधन शामिल है.		**Show If SAL_REC == 1**	ALL	Rajasthan
			Last Fiscal Year पिछला वित्त वर्ष			
PS2_SAL_	IHME	**TOTAL amount spent on all staff salaries/wages for the previous fiscal year**<br>पिछले वित्त वर्ष में सभी कर्मचारियों के वेतन/मजदूरी पर खर्च कुल धनराशि				
		Doctors<br>डाक्टर				

Unique Variable Name(s)	Source (Year & Item # if Known)			Item Conditions	Platform	State-Specific Modifications
		Nurses<br>नर्स				
		Matron मैट्रन				
		Ward Nurse वार्ड नर्स				
		Ward Sister वार्ड सिस्टर				
		Other paramedical staff (excluding nurses)<br>अन्य पैरामेडिकल कर्मचारी (नर्सों को छोडकर)				
		Non-medical staff<br>गैर चिकित्सकीय कर्मचारी				
		**Staff (3) - Benefits for <u>Current</u> Employees:**<br>कर्मचारी (3) वर्तमान कर्मचारियों के लाभ<br><br>Please note that employee benefits include contributions to provident fund, health benefits, medical insurance, pensions, tuition reimbursement, housing allowances, leave allowances, continuing medical education expenses, incentive pay, and/or educational support for children of employees.<br>कृपया ध्यान दें कि कर्मचारी लाभ में भविष्य निधि, स्वास्थ्य लाभ, मेडिकल बीमा, पेंशन, ट्यूशन प्रतिपूर्ति, आवास भते, छुट्टी भता, सतत मेडिकल शिक्षा के खर्च, प्रोत्साहन भुगतान, और / या कर्मचारियों के बच्चों के लिए शैक्षिक सहायता करने के लिए योगदान शामिल है.				
TAB_INSTRUCT_EXP_PERS3<br><br>EXP_PERSONNEL3			Last Fiscal Year पिछला वित्त वर्ष	required; show-if #BEN_REC ==1; column headings are populated by FY end dates range input into Collection "C_FINANCIAL_YRS";	ALL	Rajasthan
PS3_BENN_CURR_	IHME	**TOTAL amount spent on all staff benefits for current employees for the previous fiscal year**<br>पिछले वित्त वर्ष के लिए वर्तमान कर्मचारियों के लिए लाभ पर खर्च कुल धनराशि बतायें				
		Doctors<br>डाक्टर				
		Nurses<br>नर्स				
		Matron मैट्रन				
		Ward Nurse वार्ड नर्स				
		Ward Sister वार्ड सिस्टर				
		Other paramedical staff (excluding nurses)<br>अन्य पैरामेडिकल कर्मचारी (नर्सों को छोडकर)				

Unique Variable Name(s)	Source (Year & Item # if Known)			Item Conditions	Platform	State-Specific Modifications
		Non-medical staff<br>गैर चिकित्सकीय कर्मचारी				
		**Staff (4) - Benefits for <u>Former</u> Employees:**<br>कर्मचारी (4) पूर्व कर्मचारियों के लिए लाभ<br><br>Please note that employee benefits include contributions to provident fund, health benefits, medical insurance, pensions, tuition reimbursement, housing allowances, leave allowances, continuing medical education expenses, incentive pay, and/or educational support for children of employees<br>कृपया ध्यान दें कि कर्मचारी लाभ में भविष्य निधि, स्वास्थ्य लाभ, मेडिकल बीमा, पेंशन, ट्यूशन प्रतिपूर्ति, आवास भते, छुट्टी भता, सतत मेडिकल शिक्षा के खर्च, प्रोत्साहन भुगतान, और / या कर्मचारियों के बच्चों के लिए शैक्षिक सहायता करने के लिए योगदान शामिल है.				
TAB_INSTRUCT_EXP_PERS4<br><br>EXP_PERSONNEL4			Last Fiscal Year	required; show-if #BENFORM_REC ==1; column headings are populated by FY end dates range input into Collection "C_FINANCIAL_YRS";	ALL	Rajasthan
PS3_BENN_FORM_FY1_1<br>PS3_BENN_FORM_FY5_1	IHME	**TOTAL amount spent on all staff benefits for former employees**<br>पूर्व कर्मचारियों के लिए सभी कर्मचारी लाभों पर खर्च की गयी धनराशि				
EXP_COMMENTS		Please use this space to identify any funds that aren't included in these categories: ____<br>कृपया इस जगह को उस फण्ड की पहचान के लिए इस्तेमाल करें जिसे इन कैटेगरी में शामिल नहीं किया गया है				
COMMENTS_SECTION3A	IHME	Comments for Section 3: Finances		page break	ALL	None
		**Section 4: Facility Revenues**				
		This section is primarily designed to capture information about this facility's revenues. Please complete this section with a facility accountant, administrator or other individual who is well-informed about the finances to this health<br>**यह अनुभाग खास तौर पर केंद्र के राजस्व के बारे में जानकारी इकठ्ठा करने के लिए बनाया है। कृपया इस अनुभाग को केंद्र लेखाकार, प्रबंधक या किसी अन्य व्यक्ति जो इस स्वास्थ्य केंद्र के वित्त के बारे में जानता हो, उसके सहयोग से पूरा करें**				
REV_MONTH_YEAR		Are facility revenues recorded by month or by year?<br>क्या केन्द्र की आय को महीने या साल में रिकार्ड किया जाता है?<br>☐ 1=By Month महीने के अनुसार<br>☐ 2=By Year साल के अनुसार				

Unique Variable Name(s)	Source (Year & Item # if Known)			Item Conditions	Platform	State-Specific Modifications
TAB_INSTRUCT_REVENUE<br><br>REVENUE	IHME<br>AHA<br>DHS SPA	*Display as a table:*<br>**Total Revenue:**<br>कुल राजस्व<br><br>Please report the <u>total revenue</u> this facility collected during the previous fiscal year. This includes all monetary resources acquired by this facility from all sources, and for all purposes.<br>कृपया बतायें कि इस केन्द्र ने पिछले वित्त वर्ष के दौरान कुल कितनी आय की। इसमें इस केन्द्र द्वारा सभी स्रोतों से सभी आर्थिक संसाधनों, सभी उद्देश्यों को शामिल करें।<br>Please note, for all tables such as the one below, use the "Tab" key to move to the next cell space<br>कृपया नोट करें कि सभी तालिकाओं के लिए अगले सेल पर जाने के लिए टैब की का प्रयोग करें<br><br>Enter the following codes, if necessary: यदि आवश्यक हो तो निम्न कोड लिखें<br>0=No revenue कोई आय नहीं<br>-1=Don't know नहीं जानते<br>-2=Decline to respond जवाब देने से इंकार<br><br>All amounts should be listed in Indian rupees सभी धनराशि रुपये में होनी चाहिए		required; column headings are populated by FY end dates range input into Collection "C_FINANCIAL_YRS";	ALL	Currency
			Last Year			
REV_ABS		**TOTAL revenue (all payers) कुल राजस्व**				
		(1) Reimbursements from Ministry of Health and Family Welfare, Government of India (NRHM)(JSY, RCH II, Immunization)<br>स्वास्थ्य एवं परिवार कल्याण मंत्रालय, भारत सरकार (NRHM) द्वारा प्रतिपूर्ति			ALL	State Specific
		(a) RCH-II<br>आरसीएच - 2			ALL	State Specific
		(b) NRHM initiative (ASHA, Untied funds, Hospital strengthening, MMU, procurement,C42 etc)<br>एनआरएचएम (आशा, संयुक्त फण्ड, अस्पताल को मजबूत करने के लिए, एमएमयू, प्राप्ति, सी42 आदि)			ALL	State Specific
		(c) Immunization प्रतिरक्षण			ALL	State Specific
		(d) IDD आईडीडी			ALL	State Specific
		(e) IDSP आईडीएसपी			ALL	State Specific
		(f) NVBDCP एनवीबीडीसीपी			ALL	State Specific
		(g) NLEP एनएलईपी			ALL	State Specific
		(h) NPCB एनपीसीबी			ALL	State Specific
		(i) RNTCP आरएनटीसीपी			ALL	State Specific

Unique Variable Name(s)	Source (Year & Item # if Known)			Item Conditions	Platform	State-Specific Modifications
		(2) Reimbursement from State Department of Health and Family Welfare (state funds)<br>स्वास्थ्य एवं परिवार कल्याण के राज्य विभाग द्वारा प्रतिपूर्ति (राज्य निधि)			ALL	State Specific
		(3) Other donor agency or private organization अन्य डोनर एजेंसी या निजी संगठन			ALL	State Specific
		(4) Direct patient / out-of-pocket charges/fees प्रत्यक्ष रोगी/ जेब से खर्च की गयी /फीस			ALL	State Specific
		(5) Direct tuition/training payments प्रत्यक्ष ट्यूशन/ प्रशिक्षण भुगतान			ALL	State Specific
		(6) Research funding अनुसन्धान के लिए वित्त पोषण			ALL	State Specific
		(7) Reimbursement from public insurance सार्वजनिक बीमा द्वारा प्रतिपूर्ति			ALL	State Specific
		a. Rashtriya Swasthya Bima Yojana (RSBY)<br>राष्ट्रीय स्वास्थ्य बीमा योजना (आरएसबीवाई)			ALL	State Specific
		b. Chief Minister Relief Fund (CMRF)<br>मुख्यमंत्री राहत कोष (सीएमआरएफ)			ALL	State Specific
		(8) Reimbursement from private insurance निजी बीमा द्वारा प्रतिपूर्ति			ALL	State Specific
		(9) NACO/SACS नाको/ सैक्स			ALL	State Specific
		(10) Other अन्य				
ROUTINE_FEES_OSP		Please specify 'other': ___________________________<br>कृपया 'अन्य' को स्पष्ट करें				
FEES	IHME DHS SPA GENERAL INFO question 141	Does this facility have any routine user-fees or charges for patient services? This includes any fees, including those for registration or for client health records, or for diagnostics including charges for reports.<br>रोगी सेवाओं के लिए क्या इस केन्द्र में कोई नियमित उपयोगकर्ता शुल्क है? इसमें पंजीकरण या ग्राहक के स्वास्थ्य रिकॉर्ड के लिए, या नैदानिक जांच और उसकी रिपोर्ट का शुल्क सहित कोई भी शुल्क शामिल हैं.<br>(SELECT ALL THAT APPLY) जो लागू हों सभी को चुनें<br>☐ 3=Yes, registration fees हां, पंजीकरण शुल्क<br>☐ 2=Yes, fees for diagnostic or laboratory reports हां, जांच या प्रयोगशाला रीपोर्ट के लिए शुल्क<br>☐ 1 = Yes, fees for all services हां, सभी सेवाओं के लिए शुल्क<br>☐ 0=No fees at all बिलकुल भी कोई शुल्क नहीं<br>☐ -1=Don't know नहीं जानते<br>☐ -2=Decline to respond जवाब देने से इंकार		;		

Unique Variable Name(s)	Source (Year & Item # if Known)		Item Conditions	Platform	State-Specific Modifications
ROUTINE_FEES		Do ALL patients pay routine user-fees or charges for patient services?<br>क्या रोगी सेवाओं के लिए सभी रोगी नियमित उपयोगकर्ता शुल्क का भुगतान करते हैं?<br>(SELECT ONE) एक चुनें<br>☐ 1=Yes हां<br>☐ 0=No, those below certain income do not pay नहीं, कुछ निश्चित आय से नीचे के लोग नहीं अदा करते<br>☐ 2=No one pays कोई भी अदा नहीं करता<br>☐ -1=Don't know नहीं जानते<br>☐ -2=Decline to respond जवाब से इंकार<br>☐ -3= Other, Please specify अन्य, कृपया स्पष्ट करें	Show if fees <>0		
ROUTINE_FEES_OSP		Please specify 'other': ___________________________<br>कृपया 'अन्य' को स्पष्ट करें			

C_PAT_FEES		**USER FEES**<br>प्रयोग शुल्क<br>Please ask for fees charged to patients who pay for services from the facility for the below services.<br>कृपया केन्द्र की निम्नलिखित सेवाओं के लिए रोगियों द्वारा भुगतान किए गए शुल्क के बारे में पूछें.<br>All fees should be entered in Indian rupees<br>सारा शुल्क रुपये में लिखें<br>Enter the following codes, if necessary: यदि आवश्यक हो तो ये कोड लिखें<br>0=No fee is charged कोई शुल्क नहीं लिया जाता<br>-1=Don't know नहीं जानते<br>-2=Decline to respond जवाब से इंकार		Show if FEES=1, 2, 3		Country specific terminology, categories will remain the same.
		**Service Fee *सेवा शुल्क***	**Fee *शुल्क***			
PAT_FEES		Registration पंजीकरण			All Public	
		Ambulance charges रोगी वाहन शुल्क			All Public	
		CT Scan सीटी स्केन			All Public Except PHC and SHC	
		ECG ईसीजी			All Public Except PHC and SHC	
		Ultrasound अल्ट्रासाउण्ड			All Public Except PHC and SHC	
		X-rays एक्सरे			All Public Except PHC and SHC	
		MRI एमआरआई			All Except PHC and CHC	
		Plasma glucose test प्लाज्मा ग्लूकोज टेस्ट			All Except PHC and CHC	

		HbA1c test एचबीए1सी टेस्ट			All Except PHC and CHC	
		Fasting lipid profile फास्टिंग लिपिड प्रोफाइल			All Except PHC and CHC	
		Consultation for hypertension उच्चरक्तचाप के लिए परामर्श			All	
		Consultation for diabetes डाइबिटीज के लिए परामर्श			All	
		Pediatric consultation /registration शिशु रोगों का परामर्श/ पंजीकरण			All Private	
		Adult consultation /registration वयस्कों का परामर्श/ पंजीकरण			All Private	
		Adult overnight stay or overnight observation (one night IP admission charges)<br>वयस्कों का एक रात रुकना या एक रात का अवलोकन (एक रात आईपी भर्ती शुल्क)			All Private	
		**Comments for Section 4: Revenues** ***सेक्शन 4 के लिए टिप्पणी : आय***				
		**Section 5: Facility Staff Characteristics**				
		This section is primarily designed to capture information about this facility's staff. Please complete this section with a facility human resources administrator or other individual who is well-informed about staff employed at this facility.<br>**यह अनुभाग खास तौर पर केंद्र के कर्मचारियों के बारे में जानकारी लेने के लिए बनाया गया है इस अनुभाग को केंद्र मानव संसाधन प्रबंधक या किसी अन्य व्यक्ति जो नियुक्त किये हुए कर्मचारियों के बारे में जानता हो उसके सहयोग से पूरा करें.**				
INPUT_PS_FTE_HRS		*(Display as table)*<br>Average hours worked per week per individual staff member by staff type.<br>प्रति स्टाफ प्रति सप्ताह किए गए काम के औसत घंटे<br>Enter the following codes, if necessary: यदि आवश्यक हो तो ये कोड लिखें<br>-1=Don't know नहीं जानते<br>-2=Decline to respond जवाब से इंकार				
		**Staff Category स्टाफ श्रेणी**	**Average Hours worked per week per individual staff member**<br>**प्रति सप्ताह प्रत्येक कर्मचारी ने औसतन कितने घण्टे काम किया**		ALL	None
FTE_HRS_DRS		Doctor<br>डाक्टर				
FTE_HRS_NRS		Nurse(excluding ANM)<br>नर्स (एएनएम को छोडकर)				
FTE_HRS_ANM		**ANM/Village Health Nurse**<br>एएनएम / ग्राम स्वास्थ्य नर्स				
		**Female Healthworkers** महिला स्वास्थ्य कार्यकर्ता				
		**Village Healthworkers** ग्राम स्वास्थ्यकार्यकर्ता				
		**Traditional Birth Attendants** दाई				
		Matron मैट्रन				
		**Ward Nurse** वार्ड नर्स				
		Ward Sister वार्ड सिस्टर				
FTE_HRS_OMP		Other paramedical staff (excluding nurses and ANM)<br>अन्य पैरामेडिकल कर्मचारी (नर्सों और एएनएम को छोडकर)				

FTE_HRS_NMP		Non-medical staff<br>गैर चिकित्सकीय कर्मचारी			
TAB_INSTRUCT_PS_FTE_TOT<br><br>C_INPUT_PS_FTE_TOT		*Display as a table:*<br><br>**Total Staff at this facility:**<br>इस केन्द्र पर कुल कर्मचारी<br><br>**Total staff that work at this facility. All individuals who work at this facility should be counted.**<br>**कुल स्टाफ जो इस केन्द्र में काम करते हैं. इस केन्द्र में काम करने वाले सभी व्यक्तियों को गिना जाना चाहिए.**<br>For each occupational category listed below please indicate the number of persons who worked at this facility at the end of each of the previous fiscal year. Include all staff who worked at this facility irrespective of the source of payment and any volunteer.<br>नीचे दी गयी प्रत्येक व्यावसायिक कैटेगरी के लिए कृपया बतायें कि प्रत्येक पिछले वित्त वर्ष के अन्त में इस केन्द्र पर कितने व्यक्तियों ने काम किया। इस केन्द्र पर काम करने वाले सभी कर्मचारियों को शामिल करें, इससे कोई मतलबनहीं कि कोई वालंटियर है या उसको अदायगी का स्रोत क्या है।<br>Please indicate the total number of staff in each job category during the last fiscal year.<br>कृपया बतायें कि पिछले वित्त वर्ष में प्रत्येक जॉब कैटेगरी में कुल कितने लोग थे।<br><br>Enter the following codes, if necessary: यदि आवश्यक हो तो ये कोड लिखें<br>0=No staff in this job category इस जॉब कैटेगरी में कोई कर्मचारी नहीं<br>-1=Don't know नहीं जानते<br>-2=Decline to respond जवाब देने से इंकार			
		**Total staff working at this facility इस केंद्र पर काम करने वाले कुल कर्मचारी**			
		a. **TOTAL Doctors कुल मेडिकल स्टाफ (डॉक्टर)**			
		b. **TOTAL Nurses excluding ANM कुल नर्स**			
		c. **TOTAL ANM/Village Health Nurse एएनएम / ग्राम स्वास्थ्य नर्स**			
		**d. TOTAL Female Healthworkers** कुल महिला स्वास्थ्यकार्यकर्ता			
		**e. TOTAL Village Healthworkers** कुल ग्राम स्वास्थ्यकार्यकर्ता			
		**f. TOTAL Traditional Birth Attendants** कुल दाई			
		**g.** TOTAL Matrons कुल मैट्रन			
		**h. TOTAL Ward Nurse** कुल वार्ड नर्स			
		**i.** TOTAL Ward Sister कुल वार्ड सिस्टर			
		j. **TOTAL Other Paramedical Staff excluding nurses and ANM कुल अन्य पैरामैडिकल स्टाफ**			

		k. **TOTAL Other Non-Medical Staff कुल अन्य गैर - मेडिकल स्टाफ**											
FAC_TRAIN	IHME	Have there ever been any training sessions organized by this facility in the last 12 months?<br>क्या इस केन्द्र द्वारा कभी कोई प्रशिक्षण सत्र का आयोजन किया गया है?<br>(SELECT ONE) एक विकलप चुनें<br>☐ 1=Yes हां<br>☐ 0=No नहीं<br>☐ -1=Don't Know नहीं जानते<br>☐ -2=Decline to respond जवाब देने से इंकार											
STAFF_TRAIN		Does the staff working at this facility go for training elsewhere?<br>क्या इस केन्द्र पर काम कर रहे कर्मचारी प्रशिक्षण के लिए कहीं और जाते है?<br>(SELECT ONE) एक विकलप चुनें<br>☐ 1=Yes हां<br>☐ 0=No नहीं<br>☐ -1=Don't Know नहीं जानते<br>☐ -2=Decline to respond जवाब देने से इंकार											
TAB_INST_INPUT_STAFFING		**Please click the button bellow and fill out the tables for Health Services and Administrive Support Services Provided**<br>कृपया नीचे की बटन पर क्लिक करें तथा स्वास्थ्य सेवाओं और दी गयी प्रशासनिक सेवा सहयोग के लिए तालिका भरें<br>**Health Services Provided: Staffing**<br>स्वास्थ्य सेवाएं दी गयी - कर्मचारी<br><br>For each service listed below, please provide how many hours per day of the week the services is offered and the average number of staff providing this services on a typical day.<br>नीचे दी गयी प्रत्येक सर्विस के लिए, कृपया बतायें कि सप्ताह में रोजाना कितने घण्टे तक सेवा दी जाती है और एक सामान्य दिन में औसतन कितने कर्मचारी इस सेवा को देते हैं।<br>Enter the following codes, if necessary: आवश्यक होने पर निम्न कोड लिखें -<br>0=Service not offered/no staff in this category staffing this clinic/service<br>सेवा नहीं दी जाती/ इस क्लीनिक/सर्विस में इस कैटेगरी के कर्मचारी नहीं हैं<br>-1=Don't know नहीं जानते<br>-2=Decline to respond जवाब देने से इंकार											
C_INPUT_STAFFING			How many hours provided on **Monday**? सोमवार को कितने घण्टे दी जाती है?	How many hours provided on **Tuesday**? मंगलवार को कितने घण्टे दी जाती है?	How many hours provided on **Wednesday**? बुद्धवार को कितने घण्टे दी जाती है?	How many hours provided on **Thursday**? गुरुवार को कितने घण्टे दी जाती है?	How many hours provided on **Friday**? शुक्रवार को कितने घण्टे दी जाती है?	How many hours provided on **Saturday**? शनिवार को कितने घण्टे दी जाती है?	How many hours provided on **Sunday**? रविवार को कितने घण्टे दी जाती है?	What is the typical number of staff providing this service on a typical working day? एक सामान्य दिन में सामान्य रूप से कितने कर्मचारी सेवाएं देते हैं?			
		General cardiology<br>हृदय विज्ञान											All except PHC and SHC

		Accident, trauma & emergency दुर्घटना, ट्रामा और आपातकालीन											All except SHC
		Ophthalmology नेत्र विज्ञान											All except PHC and SHC
		Paediatric शिशु चिकित्सा											All except SHC
		General anesthesiology जनरल निश्चेतनाविज्ञान											All except PHC and SHC
		Blood bank रक्त बैंक											All except SHC
		DOTS treatment डॉट्स उपचार											All
		STI/HIV एसटीआई / एचआईवी											All except SHC
		Immunization टीकाकरण											All
		Internal/General medicine आंतरिक / जनरल दवा											All except PHC and SHC
		Orthopedic हड्डी रोग											All except PHC and SHC
		Pharmacy फ़ार्मेसी											All except SHC
		Oncology आनकोलोजी											All except PHC and SHC
		Alternative medicine e.g. Siddha, Homeopathy etc. वैकल्पिक चिकित्सा जैसे सिद्ध, होम्योपैथी आदि											All except SHC
		Diagnostic / medical imaging e.g. x-ray, CT Scan, MRI etc. निदान / मेडिकल इमेजिंग उदा. एक्स - रे, सीटी स्कैन, एमआरआई आदि											All except PHC and SHC
		Laboratory services प्रयोगशाला सेवाएं											All except SHC

		Preventative services (Smoking cessation, weight management, etc.)<br>रोकथाम सेवाएं (धूम्रपान बन्द करना, वजन नियंत्रित रखना आदि)						All
		How many days of outreach are conducted in a typical month एक सामान्य महीने में कितने दिनों के आउटरीच संचालित किये जाते हैं<br>(NUMERIC RESPONSE)						
		How many staff typically participate in outreach? आउटरीच में सामान्य रूप से कितने कर्मचारी भाग लेते हैं?<br>(NUMERIC RESPONSE)						
TAB_INST_INPUT_ADM_TEAMS		**Administrative and Support Services Provided: Staffing**<br>दी गयी प्रशासनिक और सपोर्ट सेवाएं : कर्मचारी<br>For each administrative service listed below, please provide how many days per week the service is available, how many hours per typical day the service is offered, and the typical number of staff staffing/providing this services on a typical day.<br>नीचे सूचीबद्ध प्रत्येक प्रशासनिक सेवा के लिए, कृपया बतायें कि सेवा प्रति सप्ताह कितने घंटे उपलब्ध है, किसी सामान्य दिन में कितने घंटे सेवा प्रदान की जाती है, और किसी सामान्य दिन यह सेवा प्रदान/ स्टाफ करने वाले स्टाफ की सामान्य संख्या.<br>Enter the following codes, if necessary: आवश्यक होने पर निम्न कोड लिखें -<br>1=Service Provided and staffed सेवा दी जाती है और कर्मचारी हैं<br>0=Service not offered/no staff in this category staffing this clinic/service सेवा नहीं दी जाती/ इस क्लीनिक/सर्विस में इस कैटेगरी के कर्मचारी नहीं हैं<br>-1=Don't know नहीं जानते<br>-2=Decline to respond जवाब देने से इंकार						
C_INPUT_ADM_TEAMS		Service सेवा	How many days per week is this service available? सप्ताह में कितने दिन यह सेवा उपलब्ध है?	How many hours per day is this service typically offered?<br>एक दिन में सामान्य रूप से कितने घण्टे यह सेवा दी जाती है?	What is the typical number of staff providing this service on a typical day?<br>एक सामान्य दिन में इस सेवा के लिए कितने कर्मचारी काम करते हैं?			
TEAMS_ADMIN_DPW_<br>TEAMS_ADMIN_HPD_<br>TEAMS_ADMIN_STAFFPD_	"	Accounting / billing and collection<br>एकाउंटिंग/ बिलिंग और एकत्रीकरण				All except SHC		
…	"	Patient review committee<br>रोगी समीक्षा समिति				All except PHC, and SHC		
	"	Medical records<br>चिकित्सा रिकार्ड				All except PHC, and SHC		
	"	Security<br>सुरक्षा				All except PHC, and SHC		

STAFF_POOR_PERF	INSP	Does the facility take any of the following actions in the case of poor staff performance?<br>क्या यह केन्द्र कर्मचारियों के खराब प्रदर्शन के मामले में निम्नलिखित से कोई भी कार्रवाई करता है?<br>(SELECT ALL THAT APPLY) (एक से अधिक उत्तर सम्भव)<br>☐ 1=Verbal warning मौखिक चेतावनी<br>☐ 2=Written warning from facility केन्द्र द्वारा लिखित चेतावनी<br>☐ 3=Written warning from Ministry of Health स्वास्थ्य मंत्रालय की ओर से लिखित चेतावनी<br>☐ 4=Reduce pay कम भुगतान<br>☐ 5=Less desirable work schedules कम वांछनीय कार्य कार्यक्रम<br>☐ 6= Termination निष्कासन<br>☐ 7= Confidential report in staff file स्टाफ फ़ाइल में गोपनीय रिपोर्ट<br>☐ 8=Other अन्य-<br>☐ 9= No action taken कोई कार्यवाही नहीं की गयी<br>☐ -1=Don't Know नहीं जानते<br>☐ -2=Decline to respond जवाब देने से इंकार	Page break	ALL	
STAFF_POOR_PERF_OSP		Please specify other action taken in the case of poor staff performance: ________________<br>कृपया बतायें कि कर्मचारियों के खराब प्रदर्शन की स्थिति में और कौन सी कार्यवाही की गयी			
PBFS	INSP	Does this facility offer any type of Performance Based incentives?<br>क्या यह केन्द्र किसी भी प्रकार का प्रदर्शन आधारित प्रोत्साहन प्रदान करता है?<br>(SELECT ONE) एक विकल्प चुनें<br>☐ 1=Yes हां<br>☐ 0=No नहीं<br>☐ -1=Don't Know नहीं जानते<br>☐ -2=Decline to respond जवाब देने से इंकार		ALL	
		Are the Performance Based Incentives related to non-communicable diseases?<br>क्या कार्यप्रदर्शन से संबंधित प्रोत्साहन गैर संक्रामक रोगों से संबंधित है?<br>(SELECT ONE) एक विकल्प चुनें<br>☐ 1=Yes हां<br>☐ 0=No नहीं<br>☐ -1=Don't Know नहीं जानते<br>☐ -2=Decline to respond जवाब देने से इंकार			

PBFS_WHOM		To which cadre of staff is the incentive system applicable?<br>स्टाफ के किस कैडर पर प्रोत्साहन प्रणाली लागू होता है?<br>(SELECT ALL THAT APPLY) (एक से अधिक उत्तर सम्भव)<br>☐ 1=All cadres सभी कैडर<br>☐ 2=Doctors डाक्टर<br>☐ 3=ASHA workers आशा कार्यकर्त्री<br>☐ 4=Nurses नर्स<br>☐ 5=Others अन्य<br>☐ 0=None कोई नहीं<br>☐ -1=Don't know नहीं जानते<br>☐ -2=Decline to respond जवाब देने से इंकार	Show if #PFB==1; select one		
		**Financial Audit Information**<br>वित्तीय आडिट संबंधी जानकारी			
FAC_AUDIT		Is the annual PIP reports for the most recent fiscal year available?<br>क्या पिछले वित्तीय वर्ष का वित्तीय लेखा परीक्षा उपलब्ध है?<br>(SELECT ONE) एक विकल्प चुनें<br>☐ 1=Yes हां<br>☐ 0=No नहीं<br>☐ -1=Don't Know नहीं जानते<br>☐ -2=Decline to respond जवाब देने से इंकार	Page break	ALL	
		Comments for Section 5: Staff Characteristics			
		**Section 6: Facility Management**			
INTRO_SECTION5A	IHME	This section is designed to capture general information about this facility, its infrastructure and other relevant characteristics. Please complete this section with a facility administrator, manager or other individual who is well-informed about the overall characteristics of this health facility.<br>यह अनुभाग इस केंद्र की आधारभूत संरचना और अन्य प्रासंगिक विशेषताओं कि सामान्य जानकारी लेने के लिए बनाया गया है कृपया इस अनुभाग को केंद्र संचालक, प्रबन्धक या कोई ऐसा व्यक्ति जो इस केंद्र के बारे में जानता हो, उसकी सहायता से भरें		ALL	

FAC_HRS_MON<br>FAC_HRS_TUES<br>FAC_HRS_WED<br>FAC_HRS_THURS<br>FAC_HRS_FRI<br>FAC_HRS_SAT<br>FAC_HRS_SUN	IHME	How many hours per day is the facility open on each day of the week?<br>यह केंद्र सप्ताह के प्रत्येक दिन कितने घंटे खुला रहता है?<br>If necessary enter -1 for missing/no data (यदि आवश्यक हो तो अनुपलब्ध डाटा के लिए -1 भरें)<br>(ENTER NUMBER OF HOURS) (कृपया घंटों की संख्या भरें)<br>Monday सोमवार : ___<br>Tuesday मंगलवार : ___<br>Wednesday बुद्धवार : ___<br>Thursday गुरुवार : ___<br>Friday शुक्रवार : ___<br>Saturday शनिवार : ___<br>Sunday रविवार : ___	required;	only at Subcenter, PHC and private	Country specific
HOSP_HRS_MON<br>HOSP_HRS_TUES<br>HOSP_HRS_WED<br>HOSP_HRS_THURS<br>HOSP_HRS_FRI<br>HOSP_HRS_SAT<br>HOSP_HRS_SUN	IHME	How many hours per day is the outpatient services/general services clinic of thisfacility open on each day of the week?<br>इस केंद्र की वाह्य रोगी सेवाएँ / सामान्य सेवाएँ सप्ताह के प्रत्येक दिन कितने घंटे खुली रहती हैं?<br>If necessary enter -1 for missing/no data (यदि आवश्यक हो तो अनुपलब्ध डाटा के लिए -1 भरें)<br>(ENTER NUMBER OF HOURS) (कृपया घंटों की संख्या भरें)<br>Monday सोमवार : ___<br>Tuesday मंगलवार : ___<br>Wednesday बुद्धवार : ___<br>Thursday गुरुवार : ___<br>Friday शुक्रवार : ___<br>Saturday शनिवार : ___<br>Sunday रविवार : ___	required;	ALL	None
accessible_weeks	IHME	How many weeks per year is the facility not accessible to some patients within this facility's catchment area because of flooding or other environmental reasons (such as snow)?<br>प्रति वर्ष कितने सप्ताह यह केन्द्र अपने क्षेत्र के भीतर बाढ़ या अन्य पर्यावरणीय कारणों (जैसे बर्फ) से कुछ रोगियों के लिए सुलभ नहीं हो पाता है?<br>(SELECT ONE)<br>☐ 1=Enter number of weeks: ___ सप्ताह की संख्या लिखें<br>-1=Don't know नहीं जानते<br>-2=Decline to respond जवाब से इंकार	required; select one; range: 0-52;	ALL	None
watershortage		In a typical year, is there a time of year when there is a severe shortage or lack of water at this facility?<br>किसी सामान्य वर्ष में, साल का कोई ऐसा समय होता है जब इस केन्द्र में पानी की भारी कमी होती है?<br>(SELECT ONE) एक विकल्प चुनें<br>☐ 1=Yes हां<br>☐ 0=No नहीं<br>☐ -1=Don't Know नहीं जानते<br>☐ -2=Decline to respond जवाब देने से इंकार	required; page break	ALL	
examination_rooms		How many examination rooms are there in this facility? इस केन्द्र में कितने जांच कक्ष हैं?<br>(An examination room is room where a medical provider receives and treats patients, and otherwise practices medicine.)<br>(जांच कक्ष वह कमरा है जहां चिकित्सक मरीज से मिलकर उसका उपचार करता है और मेडिसिन की प्रैक्टिस करता है)<br>(SELECT ONE)<br>☐ 1=Total number of examination rooms: ____ परीक्षण कक्षों की कुल संख्या		ALL	None

		-1=Don’t Know नहीं जानते<br>-2=Decline to respond जवाब देने से इंकार				
inpatient		Does this facility provide inpatient care?<br>क्या यह केन्द्र अन्तःरोगी देखभाल प्रदान करता है?<br>(SELECT ONE) एक विकल्प चुनें<br>☐ 1=Yes हां<br>☐ 0=No नहीं<br>☐ -1=Don’t Know नहीं जानते<br>☐ -2=Decline to respond जवाब देने से इंकार			ALL	
fac_beds	IHME	How many inpatient beds are there in this facility?<br>इस केन्द्र में कितने अन्तःरोगी बिस्तर हैं?<br>(SELECT ONE) एक विकल्प चुनें<br>☐ 1=Total number of beds: ____ कुल बिस्तरों की<br>-1=Don’t Know नहीं जानते<br>-2=Decline to respond जवाब देने से इंकार		required; select one; range: 0-1500 soft prompt; show if INPATIENT=1	ALL	None
IP_ROOMS_BD		Please Click the following button<br>कृपया नीचे की बटन पर क्लिक करें<br>(Display as table)<br><br>Of the total rooms where patients typically spend the night how many hold only 1 bed? How many hold 2 beds? How many hold 4 or more beds?<br>कुल कमरों में से जहां रोगियों को आम तौर पर रात बिताना होता है कितनों में 1 बिस्तर है? कितनों में 2 बिस्तर है? कितनों में 4 या अधिक बिस्तर है?<br>Enter the following codes, if necessary: आवश्यक होने पर निम्न कोड लिखें -<br>-1=Don’t know नहीं जानते<br>-2=Decline to respond जवाब देने से इंकार		Current.inpatient = 1	ALL	Might need to include wards with many beds in some countries
		**Number of rooms with:** ...... वाले कमरों की संख्या	**Number of rooms** कमरों की संख्या			
		Private room: 1 bed प्राइवेट कमरे - 1 बेड				
		Private room: 2-4 beds प्राइवेट कमरे - 2-4 बेड				
		General wards जनरल वार्ड				
main_inf	DHS SPA	Does this facility perform routine maintenance and repair of **infrastructure**?<br>क्या यह केन्द्र **आधारभूत संरचना का** नियमित रखरखाव और मरम्मत का कार्य करता है?<br>☐ 1=Yes हां<br>☐ 0=No नहीं<br>☐ -1=Don’t Know नहीं जानते<br>☐ -2=Decline to respond जवाब देने से इंकार		required; select one;	ALL	None

maint_equ	IHME	Does this facility perform routine maintenance and repair of **major equipment** such as a generator, refrigerator, sterilization equipment, or other large medical equipment?<br>क्या यह केन्द्र **प्रमुख उपकरणों** जैसे कि जनरेटर, रेफ्रिजरेटर, जर्मरोधी उपकरण, या अन्य बड़े चिकित्सा उपकरणों का नियमित रखरखाव और मरम्मत का कार्य करता है?<br>☐ 1=Yes हां<br>☐ 0=No नहीं<br>☐ -1=Don't Know नहीं जानते<br>☐ -2=Decline to respond जवाब देने से इंकार<br>☐ -3=Not applicable, this facility does not have any major equipment that requires routine maintenance and repair केंद्र के पास कोई भी प्रमुख उपकरण नहीं है जिसे नियमित रख रखाव और मरम्मत कि आवश्यकता होती है	required; select one;	ALL	None
system_1	IHME	Does this facility have a functional electricity connection?<br>क्या इस केन्द्र में एक कार्यात्मक बिजली कनेक्शन है?<br>(SELECT ONE) एक विकल्प चुनें<br>☐ 1=Yes हां<br>☐ 0=No नहीं<br>☐ -1=Don't Know नहीं जानते<br>☐ -2=Decline to respond जवाब देने से इंकार		ALL	None
electricity_hours		Usually during a typical day, how many hours is there electricity available at the facility irrespective of the source?<br>आम तौर पर एक सामान्य दिन पर, इस केन्द्र पर किसी भी स्रोत से कितने घंटे बिजली उपलब्ध रहती है?<br>(SELECT ONE) एक विकल्प चुनें<br>☐ 1=Total number of hours: कुल घंटों की संख्या ____<br>-1=Don't Know नहीं जानते<br>-2=Decline to respond जवाब देने से इंकार	current.inpatient=1	ALL	None
electricity_days	IHME<br>DHS SPA	During the past <u>week</u> on how many days was the electricity not available for <u>at least two hours</u> from any source during a time the facility was open for services (including emergency services)?<br>Enter "0" if there were no interruptions in electricity.<br>पिछले एक सप्ताह के दौरान जब केन्द्र सेवाओं के लिए खुला हुआ था (आपातकालीन सेवाओं सहित), किसी भी स्रोत से कितने दिन <u>कम से कम दो घंटे के लिए</u> बिजली उपलब्ध नहीं थी?<br>(SELECT ONE) एक विकल्प चुनें<br>☐ 1=Total number of days कुल दिनों की संख्या ____<br>-1=Don't Know नहीं जानते<br>-2=Decline to respond जवाब देने से इंकार	current.inpatient=1	ALL	None
internet		Does this facility have a functional internet connection?<br>क्या इस केन्द्र में चालू इंटरनेट कनेक्शन है?<br>(SELECT ONE) एक विकल्प चुनें<br>☐ 1=Yes हां<br>☐ 0=No नहीं<br>☐ -1=Don't Know नहीं जानते<br>☐ -2=Decline to respond जवाब देने से इंकार		ALL	None

ATQ_HR<br><br>PAPER<br>ELEC<br>PAPERACC<br>ELECACC<br>NONE<br>DK<br>DTR<br>NA	IHME<br>AHA<br>Rand	Please indicate if this facility utilizes any of the following health record systems:<br>कृपया सूचित करें कि क्या यह केंद्र निम्नलिखित स्वास्थ्य रिकार्ड सिस्टम में से किसी एक का भी इस्तेमाल करता है:<br>(SELECT ALL THAT APPLY) (एक से अधिक उत्तर सम्भव)<br>☐ 4=Paper-based health record system कागजात आधारित हेल्थ रिकार्ड सिस्टम<br>☐ 3=Electronic-based health record system इलेक्ट्रानिक आधारित हेल्थ रिकार्ड सिस्टम<br>☐ 0=None of the above इनमें से कोई नहीं<br>☐ -1=Don't know नहीं जानते<br>☐ -2=Decline to respond जवाब से इंकार	required;<br>select all,<br>excluding<br>NONE/DK/DTR/<br>NA;	ALL	None
watersource_t		What are the most commonly used sources of water used at this facility?<br>इस केंद्र पर सामान्य रूप से उपयोग किये जाने वाले पानी के स्रोत क्या है?<br>(SELECT ALL THAT APPLY) (एक से अधिक उत्तर सम्भव)<br>☐ 1=Piped into facility केन्द्र में पाइप से पानी की सुविधा<br>☐ 2=Well (public protected) कूआं (सार्वजनिक रूप से सुरक्षित)<br>☐ 3=Well (facility protected) कूआं (केन्द्र द्वारा सुरक्षित)<br>☐ 4=Well (unprotected कूआं (असुरक्षित)<br>☐ 5=Borehole बोरहोल<br>☐ 6=Hand pump हैण्डपम्प<br>☐ 7=Bottled water बोतल वाला पानी<br>☐ 8=Tanker truck टैंकर ट्रक<br>☐ 9=Rainwater बारिश का पानी<br>☐ 10=Other अन्य<br>☐ 11=No water source पानी का कोई स्रोत नहीं<br>☐ -1=Don't Know नहीं जानते<br>☐ -2=Decline to respond जवाब देने से इंकार<br>☐ -3=Not applicable नहीं लागू	required;<br>select all,<br>excluding<br>NONE/DK/DTR/<br>NA;	ALL	None
watersource_osp		Please specify 'other':<br>कृपया 'अन्य' को बतायें			
ROUT_ADMIN_MEETINGS	IHME<br>DHS SPA<br>GENERAL<br>INFO<br>question<br>118	Does this facility hold ***routine*** meetings to discuss facility managerial and administrative matters with just facility staff?<br>क्या यह केन्द्र केन्द्र के प्रबन्धकीय और प्रशासनिक मामलों की चर्चा के लिए केन्द्र के कर्मचारियों के साथ नियमित बैठकें करता है?<br>(SELECT ONE) एक विकल्प चुनें<br>☐ 1=Yes हां<br>☐ 0=No नहीं<br>☐ -1=Don't Know नहीं जानते<br>☐ -2=Decline to respond जवाब देने से इंकार		ALL	None

ADMIN_MEETINGS	IHME DHS SPA GENERAL INFO question 118	How often do these meetings, to discuss facility managerial and administrative matters, take place?<br>प्रशासनिक और प्रबंधकीय मामलों पर चर्चा के लिए ये बैठके कितने अंतराल पर होती है?<br>(SELECT ONE) एक विकल्प चुनें<br>☐ 5=Weekly साप्ताहिक<br>☐ 1=Bi-weekly or monthly दो सप्ताह में एक बार या मासिक<br>☐ 2=Every 2-3 months हर 2-3 महीने में<br>☐ 3=Every 4-6 months हर 4-6 महीने में<br>☐ 4=Less often than every 6 months or irregularly हर 6 महीने में एक बार से कम या अनियमित रूप से<br>☐ -1=Don't Know नहीं जानते<br>☐ -2=Decline to respond जवाब देने से इंकार	Show if #ROUT_ADMIN_MEETINGS=1	ALL	None
ROUT_ADMIN_MEETINGS_COMM		Does this facility hold ***routine*** meetings about facility activities or management issues with both facility staff and community members?<br>क्या यह केंद्र, केंद्र की गतिविधियों या प्रबंधन के मुद्दों के बारे में केंद्र के कर्मचरियों और समुदाय के सदस्यों के साथ नियमित बैठकें आयोजित करता है?<br>(SELECT ONE) एक विकल्प चुनें<br>☐ 1=Yes हां<br>☐ 0=No नहीं<br>☐ -1=Don't Know नहीं जानते<br>☐ -2=Decline to respond जवाब देने से इंकार<br>☐ -3=Not applicable नहीं लागू		All	None
ADMIN_MEETINGS_COMM	IHME DHS SPA GENERAL INFO question 122	How often do these meetings, about facility activities or management issues with both facility staff and community members, take place?<br>केन्द्र की गतिविधियों या प्रबंधन के मुद्दों के बारे में केन्द्र स्टाफ और समुदाय के सदस्यों के साथ कितनी बार ये बैठकें कितने अंतराल पर होती हैं?<br>(SELECT ONE) एक विकल्प चुनें<br>☐ 1=Monthly or more often मासिक या इससे ज्यादा<br>☐ 2=Every 2-3 months हर 2-3 महीने में<br>☐ 3=Every 4-6 months हर 4-6 महीने में<br>☐ 4=Less often than every 6 months or irregularly हर 6 महीने में एक बार से कम या अनियमित रूप से<br>☐ -1=Don't Know नहीं जानते<br>☐ -2=Decline to respond जवाब देने से इंकार<br>☐ -3=Not applicable नहीं लागू	Show if #ROUT_ADMIN_MEETINGS_COMM=1	All	None
WHO_ASSISTED	IHME	What were the positions of the respondents who helped you fill in this module of the survey?<br>जिस उत्तरदाता ने आपको सर्वे के इस माड्यूल को भरने में आपकी मदद की उसका पद क्या था?<br>(SELECT ALL THAT APPLY) (एक से अधिक उत्तर सम्भव)<br>☐ 1 =Facility Administrator केन्द्र प्रशासक<br>☐ 2 = Doctor डाक्टर<br>☐ 3 = Nurse नर्स<br>☐ 4 = Pharmacist फार्मासिस्ट<br>☐ 5= Laboratory Technition लैब टेक्नीशियन<br>☐ 6= Accountant एकाउंटेन्ट<br>☐ 7= Human Resource Manager मानव संसाधन प्रबन्धक<br>☐ 8= HMIS Manager एचएमआईएस मैनेजर<br>☐ 9= Clerk क्लर्क<br>☐ 10 = Other Staff, Specify अन्य कर्मचारी, स्पष्ट करें	required;	ALL	

COMMENTS_PERF	IHME	Your input is important to us, and will help us to better-tailor this instrument for collecting this valuable information.<br>Please use the space below to provide us with any feedback you may have regarding Survey 1: Finances and Inputs<br>आपका सहयोग हमारे लिए महत्वपूर्ण है, और बहुमूल्य जानकारी को इकट्ठा करने के लिए हमें इस प्रपत्र को बेहतर बनाने में मदद मिलेगी.<br>इस माड्यूल के संबंध में आपकी यदि कोई प्रतिक्रिया हो तो कृपया नीचे दिए गए स्थान का उपयोग करें: वित्त और इनपुट	NOT required; page break	ALL	

Unique Variable Name(s)	Source (Year & Item # if Known)	ENGLISH/HINDI<br>Question & Response Options with Values	Item Conditions	Platform	Site-Specific Modifications
	IHME	**Module 2: Direct Observation and Facility Supplies**			
		**Survey Log-In**			
		Enter District ID: ________________<br>जिले की पहचान संख्या लिखें			
CUSTOMID	IHME	Enter the CUSTOMID for this facility:________<br>इस केन्द्र का CUSTOMID प्रविष्ट करें: ____________________________	required;	ALL	none
FIELDSTAFF_ID<br>FIELDSTAFF_ID_TEXT	IHME	Enter your Interviewer ID: ______________<br>अपना साक्षात्कारकर्ताआईडी प्रविष्ट करें: ______________	required;<br>text only;<br>page break	ALL	None
		Introduction			
TIME_START		Survey Start Time. Please click 'Get Time' button<br>सर्वे शुरू करने का समय। कृपया 'गेट टाइम' बटन पर क्लिक करें<br>[Get Time]____________________			
PLATFORM_TYPE	IHME	Health service delivery platform type:<br>स्वास्थ्य सेवा प्रदान करने वाले प्लेटफॉर्म का प्रकार:<br>(SELECT ONE)<br>☐ 1= District Hospital (DH) जिला अस्पताल (डीएच)<br>☐ 2 = Civil Hospital (CH) सिविल अस्पताल (सीएच)<br>☐ 3 = Community Health Centre (CHC) सामुदायिक स्वास्थ्य केन्द्र (सीएचसी)<br>☐ 4 = Primary Health Centre (PHC) प्राथमिक स्वास्थ्य केन्द्र (पीएचसी)<br>☐ 5 = Sub Health Centre (SHC) उपस्वास्थ्य केन्द्र (एसएचसी)<br>☐ 6 = Private Hospital प्राइवेट अस्पताल<br>☐ 7= Private Clinic प्राइवेट क्लीनिक	PRELOAD;	ALL	Country specific platform types
Fac_private		Is this a private facility?<br>क्या यह प्राइवेट स्वास्थ्य केन्द्र है?<br>☐ 1=Yes हां<br>☐ 0=No नहीं			

Unique Variable Name(s)	Source (Year & Item # if Known)	ENGLISH<br>Question & Response Options with Values			Item Conditions	Plat-form	Site-Specific Modifications
		**Section 1: Laboratory Consumables and Equipment**<br><br>**Please complete this section with a laboratory technician or other individual who is well-informed about the medical equipment, medical consumables and facility information that is normally used in the laboratory or to perform lab tests.**<br>**कृपया इस खंड को प्रयोगशाला तकनीशियन या किसी अन्य व्यक्ति कि सहायता से भरें जो कि चिकित्सा उपकरणों , चिकित्सा उपभोग्य सामग्रियों और केंद्र की सूचना अच्छी प्रकार से जानता हो. जिसका उपयोग सामान्यतः प्रयोगशाला या प्रयोगशाला परीक्षण में होता है**					
TESTS_AVAIL		(show as table)<br>**Laboratory Tests: with lab tech/medical staff**<br>प्रयोगशाला परीक्षण - लैब टेक्नीशियन/ चिकित्सा कर्मचारी की मदद से<br><br>Please report if each of the following lab tests are:<br>कृपया बतायें यदि प्रत्येक निम्नलिखित प्रयोगशाला परीक्षण:<br><br>• Typically available सामान्य रूप से उपलब्ध<br>• If the test is performed at this facility. क्या टेस्ट इस केन्द्र पर किया जाता है<br><br>Response options for both columns: दोनों कालम के लिए जवाब के विकल्प<br>1=Yes हां<br>0=No नहीं<br>-1=Don't know नहीं जानते<br>-2=Decline to respond जवाब देने से इंकार					
		**Test** टेस्ट/परीक्षण	**Typically Available आम तौर पर उपलब्ध**	**Is the test performed within this facility?** क्या टेस्ट इस केन्द्र पर किया जाता है?			None
TESTS_AVAIL_TYP_1;TESTS_AVAIL_NOW_1							
		Urinalysis (dipstick and/or microscopy) पेशाब की जांच (डिपस्टिक और/या माइक्रोस्कोपी)				All except SHC	
		Hemoglobin test हीमोग्लोबिन टेस्ट				All except SHC	
		Complete blood count सम्पूर्ण ब्लड काउंट				All except SHC	
		Cross-match blood test क्रासमैच ब्लड टेस्ट				All except SHC	
		Blood grouping test ब्लड ग्रुपिंग टेस्ट				All except SHC	
		Liver function test लीवर फंक्शन टेस्ट				All except PHC and SHC	
		Renal/kidney function test गुर्दा/किडनी फंक्शन टेस्ट				All except SHC	
		Serum electrolytes सीरम इलेक्ट्रोलाइट्स				All except SHC	

Unique Variable Name(s)	Source (Year & Item # if Known)	ENGLISH Question & Response Options with Values			Item Conditions	Plat-form	Site-Specific Modifications
		CSF (Cerebral spinal fluid test) (cell count) सीएसएफ (सीरीब्रल स्पाइनल फ्लूड टेस्ट) (सेल काउंट)				All except PHC and SHC	
		Microbiology (blood, urine and CSF cultures) माइक्रोबायोलोजी (खून, पेशाब और सीएसएफ कल्चर)				All except SHC	
		Microalbumin and creatinine clearance test माइक्रोएल्ब्युमिन और क्रीएटीनाइन क्लीयरेंस टेस्ट				All except SHC	
		Troponin I test ट्रोपोनिन आइ टेस्ट				All except SHC	
		Troponin T test ट्रोपोनिन टी टेस्ट				All except SHC	
		Creatine Kinase test क्रिएटाइन किनेज टेस्ट				All except SHC	
		Creatine Kinase-MB test क्रिएटाइन किनेज - एमबी टेस्ट				All except SHC	
		Any BNP (B-type Natriuretic Peptide) test कोई बीएनपी (बी-टाइप नैथ्रीयूरेटिक पेप्टाइड) टेस्ट				All except SHC	
		Plasma glucose test (Fasting plasma glucose test)<br>प्लाज्मा ग्लूकोज टेस्ट (फास्टिंग प्लाज्मा ग्लूकोज टेस्ट)				All except SHC	
		HbA1c test (A1c test) एचबीए 1 सी परीक्षण				All except SHC	
		Fasting lipid profile फास्टिंग लिपिड प्रोफाइल				All except SHC	
		Total cholesterol test टोटल कोलेस्ट्राल टेस्ट				All except SHC	
		LDL cholesterol test एलडीएल कोलेक्स्ट्राल टेस्ट				All except SHC	
		Blood urea and serum creatinine test ब्लड यूरिया और सीरम क्रीएटिनाइन टेस्ट				All except SHC	
		Coagulation Test (PT/PTT/INR) कोगुलेशन टेस्ट (पीटी/पीटीटी/आईएनआर)				All except SHC	
TAB_INSTRUCT_SUPPLIES	IHME	**Assisted Observation: Medical consumables and supplies: with lab tech/ medical staff**<br>मदद से किया गया अवलोकन : चिकित्सा कंज्यूमेबल और आपूर्ति : लैब टेक्नीशियन/चिकित्सा कर्मचारी की मदद से<br>*Display as a table:*<br><br>Please report if the following medical consumables and supplies are available and functional at this facility today:<br>कृपया बतायें कि क्या आज इस केन्द्र पर निम्न चिकित्सा उपभोग्य सामग्रियाँ और आपूर्ति उपलब्ध हैं और कार्य कर रहे हैं:<br>Enter the following codes, if necessary for both the columns यदि आवश्यक हो तो दोनों कालमों में ये कोड लिखें<br>1=Yes हां<br>0=No नहीं<br>-1=Don't Know नहीं जानते<br>-2=Decline to respond जवाब देने से इंकार					
		**Equipment** उपकरण	**Available उपलब्ध है**	**Functional today आज कार्य कर रहे हैं**			None
MCS_AVAIL_TYP_; MCS_AVAIL_NOW_		Glucometer ग्लूको मीटर				All	
		Blood glucose test strips (for use with glucometer)<br>ब्लड ग्लूकोज टेस्ट स्ट्रिप (ग्लूकोमीटर के साथ इस्तेमाल के लिए)				All	
		Hematologic counter/analyzer<br>हीमेटालोजिक काउंटर/एनेलाइजर				All except SHC	
		Blood chemistry analyzer ब्लड केमिस्ट्री एनेजाइजर				All except SHC	

Unique Variable Name(s)	Source (Year & Item # if Known)	ENGLISH Question & Response Options with Values					Item Conditions	Plat-form	Site-Specific Modifications
		Centrifuge अपकेंद्रित्र						All except SHC	
		Microscope माइक्रोस्कोप						All except SHC	
WHO_ASSISTED	IHME	What were the positions of the respondents who helped you fill in this section of the survey?<br>उन उत्तरदाताओं का पद क्या था जिन्होंने सर्वेक्षण के इस खंड को भरने में आपकी मदद की?<br>(Blank Field)					required;		
		**Section 2: Pharmaceuticals**<br>**Please complete this section with a pharmacist or other individual who is well-informed about the pharmaceutical ordering and receipt system that is normally used in the pharmacy.**<br>**कृपया इस खंड को फार्मासिस्ट या किसी अन्य व्यक्ति कि सहायता से भरें जो कि दवाओं के आर्डर और प्राप्ति प्रक्रिया को अच्छी प्रकार से जानता हो. जिसका उपयोग सामान्यतः फार्मेसी में होता है**							
C_PHARM_AVAILABLE_DO		**Pharmaceutical Availability: Assisted Observation: with pharmacist**<br>फार्मास्युटिकल की उपलब्धता - फार्मासिस्ट की मदद से किया गया अवलोकन<br><br>Proceed to the pharmacy or place where pharmaceuticals are stored in this facility.<br>फार्मेसी या उस जगह जाएं जहाँ इस केन्द्र में दवाइयाँ स्टोर की जाती है.<br>For products listed, please indicate which are available today, and please report if the **drug was stocked out for 1 day in the last quarter** and **for more than 8 consecutive days in the last quarter.**<br>सूचीबद्ध उत्पादों के लिए, कृपया बतायें कि आज कौन-से उपलब्ध हैं, और कृपया बतायें कि क्या आखिरी तिमाही में 1 दिन के लिए और आखिरी तिमाही में लगातार 8 दिनों से अधिक समय तक के लिए दवा का स्टॉक खत्म हो गया था.<br><br>**Stock available(of any of the options within the category) today- आज की उपलब्धता**<br>Record if stock is available today- स्टॉक रजिस्टर से आज की उपलब्ध मात्रा लिखें<br><br>**Stocked out last quarter for one day**- as reported by pharmacist. पिछले तिमाही में एक दिन के लिए अनुपलब्ध- जैसा फार्मासिस्ट बताए वैसा लिखें<br><br>**Stocked out last quarter for more than 8 consecutive days**- as reported by pharmacist. पिछले तिमाही में लगातार आठ दिन से अधिक के लिए अनुपलब्ध- जैसा फार्मासिस्ट बताए वैसा लिखें<br>Response codes: जवाब के कोड<br>Enter -1 for Don't know नहीं जानते<br>Enter -2 for Decline to respond जवाब देने से इंकार<br>Enter -4 for Missing/Data not available गायब/जानकारी उपलब्ध नहीं					Available required, stocked out not required<br><br>INFORMS THE FORMULATIONS ASKED ABOUT LATER ON		site-specific
PHARM_AV_<br>AVAIL<br>SO1<br>SO8		**Drug Category**<br>दवा की कैटेगरी	**Drug Name**<br>दवा का नाम	**Stock available (of any of the options within the category) today**<br>**आज की उपलब्धता**	**Stocked out for at least 1 day IN THE LAST QUARTER** पिछले तिमाही में एक दिन के लिए अनुपलब्ध	**Stocked out for more than 8 consecutive days IN THE LAST QUARTER**<br>पिछले तिमाही में लगातार आठ दिन से अधिक के लिए अनुपलब्ध		All	None
		Biguanides<br>बाइग्यूनाइड्स	Acarbose आकरबोज<br>Miglitol मिग्लीटाल<br>Metformin मेटफार्मिन						

Unique Variable Name(s)	Source (Year & Item # if Known)	ENGLISH Question & Response Options with Values					Item Conditions	Plat-form	Site-Specific Modifications
		Insulin इंसुलिन	Regular Insulin रेगुलर इनसुलीन<br>NPH एनपीएच<br>Lente Insulin लेन्टी इनसुलीन						
		Sulphonylureas सल्फोनालूरीयाज	Glimepride ग्लाइप्राइड<br>Glipizide ग्लीपीजाइड<br>Glibenclamide ग्लीबेनक्लेमाइड						
		Thiazolidinediones थियाजोलिडिनेडाइयोन्स	Rosiglitazone रोसीग्लीटाजोन<br>Pioglitazoneपियोग्लीटाजोन						
		Diuretic डाइयूरेटिक	Furosemide (Frusemide) फ्यूरोसेमाइड (फ्रूसेमाइड)						
		Potassium-Sparing Diuretic पोटैशियम स्पारिंग डाइयूरेटिक	Spironolactone स्पिरोनोलैक्टोन						
		Statin स्टैटिन	Atorvastatin एटोरवैस्टेटिन<br>Simvastatin सिम्वैस्टेटिन<br>Rosuvastatin रोज़ूवैस्टैटिन						
		ACE Inhibitors ऐस इन्हिबिटर्स	Ramipril रैमिप्रिल<br>Enalapril इनालाप्रिल<br>Captopril कैप्टोप्रिल						
		Alpha agonist एल्फा एगोनिस्ट	Clonidine क्लोनिडाइन<br>Methyldopa मेथिलडोपा<br>Prazosin प्राजोसिन						
		Angiotensin Receptor Blockers एंगिओटेन्सिन रिसेप्टर ब्लाकर्स	Losartan लोसार्टन<br>Valsartan वैलसार्टन						
		Beta Blocker बीटा ब्लॉकर	Atenolol एटेनोलोल<br>Metoprolol मेटोप्रोलोल<br>Propranolol प्रोप्रानोलाल						
		Calcium Channel Blockers कैल्सियन चैनल ब्लाकर्स	Diltiazem tablets डिल्टिआजेम टैबलेट<br>Nifedipine (long-acting) नाइफेडिपाइन (लांग एक्टिंग)<br>Amlodipine आल्मोडिपाइन						
		Thiazide Diuretic थियेजाइड डाइयूरेटिक	Hydrochlorothiazide हाइड्रोक्लोरोथिएजाइड<br>Indapamide इन्डापैमाइड						
		Cardiac Glycoside	Digoxin डिगाक्सिन						

Unique Variable Name(s)	Source (Year & Item # if Known)	ENGLISH<br>Question & Response Options with Values					Item Conditions	Plat-form	Site-Specific Modifications
		कार्डिएक ग्लाइकोसाइड							
		Anticoagulant<br>एन्टीकोगुलेन्ट	Heparin हेपारिन<br>Fondaparinux फोन्डापैरिनक्स<br>Bivalirudin बिवालुरडीन						
		Antiplatelet<br>एन्टीप्लेटलेट	Ticagrelor टिकाग्रेलर<br>Clopidogrel क्लोपिडोगरेल						
		Nitrates<br>नाइट्रेट	Nitroglycerin (tablets)<br>नाइट्रोग्लीसरीन (टैबलेट)<br>Isosorbide dinitrate (Isordil)<br>इासोसारबाइड डाइनाइट्रेट (आइसोरडिल)						
		Opiates<br>ओपिएट्स	Morphine मोरफाइन						
		Salicylate<br>सैलिसिएट	Aspirin एस्पिरीन						
		Anticholinergics<br>एन्टीकोलिनेरजिक्स	Atropine Sulfate अल्ट्रोपाइन सल्फेट						
		Thrombolytic therapy<br>थ्रोम्बोलिटिक थिरेपी	Streptokinase or urokinase<br>स्ट्रेप्टोकिनेज या यूरोकिनेज						
		Antiarrhythmic drug<br>एण्टीएरिथमिक ड्रग	Lidocaine (Lignocaine hydrochloride) for Cardiac use<br>लिडोकैन (लिग्नोकैन हाइड्रोक्लोराइड) कार्डिएक इस्तेमाल के लिए						
		Beta-adrenergic agonist<br>बीटा एड्रेनेरजिक एगोनिस्ट	Epinephrine (Adrenaline)<br>एपीनेफ्राइन (एड्रेनालिन)						
WHO_ASSISTED	IHME	What were the positions of the respondents who helped you fill in this section of the survey?<br>उन उत्तरदाताओं का पद क्या था जिन्होंने सर्वेक्षण के इस खंड को भरने में आपकी मदद की?					required;	***ALL***	***None***
		**Section 3: Medical Consumables, Equipment and Facility Capacity**<br>Please complete this section with a nurse or other individual who is well-informed about the medical equipment, medical consumables and facility information that a nurse would normally know.<br>**कृपया इस खंड को नर्स या किसी अन्य व्यक्ति कि सहायता से भरें जो कि चिकित्सा उपकरणों , चिकित्सा उपभोग्य सामग्रियों और केंद्र की सूचना अच्छी प्रकार से जानता हो. जोकि नर्स आम तौर पर जानती है**							
SURGICAL		Does this facility provide surgery under regional or general anesthesia?<br>क्या यह केन्द्र स्थानीय या जनरल एनेस्थेसिअ के साथ सर्जरी की सुविधा प्रदान करता है?<br>(SELECT ONE)<br>☐ 1=Yes हां<br>☐ 0=No नहीं						ALL except PHC	None

Unique Variable Name(s)	Source (Year & Item # if Known)	ENGLISH<br>Question & Response Options with Values	Item Conditions	Plat-form	Site-Specific Modifications
		☐ -1=Don't Know नहीं जानते<br>☐ -2=Decline to respond जवाब देने से इंकार			
IMAGING		Does this facility provide any kind of diagnostic or imaging services (eg lab tests, XRAYS, CT Scan etc.)?<br>क्या यह केन्द्र किसी भी प्रकार की जांच कि सुविधा (नैदानिक या इमेजिंग सेवायें जैसे प्रयोगशाला परीक्षण, एक्सरे, सीटी स्कैन आदि) प्रदान करता है?<br>(SELECT ONE)<br>☐ 1=Yes हां<br>☐ 0=No नहीं<br>☐ -1=Don't Know नहीं जानते<br>☐ -2=Decline to respond जवाब देने से इंकार		ALL	None
WATER		Does this facility have running water for staff to wash their hands?<br>क्या इस केन्द्र में स्टाफ को अपने हाथ धोने के लिए नल का पानी उपलब्ध है?<br>(SELECT ONE)<br>☐ 1=Yes हां<br>☐ 0=No नहीं<br>☐ -1=Don't Know नहीं जानते<br>☐ -2=Decline to respond जवाब देने से इंकार<br>☐ -3=Not applicable नहीं लागू		ALL	None
IU_DECON	DHS SPA	What procedure is used for sterilising and cleaning equipment before its final processing for reuse?<br>(DO NOT READ ANSWERS ALOUD)<br>दुबारा इस्तेमाल करने के लिए अंतिम प्रोसेसिंग के पहले उपकरण को जीवाणुमुक्त करने और साफ करने के लिए कौन से तरीके इस्तेमाल किये जाते हैं?<br>जवाब को तेज आवाज में न पढें<br>(SELECT ALL THAT APPLY) (एक से अधिक उत्तर सम्भव)<br>☐ Use sterlisation equipment to clean कीटाणु रहित करने वाले उपकरणों के माध्यम से<br>☐ Soaked in disinfectant solution and then brush scrubbed with soap and water निस्संक्रामक घोल में भिगोना और फिर साबुन और पानी से ब्रश करना<br>☐ Brush scrubbed with soap and water and then soaked in disinfectant साबुन और पानी से ब्रश करना और फिर निस्संक्रामक घोल में भिगोना<br>☐ Brush scrubbed with soap and water only केवल साबुन और पानी से ब्रश करना<br>☐ Soaked in disinfectant, not brush scrubbed निस्संक्रामक घोल में भिगोना, ब्रश नहीं करना<br>☐ Cleaned with soap and water, not brush scrubbed साबुन और पानी से साफ करना, ब्रश नहीं करना<br>☐ Other अन्य<br>☐ No equipment is ever reused किसी भी उपकरण का कभी पुनः उपयोग नहीं हुआ<br>☐ No decontamination procedure for reused equipment पुनः उपयोग किये गए उपकरण के लिए परिशोधन प्रक्रिया नहीं<br>☐ Don't know नहीं जानते<br>☐ Decline to respond जवाब देने से इंकार<br>☐ Not applicable नहीं लागू	required; select all, excluding NONE/DK/DTR/NA;	ALL	None
		**Section 4: Assisted Observation with nurse/medical staff**<br>Please indicate the type and number of medical equipment items that are available at this facility today and of those how many are functional today. Functional denotes that particular equipment is in a condition to be used adequately and appropriately.			

Unique Variable Name(s)	Source (Year & Item # if Known)	ENGLISH Question & Response Options with Values				Item Conditions	Plat-form	Site-Specific Modifications
C_INPUT_MSE3		**Assisted Observation: Medical Equipment: with nurse/medical staff**<br>नर्स/ चिकित्सा कर्मचारी की मदद से किया गया अवलोकन - चिकित्सा उपकरण<br><br>In column 1, please report the total number of each item. In column 2 please report, of those available, how many are functional. In column 3, please report whether or not the item is staffed by someone at the facility who knows how to operate/use it.<br><br>कॉलम 1 में कुल उपलब्ध वस्तुओं की संख्या बतायें. कॉलम 2 में, कृपया बतायें कि उपलब्ध सामानों में से कितने कार्य कर रहे हैं. कॉलम 3 में, कृपया बतायें कि क्या इन उपकरणों को संचालित करने के लिए इस केंद्र में कोई कर्मचारी नियुक्त है जो इसको संचालित करना जनता है.				Show if #IMAGING==1<br>Page Break		
		**Item** आइटम	**Column 1: Total Available TODAY**<br>आज उपलब्ध कुल संख्या<br>-1=Don't know नहीं जानते<br>-2=Decline to respond जवाब से इंकार<br>-3=Not applicable नहीं लागू	**Column 2: Total Functional** कुल क्रियाशील<br>Enter the following codes:<br>0=None functional at this facility<br>इस केन्द्र में कोई भी काम नहीं करता<br>-1=Don't know नहीं जानते<br>-2=Decline to respond जवाब से इंकार<br>-3=Not applicable नहीं लागू	**Column 3:** Is there someone working in this facility that knows how to use the equipment?<br>क्या इस केन्द्र में काम करने वाला ऐसा कोई व्यक्ति है जो जानता है कि तरह से उपकरण को इस्तेमाल किया जाय?<br>1=Staffed by someone at the facility who knows how to operate/use it केंद्र में कर्मचारी मौजूद हैं जो संचालित करना जानता है<br>0=Not staffed by someone at the facility who knows how to operate/use it केंद्र में कर्मचारी मौजूद नहीं हैं जो संचालित करना जानता है<br>1=Staffed by someone at the facility who knows how to operate/use it केंद्र में कर्मचारी मौजूद हैं जो संचालित करना जानता है<br>0=Not staffed by someone at the facility who knows how to operate/use it केंद्र में कर्मचारी मौजूद नहीं हैं जो संचालित करना जानता है<br>-1=Don't know नहीं जानते<br>-2=Decline to respond जवाब से इंकार<br>-3=Not applicable नहीं लागू			None
		Adult Scale युवा स्केल					All	
		Child Scale (weight graduation min 250 grams) शिशु स्केल (न्यूनतम वजन 250 ग्राम)					All	
		Blood Pressure Apparatus (digital or manual with cuff)<br>ब्लड प्रेशर उपकरण (डिजिटल या कफ वाले मैनुअल)					All	
		Stethoscope स्टेथोस्कोप					All	

Unique Variable Name(s)	Source (Year & Item # if Known)	ENGLISH<br>Question & Response Options with Values				Item Conditions	Plat-form	Site-Specific Modifications
		Cardiac Monitor कार्डिएक मानीटर					All	
		Pulse Oximeter पल्स आक्सीमीटर					All	
		Xray machine एक्सरे मशीन					All except PHC	
		Electrocardiography (ECG or EKG)<br>इलेक्ट्रोकार्डियोग्रैफी (ईसीजी या ईकेजी)					All except PHC	
		Treadmill stress ECG<br>ट्रेडमिल स्ट्रेस ईसीजी					All except PHC	
		Ultrasound for body imaging<br>बाडी इमेजिंग के लिए अल्ट्रासाउण्ड					All except PHC	
		Dedicated cardiac ultrasound (echocardiogram machine) with Doppler<br>डाप्लर वाले समर्पित कार्डिएक अल्ट्रासाउण्ड (इकोकार्डियोग्राम मशीन)					All except PHC	
		Stress echocardiography with exercise or pharmacologic stress<br>एक्सरसाइज या फार्माकोलोजिक स्ट्रेस वाला स्ट्रेस इकोकार्डियोग्रैफी					All except PHC	
		CT scan for body imaging<br>बाडी इमेजिंग के लिए सीटी स्केल					All except PHC	
		Cardiac CT with dedicated coronary imaging<br>समर्पित कोरोनेयरी इमेजिग वाला कार्डिएक सीटी					All except PHC	
		Electron Beam CT for coronary calcium scoring<br>कोरोनेयरी कैल्शियम स्कोरिंग के लिए इलेक्ट्रान बीम सीटी					All except PHC	
		SPECT (single photon emission tomography) nuclear scanner<br>स्पेक्ट (सिंगल फोटान एमिशन टोमोग्रैफी) न्यूक्लीयर सकैनर					All except PHC	
		Myocardial perfusion imaging (cardiac SPECT with exercise or pharmacologic stress)<br>मायोकार्डियल परफ्यूजन इमेजिंग (एक्सरसाइज या फार्माकोलोजिक स्ट्रेस वाला कार्डिएक स्पेक्ट)					All except PHC	
		Defibrillator<br>डेफिब्रिलेटर					All	
		Automatic External Defibrillator (AED)<br>आटोमैटिक एक्टरनल डेफिब्रिलेटर						
		Dialysis machine डायलसिस मशीन						
		Ventilator (Adult) वेंटिलेटर (युवा)					All	

Unique Variable Name(s)	Source (Year & Item # if Known)	ENGLISH Question & Response Options with Values		Item Conditions	Plat-form	Site-Specific Modifications
C_INPUT_MSE4		**Assisted Observation: Medical Supplies: with nurse/medical staff**<br>मदद से किया गया अवलोकन - चिकित्सा आपूर्ति - नर्स/चिकित्सा कर्मचारी की मदद से<br><br>Please report on the availability of each medical supply item today.<br>कृपया किसी सामान्य दिन और आज के दिन प्रत्येक मेडिकल आपूर्ति मद की उपलब्धता दर्ज करें.<br><br>1=Available उपलब्ध<br>0=Not available उपलब्ध नहीं<br>-1=Don't know नहीं जानते<br>-2=Decline to respond जवाब से इंकार		Page Break		
		**Item** आइटम	**Available today?** आज उपलब्ध है?			
		Gloves दस्ताने				
		Equipment Disinfectant उपकरण को जीवाणुविहीन करने वाला				
		Hand disinfectant हाथ को जीवाणुविहीन करने वाला				
		Needle holder नीडल होल्डर				
		Eye protection आंख की सुरक्षा				
C_INSTR_INPUT_PERS		**Assisted Observation: Staff Inventory: with nurse/medical staff**<br>मदद से किया गया अवलोकन : स्टाफ इंवेट्री : नर्स/चिकित्सा कर्मचारी की मदद से<br>*Please inventory the staffs who are working at the facility the day that you are completing this module. Ask the individual assisting you during this module to inform you who is working that day.*<br>*जिस दिन आप इस मॉड्यूल को पूरा कर रहे हैं उस दिन इस केन्द्र पर काम कर रहे स्टाफ की कृपया सूची बनायें. इस मॉड्यूल को पूरा करने के दौरान आप की सहायता कर रहे लोगों से पूछें कि उस दिन कौन काम कर रहा है.*<br>Enter the following codes, if necessary: आवश्यक होने पर ये कोड लिखें<br>0=None कोई नहीं<br>-1=Don't know नहीं जानते<br>-2=Decline to respond जवाब से इंकार			Only for SC, PHC and CHC in public sector	None
		**Total staff कुल कर्मचारी**	**Number working today आज कार्य कर रहे लोगों की संख्या**			
tot_staff		(a) Doctors<br>डाकटर				
		(b) Nurses excluding ANM<br>नर्स, एएनएम को छोडकर				
		(c) ANM/Village Health Nurse एएनएम/ ग्राम स्वास्थ्य नर्स				
		(d) Matron मैट्रन				
		(e) Ward Nurse वार्ड नर्स				
		(f) Ward Sister वार्ड सिस्टर				
		(g) Female Healthworkers महिला स्वास्थ्यकार्यकर्ता				

Unique Variable Name(s)	Source (Year & Item # if Known)	ENGLISH Question & Response Options with Values		Item Conditions	Plat-form	Site-Specific Modifications
		(h) Traditional Birth Attendants (TBAs) दाई				
		(i) Other paramedical staff (excluding nurses and ANM)<br>(j) अन्य पैरामेडिकल कर्मचारी (नर्स और एएनएम को छोडकर)				
		(k) Non-medical staff<br>गैर चिकित्सा कर्मचारी				
		**Treatment Pathways Provided**<br>दिये गये इलाज का ब्यौरा<br>Please answer the following questions with a staff member who is well informed of the procedures performed at this health facility, such as a doctor.<br>कृपया आगे के सवालों के जवाब किसी ऐसे कर्मचारी की मदद से दें जो इस केन्द्र पर किये जाने वाले प्रोसीजर को अच्छी तरह से जानता हो जैसे कोई डाक्टर<br>Are the following Treatment Pathways provided at this facility?<br>क्या इस केन्द्र में नीचे दिये गये इलाज दिये जाते हैं?<br>1= yes हां<br>0= no नहीं<br>-1=Don't know नहीं जानते<br>-2=Decline to respond जवाब से इंकार			All except for PHC and SHC	None
			**Is this treatment pathway provided?** क्या यह इलाज दिया जाता है?			
		Reperfusion therapy for acute myocardial infarction with intravenous thrombolytic medication (inpatient)<br>इन्ट्रावीनस थ्राम्बायोटिक दवाओं (भर्ती मरोज) के साथ गंभीर मायोकार्डियल इन्फ्रैक्शन के लिए रीपरफ्यूजन थिरेपी				
		Outpatient medical management, including oral medical therapy, for heart failure (outpatient)<br>दिल के मरीजों के लिए बाह्यरोगी चिकित्सा प्रबन्ध जिसमें ओरल मेडिकल थिरेपी शामिल है				
		Acute and immediate care of undifferentiated/undiagnosed chest pain (outpatient), including an ECG<br>एक जैसे/ बिना जांच वाले सीने के दर्द (बाह्यरोगी) जिसमें ईसीजी शामिल है उनके लिए तीव्र और तुरन्त देखभाल				
		Outpatient therapy for ischemic heart disease using oral medical therapy<br>आइशेमिक हृदय रोग के लिए ओरल मेडिकल थिरेपी इस्तेमाल करके आउटपेशेंट थिरेपी				
		Treatment for diabetes using oral medical therapy and insulin<br>ओरल मेडिकल थिरेपी और इन्सुलीन इस्तेमाल करके डाइबिटीज का इलाज				
		Treatment for chronic kidney disease not necessarily including hemodialysis<br>पुराने किडनी रोगों का इलाज जिसमें कोई जरूरी नहीं कि हीमोडायलिसिस शामिल हो				
		Acute and immediate for undiagnosed neurological conditions and suspected stroke<br>बिना जांच वाले न्यूरोलोजिकल और संभावित स्ट्रोक के लिए तीव और तुरन्त				
		Treatment for diagnosed stroke including oral medical therapy				

Unique Variable Name(s)	Source (Year & Item # if Known)	ENGLISH Question & Response Options with Values	Item Conditions	Plat-form	Site-Specific Modifications
		जांच वाले स्ट्रोक के लिए जिसमें ओरल मेडिकल थिरेपी शामिल है, के लिए इलाज			
		Cardiac catheterization lab for diagnostic coronary angiography<br>डाइग्नोस्टिक कोरोनेयरी एंजिओग्रैफी के लिए कार्डिएक कैथेटराइजेशन			
		Outpatient Cardiac Rehabilitation services with exercise training<br>व्यायाम प्रशिक्षण के साथ आउटपेशेंट कार्डिएक रिहैबिलिटेशन सेवाएं			
		Revascularization with percutaneous coronary intervention and coronary stentin<br>पर्कुटेनियस कोरोनेयरी इंटरवेंशन और कोरोनेयरी स्टेंटिन के साथ रीवैस्क्युलेराइजेशन			
		Revascularization with coronary artery bypass grafting surgery<br>कोरोनेयरी आर्टरी बाइपास ग्रैपिफ्टंग सर्जरी के साथ रीवैस्क्युलेराइजेशन			
		**Section 6: Facility Characteristics**			
		This section is designed for direct observation of some this facility's characteristics. This section is to be completed by the interviewer only, and does not need input from any facility staff..<br><br>यह अनुभाग इस केन्द्र की कुछ विशेषताओं के प्रत्यक्ष निरीक्षण के लिए बनाया गया है. इस अनुभाग को साक्षात्कारकर्ता द्वारा ही पूरा किया जाना है, और किसी भी केन्द्र के कर्मचारियों से जानकारी लेने की जरूरत नहीं है. सभी प्रत्यक्ष प्रेक्षण निर्देश पीले रंग में रखे गए हैं.			
GPS_DEGREES<br>GPS_MIN<br>GPS_SEC		**Facility GPS Coordinates:** केन्द्र के जीपीएस कोआर्डिनेट<br>Pleases enter in the same format as displayed on your GPS Device. कृपया जीपीएस उपकरण पर दर्शाये गये फार्मेट को उसी तरह से भरें।<br>Enter the latitude and longitude लैटीट्यूट और लांगीट्यूड (अक्षांस और देशान्तर) भरें<br><br>Enter the following codes, if necessary: आवश्यक होने पर ये कोड लिखें<br>-1= Error with GPS Device जीपीएस उपकरण में खराबी			
		Enter degrees कोण लिखें / Enter minutes मिनट लिखें / Enter seconds सेकेण्ड लिखें			
		Latitude लैटीट्यूट			
		Longitude लांगीट्यूड			
FAC_ELEV		**Altitude in meters:** ______________________________<br>मीटर में ऊंचाई			
RURAL	IHME	Is this facility located in a rural or urban area?<br>क्या यह केन्द्र ग्रामीण या शहरी क्षेत्र में स्थित है?<br>(If you are not sure, ask the facility administrator)<br>यदि आप इस बारे में सुनिश्चित न हों तो केन्द्र के प्रशासक से पूछें<br>(SELECT ONE)<br><br>☐ 1=Rural ग्रामीण<br>☐ 2=Semi-/Peri-urban अर्द्ध ग्रामीण<br>☐ 3=Urban शहरी	required;	ALL	None
FAC_PERM		What type of structure is the main building of this facility?<br>इस केन्द्र के मुख्य भवन का ढांचा किस प्रकार का है?<br>(SELECT ONE)<br>☐ 0=Permanent स्थायी	required;	ALL	Country specific

Unique Variable Name(s)	Source (Year & Item # if Known)	ENGLISH<br>Question & Response Options with Values	Item Conditions	Plat-form	Site-Specific Modifications
		☐ 1 =semi-permanent अर्द्ध अस्थायी<br>☐ 2= Temporary अस्थायी<br>☐ -4=Missing data/not observed जानकारी नहीं है/ अवलोकन नहीं किया			
FAC_BUILDINGS		Please indicate the number of buildings on this campus. Do not include sheds and parking areas in this count<br>कृपया इस परिसर में भवनों की संख्या बतायें. इस गिनती में शेड और पार्किंग क्षेत्र शामिल न करें<br>(SELECT ONE)<br>☐ 1 =Number of buildings ______ भवानों की संख्या<br>☐ -1=Don't know नहीं जानते<br>☐ -2=Decline to respond जवाब से इंकार<br>☐ -3=Not applicable लागू नहीं		ALL	None
FAC_ROOMWAIT_DO	IHME	Does this facility have designated waiting area (s) available that is separated from the examination room(s)?<br>क्या इस केंद्र में परीक्षण कक्ष से अलग एक निर्दिष्ट प्रतीक्षा कक्ष उपलब्ध है?<br>(SELECT ONE)<br>☐ 3=Yes, observed at least one designated waiting area separated from the examination rooms हां, परीक्षण कमरे से अलग कम से कम एक निर्दिष्ट प्रतीक्षा क्षेत्र देखा<br>☐ 0=No, waiting areas(s) not separated from the examination rooms/area नहीं, प्रतीक्षा क्षेत्र परीक्षण कमरे / क्षेत्र से अलग नहीं है<br>☐ -3=Not applicable, no designated waiting **area** लागू नहीं, कोई नियत प्रतीक्षा क्षेत्र नहीं<br>☐ -4=Missing data/not observed जानकारी नहीं है/ अवलोकन नहीं किया	required;	ALL	Country specific
FAC_ROOMWAITOUT_DO		If outdoors, is the designated waiting area protected from the elements- is shade or rain-covering provided? Consider all waiting areas if more than one.<br>यदि बाहर हो,तो क्या निर्दिष्ट प्रतीक्षा क्षेत्र छायादार और बारिश से सुरक्षित है या बारिश कवरिंग प्रदान की गई है? एक से अधिक प्रतीक्षा क्षेत्र हो तो सभी क्षेत्रों पर विचार करें.<br>(SELECT ONE)<br>☐ 2 = Yes, well protected हां, अच्छी तरह सुरक्षित<br>☐ 1 = No, not well protected नहीं, अच्छी तरह सुरक्षित नहीं<br>☐ 0= Waiting area is not outside प्रतीक्षा क्षेत्र बाहर नहीं है<br>☐ -3=Not applicable, no waiting area नहीं लागू, प्रतीक्षा क्षेत्र नहीं<br>☐ -4=Missing data/not observed जानकारी नहीं है/ अवलोकन नहीं किया		ALL	None
FAC_ROOMWAIT_CLEANLINESS_DO	IHME	In general, describe the overall cleanliness of the waiting areas: Consider all waiting areas if more than one.<br>सामान्य रूप से, प्रतीक्षा क्षेत्रों कि पूरी साफ सफाई का वर्णन करें: एक से अधिक प्रतीक्षा क्षेत्र हो तो सभी क्षेत्रों पर विचार करें.<br>☐ (2=Clean and orderly in appearance दिखने में साफ और व्यवस्थित<br>☐ 1=Somewhat dirty in appearance, with minor spills, debris, or trash present दिखने में थोड़ा गंदा, कुछ बिखराव, मलबा या कचरा<br>☐ 0=Very dirty in appearance, with major spills, debris, or trash on the chairs or floor दिखने में काफी गंदा, कुर्सियों और फर्श पर मलबा या कचरा का बिखराव<br>☐ -3=Not applicable, no waiting areas नहीं लागू, प्रतीक्षा क्षेत्र नहीं<br>☐ -4=Missing data/not observed जानकारी नहीं है/ अवलोकन नहीं किया	required; show if FAC_ROOMWAIT_DO=3|2|1	ALL	None
FAC_EXAMROOM_CLEANLINESS_DO		In general, describe the overall cleanliness of the examination room(s): Consider all examination areas if more than one.<br>सामान्य रूप से, परीक्षण कक्ष कि पूरी साफ सफाई का वर्णन करें: एक से अधिक परीक्षण कक्ष हो तो सभी क्षेत्रों पर विचार करें.<br>(SELECT ONE)<br>☐ 2=Clean and orderly in appearance दिखने में साफ और व्यवस्थित<br>☐ 1=Somewhat dirty in appearance, with minor spills, debris, or trash present दिखने में थोड़ा गंदा, कुछ बिखराव, मलबा या कचरा	required;	ALL	None

Unique Variable Name(s)	Source (Year & Item # if Known)	ENGLISH<br>Question & Response Options with Values	Item Conditions	Plat-form	Site-Specific Modifications
		☐ 0=Very dirty in appearance, with major spills, debris, or trash on the chairs or floor दिखने में काफी गंदा, कुर्सियों और फर्श पर मलबा या कचरा का बिखराव<br>☐ -3=Not applicable, no waiting areas नहीं लागू, प्रतीक्षा क्षेत्र नहीं<br>☐ -4=Missing data/not observed जानकारी नहीं है/ अवलोकन नहीं किया			
EXAM_DO	IHME<br>SPA 322	In general, describe the overall setting of the examination room(s): Consider all examination areas if more than one.<br>सामान्य रूप से, परीक्षण कक्ष के समग्र सेटिंग का वर्णन करें: एक से अधिक परीक्षण कक्ष हो तो सभी क्षेत्रों पर विचार करें.<br>(SELECT ONE)<br>☐ 3= Room with visual and auditory privacy दृश्य और श्रवण गोपनीयता वाला कमरा<br>☐ 2= Room with visual privacy only केवल दृश्य गोपनीयता वाला कमरा<br>☐ 1= Room with auditory privacy only केवल श्रवण गोपनीयता वाला कमरा<br>☐ 0=No privacy कोई गोपनीयता नहीं<br>☐ -4=Missing data/not observed	required; show if FAC_EXAMROOM_CLEANLINESS_DO=2|1|0|-4	ALL	None
FEELIST_DO	IHME	Is there an official fee list posted so that the patients can easily see it?<br>क्या एक अधिकृत शुल्क सूची लगी है ताकि मरीज़ उसे आसानी से देख सकें?<br>(SELECT ONE)<br>☐ 1=Yes हां<br>☐ 0=No नहीं<br>☐ -3=Not applicable, facility does not collect fees from patients नहीं लागू, केन्द्र मरीजों से शुल्क नहीं लेता<br>☐ -4=Missing data/not observed जानकारी नहीं है/ अवलोकन नहीं किया	required; show if FEES=1	ALL	Country specific
C_GUIDELINES_DO	IHME<br>DHS SPA GENERAL INFO question 1415	**Guideline Observation**<br>मार्गदर्शन अवलोकन<br>*Display as a table:*<br>Indicate whether or not the following guidelines are present. A guideline can be a document/poster etc., they do not need to be posted, but should be available to medical staff (for example: on a computer or in a folder readily available at this facility). If you do not directly observe them, inquire with a medical staff if there are any of the following guidelines available.<br>सूचित कीजिए कि निम्नलिखित दिशानिर्देश मौजूद हैं या नहीं. एक दिशानिर्देश एक दस्तावेज़ / पोस्टर आदि हो सकता है, यह ज़रूरी नहीं है कि वो कहीं चिपका हो, लेकिन मेडिकल स्टाफ के लिए उपलब्ध होना चाहिए (उदाहरण के लिए : किसी कंप्यूटर या किसी फोल्डर में जो इस केन्द्र में आसानी से उपलब्ध हो) . यदि आपको वे सीधे दिखाई नहीं पड़ते हैं तो चिकित्सा स्टाफ से पूछताछ करें कि क्या निम्नलिखित में से कोई दिशानिर्देश उपलब्ध हैं.<br>(SELECT ONE)<br>☐ 1=Yes, observed हां, अवलोकन किया<br>☐ 2=Yes available but not observed हां, उपलब्ध है लेकिन अवलोकन नहीं किया<br>☐ 0=No guidelines present कोई मार्गदर्शन उपलब्ध नहीं<br>☐ -3=Not applicable नहीं लागू<br>☐ -4=Missing data/not observed जानकारी नहीं है/ अवलोकन नहीं किया	required;		Based on National MOH website
GUIDELINES_DO_1	"	Disease specific treatment guidelines: Diabetes किसी रोग के इलाज के बारे में मार्गदर्शन - डाइबिटीज		ALL	
2		Disease specific treatment guidelines: Hypertension किसी रोग के इलाज के बारे में मार्गदर्शन - उच्च रक्त चाप		ALL	
3		Disease specific treatment guidelines: Ischaemic Heart Disease and Acute Myocardial Infarction किसी रोग के इलाज के बारे में मार्गदर्शन - आइशेमिक हार्ट डिजीज और एक्यूट मायोकार्डियल इन्फ्रैक्शन		ALL	

Unique Variable Name(s)	Source (Year & Item # if Known)	ENGLISH<br>Question & Response Options with Values	Item Conditions	Plat-form	Site-Specific Modifications
4		Disease specific treatment guidelines: Management of Critical Care किसी रोग के इलाज के बारे में मार्गदर्शन - गंभीर देखभाल प्रबन्ध		ALL	
5		Disease specific treatment guidelines: Management of Cardiovascualr Diseases किसी रोग के इलाज के बारे में मार्गदर्शन - हृदय रोगों से बचाव		ALL	
6		Disease specific treatment guidelines: Endocrinology किसी रोग के इलाज के बारे में मार्गदर्शन - एण्डोक्रिनोलोजी		ALL	
		Disease specific treatment guidelines: Neurology किसी रोग के इलाज के बारे में मार्गदर्शन - न्यूरोलोजी		ALL	
7		National Programme for Prevention and Control of Cancer, Diabetes, Cardiovascular Diseases &Stroke (NPCDCS) Heatlh Worker Training Guideline<br><br>कैंसर, डाइबिटीज, हृदय रोग, आघात के रोकथाम और नियंत्रण के राष्ट्रीय कार्यक्रम (एनपीसीडीसीएस) के स्वास्थ्यकर्मी के प्रशिक्षण संबंधी मार्गदर्शन		ALL	
8		National Programme for Prevention and Control of Cancer, Diabetes, Cardiovascular Diseases &Stroke (NPCDCS) Operational Guidelines<br><br>कैंसर, डाइबिटीज, हृदय रोग, आघात के रोकथाम और नियंत्रण के राष्ट्रीय कार्यक्रम (एनपीसीडीसीएस) के कार्यसंचालन संबंधी मार्गदर्शन		ALL	
IU_TOILETS;<br>FLUSH_SEWER<br>FLUSH_TANK<br>FLUSH_PIT<br>FLUSH_ELSEWHERE<br>FLUSH_UNKNOWN<br>TRAD_PIT<br>VENT_PIT<br>COVERED_PIT<br>UNCOVERED_PIT<br>HANGING<br>BUCKET<br>NONE<br>OTHER<br>DK<br>DTR<br>NA	IHME<br>DHS SPA<br>GC13<br>census	What types of toilet facilities are available and functional to patients and staff?<br>मरीजों और स्टाफ के लिए किस प्रकार की शौचालय सुविधा उपलब्ध हैं और कार्य कर रहे हैं?<br>(SELECT ALL THAT APPLY) (एक से अधिक उत्तर सम्भव)<br><br>☐ Flush/pour flush to piped sewer system फ्लश/ पानी डालकर फ्लश करने वाला पाइप वाला सीवर सिस्टम<br>☐ Flush/pour flush to septic tank फ्लश/ सेप्टिक टैंक में फ्लश किया जाना<br>☐ Flush/pour flush to pit latrine फ्लश/ पानी डालर फ्लश की जाने वाली पिट लैट्रिन<br>☐ Flush/pour flush to elsewhere फ्लश/ पानी डालकर कहीं और फ्लश करना<br>☐ Flush/pour flush to unknown destination फ्लश/ पानी डालकर किसी अनजान जगह पर फ्लश करना<br>☐ Covered, ventilated pit latrine ढका, हवादार पिट लैट्रिन<br>☐ Covered, unventilated pit latrine ढका, बगैर हवादार पिट लैट्रिन<br>☐ Uncovered pit latrine बिना ढकी पिट लैट्रिन<br>☐ Bucket बाल्टी<br>☐ No toilet facilities are available and functional at this facility इस केन्द्र पर कोई चालू शौचालय सुविधा उपलब्ध नहीं<br>☐ Other अन्य<br>☐ -1=Don't know नहीं जानते<br>☐ -2=Decline to respond जवाब से इंकार		ALL	None
WHO_ASSISTED	IHME	What were the positions of the respondents who helped you fill in this section of the survey?<br>उन उत्तरदाताओं का पद क्या था? जिन्होंने सर्वेक्षण के इस खंड को भरने में आपकी मदद की?<br>(SELECT ALL THAT APPLY) (एक से अधिक उत्तर सम्भव)<br>☐ 1 =Facility Administrator केन्द्र प्रशासक<br>☐ 2 = Doctor डाक्टर<br>☐ 3 = Nurse नर्स<br>☐ 4 = Pharmacist फार्मासिस्ट<br>☐ 5= Laboratory Technition लैब टेक्नीशियन<br>☐ 6= Accountant एकाउंटेन्ट<br>☐ 7= Human Resource Manager मानव संसाधन प्रबन्धक<br>☐ 8= HMIS Manager एचएमआईएस प्रबन्धक	required;	All	None

Unique Variable Name(s)	Source (Year & Item # if Known)	ENGLISH Question & Response Options with Values	Item Conditions	Plat-form	Site-Specific Modifications
		☐ 9= Clerk क्लर्क<br>☐ 10 = Other Staff, Specify अन्य कर्मचारी, स्पष्ट करें			
COMMENTS2	IHME	Please use this space to provide us with any feedback/comments/issues you encountered while completing this section.<br><br>इस खंड को पूरा करने में आपका सामना जिस भी प्रतिक्रिया / टिप्पणी / मुद्दों के साथ हुआ है उसे हमें प्रदान करने के लिए नीचे दिए गए स्थान का प्रयोग करें.			
		Thank you for participating in the Facility Survey Module 1: Inputs, Finances and Management<br>केन्द्र के सर्वे माडयूल 1 : जानकारी, वित्त और प्रबन्धन में भाग लेने के लिए आपको धन्यवाद<br>Click 'Get Time' button. 'गेट टाइम' बटन पर क्लिक करें			

Unique Variable Name(s)	Source (Year & Item # if Known)	ENGLISH Question & Response Options with Values	Item Conditions	Platform	Site-Specific Modifications
		**Module 3: Outputs**			
	IHME	**Survey Log-In**			
FAC_DISTRICT		Enter District ID:<br>जिले की पहचान संख्या लिखें			
CUSTOMID	IHME	Enter the CUSTOMID for this facility:________<br>इस केन्द्र का कस्टमआईडी प्रविष्ट करें:________	required;	ALL	None
FIELDSTAFF_ID<br>FIELDSTAFF_ID_TEXT	IHME	Enter your Interviewer ID: ______________<br>अपना साक्षात्कारकर्ताआईडी प्रविष्ट करें: ______________	required;<br>text only;<br>page break	ALL	None
platform_type	IHME	Health service delivery platform type:<br>स्वास्थ्य सेवा प्रदान करने वाले प्लेटफॉर्म का प्रकार:<br>☐ 1= District Hospital (DH) जिला अस्पताल (डीएच)<br>☐ 2 = Civil Hospital (CH) सिविल अस्पताल (सीएच)<br>☐ 3 = Community Health Centre (CHC) सामुदायिक स्वास्थ्य केन्द्र (सीएचसी)<br>☐ 4 = Primary Health Centre (PHC) प्राथमिक स्वास्थ्य केन्द्र (पीएचसी)<br>☐ 5 = Sub Health Centre (SHC) उपस्वास्थ्य केन्द्र (एसएचसी)<br>☐ 6 = Private Hospital प्राइवेट अस्पताल<br>☐ 7 = Private Clinic प्राइवेट क्लीनिक	PRELOAD;	ALL	site-specific platform types (with follow-up questions to define facility type as needed)
fac_private		Is this a private facility? क्या यह प्राइवेट स्वास्थ्य केन्द्र है?<br>☐ Yes हां<br>☐ No नहीं			

Unique Variable Name(s)	Source (Year & Item # if Known)	ENGLISH<br>Question & Response Options with Values	Item Conditions	Platform	Site-Specific Modifications
fac_years	IHME	How many years has this facility been in existence?<br>यह केन्द्र कितने दिनों से अस्तित्व में है?<br>(SELECT ONE)<br>☐ 1=ENTER NUMBER OF YEARS: _____ वर्षों की संख्या लिखें<br>(If the administrator is unaware, please enter their best estimate.) यदि व्यवस्थापक अनजान है तो कृपया उनके सर्वश्रेष्ठ अनुमान को भरें	required;<br>select one;<br>range 0-200;	ALL	none
INPATIENT		Does this facility provide inpatient care?<br>क्या यह केंद्र अंतःरोगी सेवा प्रदान करता है?<br>(SELECT ONE)<br>☐ 1=Yes हां<br>☐ 0=No नहीं<br>☐ -1=Don't Know नहीं जानते<br>☐ -2=Decline to respond जवाब देने से इंकार	current.platform_type<>5	All except SHC	None
FAC_MOBILE	IHME	Does this facility provide mobile clinic services?<br>क्या यह केन्द्र मोबाईल क्लिनिक सेवा प्रदान करता है?<br>(SELECT ONE)<br>☐ 1=Yes हां<br>☐ 0=No नहीं<br>☐ -1=Don't Know नहीं जानते<br>☐ -2=Decline to respond जवाब देने से इंकार	current.platform_type<>5	All except SHC	Site-specific
CATCH	CHAI Sur. 1	What is the size of the catchment population for this facility?<br>यह केंद्र जिस जनसँख्या को सेवा प्रदान करता है उसका आकार क्या है?<br>(SELECT ONE)<br>☐ 1= Size: _____ आकार<br>☐ -1=Don't Know नहीं जानते<br>☐ -2=Decline to respond जवाब देने से इंकार		All	none
EC_REFERRAL	IHME DHS RWANDA SPA Question 449	Does this facility ever refer patients to another facility for emergency care?<br>आपातकालीन देखभाल के लिए क्या यह केन्द्र रोगियों को कभी भी दूसरे केन्द्र पर भेजता है?<br>(SELECT ONE)<br>☐ 1=Yes हां<br>☐ 0=No नहीं<br>☐ -1=Don't Know नहीं जानते<br>☐ -2=Decline to respond जवाब देने से इंकार	required;	All	none
fiscal_yr		Are the start and end dates of this facility's most recent fiscal year April 2013 - March 2014?<br>क्या इस केन्द्र के शुरुआत और अंत की तारीखें वित्तीय वर्ष अप्रैल 2013 - मार्च 2014 में बिलकुल हाल ही की हैं?<br>☐ 1=Yes हां<br>☐ 0=No नहीं			

Unique Variable Name(s)	Source (Year & Item # if Known)	ENGLISH<br>Question & Response Options with Values	Item Conditions	Platform	Site-Specific Modifications
fiscal_yr2_month<br>fiscal_yr2_year		What is the first month of the most recently completed fiscal year?<br>बिलकुल हाल ही में पूरा किये गये वित्त वर्ष का पहला महीना कौन सा है?<br>Month महीना:_______________(January, February, March, April, May, June, July, August, September, October, November, December)<br>(जनवरी, फरवरी, मार्च, अप्रैल, मई, जून, जुलाई, अगस्त, सितम्बर, अक्तूबर, नवम्बर, दिसम्बर<br>Year वर्ष:_______________(2012, 2013)	current.fiscal_yr = 0		
		**General Outpatient Questions** सामान्य बाह्य रोगी प्रश्न<br>For this section, please answer the following questions with a facility staff member that is familiar with medical records regarding outpatient visits.<br>इस सेक्शन के लिए कृपया केन्द्र के किसी कर्मचारी की मदद से इनका जवाब दें जो बाह्य रोगी विजिट के बारे में चिकित्सा रिकार्ड से परिचित हो			
PED_AGE	IHME	What age group is considered pediatric patients at this facility?<br>इस केन्द्र में किस आयु वर्ग को बाल चिकित्सा रोगी माना जाता है?<br>(SELECT ONE)<br>☐ 5= 0-12 years 0-12 वर्ष<br>☐ 4= 0-14 years 0-14 वर्ष<br>☐ 3= 0-15 years 0-15 वर्ष<br>☐ 2= 0-16 years 0-16 वर्ष<br>☐ 1= 0-19 years 0-19 वर्ष<br>☐ 0= Other (specify) अन्य, स्पष्ट करें<br>☐ -1=Don't Know नहीं जानते<br>☐ -2=Decline to respond जवाब देने से इंकार<br>☐ -3=Not applicable नहीं लागू		ALL	None
PED_AGE_OSP		Please specify 'other': ___________________________<br>कृपया 'अन्य' को स्पष्ट करें			
OPC_REC		Are outpatient visits recorded at this facility? क्या इस केन्द्र पर बाह्यरोगियों के आने को रिकार्ड किया जाता है?<br>☐ 1=Yes हां<br>☐ 0=No नहीं<br>☐ -1=Don't Know नहीं जानते<br>☐ -2=Decline to respond जवाब देने से इंकार<br>☐ -3=Not applicable नहीं लागू			
OPC_MONTH_YEAR		Are outpatient visits recorded by month or by year?<br>क्या बाह्यरोगियों के आने को महीने या साल के रूप में रिकार्ड किया जाता है?<br>☐ 1=By Month महीने के अनुसार<br>☐ 2=By Year साल के अनुसार			

Unique Variable Name(s)	Source (Year & Item # if Known)	ENGLISH Question & Response Options with Values		Item Conditions	Platform	Site-Specific Modifications
TAB_INSTRUCT_OPC<br><br>OUTPATIENT_CARE		*Display as a table:*<br><br>**Outpatient Attendance:**<br>बाह्यरोगी उपस्थिति<br>For each category listed below, please report the total volume of services or procedures performed each during the previous fiscal year.<br>नीचे दी गयी प्रत्येक कैटेगरी के लिए, पिछले वित्त वर्ष में कुल सेवाओं या किये गये प्रोसीजर की कुल मात्रा बतायें<br>(If this organization has an emergency department, please do not include visits to this department as part of outpatient care.) (यदि इस केंद्र में आपातकालीन विभाग है तो कृपया इस विभाग में आये रोगियों को वाहृय रोगी के रूप में शामिल न करें)<br><br>Enter the following codes, if necessary: आवश्यक होने पर ये कोड लिखें<br>☐ -1=Don't Know नहीं जानते<br>☐ -2=Decline to respond जवाब देने से इंकार<br>☐ -4=Missing data/not observed जानकारी नहीं है/ अवलोकन नहीं किया		(FOR ALL M6 OUTPUT TABLES: 12 Month Tables will be shown for up to 5 years, depending on how long facility has existed)	If have outpatient care	None
			**Last Fiscal Year** पिछला वित्त वर्ष			
OPC_	WHO AFRO	(a) Number of outpatient visits, new patient<br>वाहृय रोगियों की संख्या, नये रोगी				
	WHO AFRO	(b) Number of outpatient visits, old patient<br>वाहृय रोगियों की संख्या, पुराने रोगी				
	IMCI MCE	(c) Number of outpatient visits, adult<br>वाहृय रोगियों की संख्या, वयस्क				
	IMCI MCE	(d) Number of outpatient visits, pediatric<br>वाहृय रोगियों की संख्या, बाल रोगी				
		TOTAL Outpatient Visits कुल आउटपेशेंट विजिट				
		**General Inpatient Questions** सामान्य अन्त: रोगी प्रश्न<br>For this section, please answer the following questions with a facility staff member that is familiar with medical records regarding inpatient visits.<br>इस सेक्शन के लिए कृपया केन्द्र के किसी कर्मचारी की मदद से इनका जवाब दें जो अन्त: रोगी विजिट के बारे में चिकित्सा रिकार्ड से परिचित हो				
OUT_REC		Are inpatient visits recorded at this facility? क्या इस केन्द्र पर आने वाले अन्त:रोगियों का रिकार्ड रखा जाता है?<br>☐ 1=Yes हां<br>☐ 0=No नहीं<br>☐ -1=Don't Know नहीं जानते<br>☐ -2=Decline to respond जवाब देने से इंकार				
INC_MONTH_YEAR		Are inpatient visits recorded by month or by year?<br>क्या अन्त:रोगियों के आने को महीने या साल के रूप में रिकार्ड किया जाता है?<br>☐ 1=By Month महीने के अनुसार<br>☐ 2=By Year साल के अनुसार				

Unique Variable Name(s)	Source (Year & Item # if Known)	ENGLISH<br>Question & Response Options with Values		Item Conditions	Platform	Site-Specific Modifications
TAB_INSTRUCT_IPC INPATIENT_CARE		*Display as a table:*<br><br>**Inpatient Care:**<br>अन्त: रोगियों की देखभाल<br><br>For each category listed below, please report the total volume of services or procedures performed during the previous fiscal year.<br>नीचे दी गयी प्रत्येक कैटेगरी के लिए, पिछले वित्त वर्ष में कुल सेवाओं या किये गये प्रोसीजर की कुल मात्रा बतायें<br><br>Enter the following codes, if necessary: आवश्यक होने पर ये कोड लिखें<br>☐ -1=Don't Know नहीं जानते<br>☐ -2=Decline to respond जवाब देने से इंकार<br>☐ -3=Not applicable नहीं लागू<br>☐ -4=Missing data/not observed जानकारी नहीं है/ अवलोकन नहीं किया		Required show if #INPATIENT==1; (FOR ALL M6 OUTPUT TABLES: 12 Month Tables will be shown for up to 5 years, depending on how long facility has existed)	If have inpatient care	None
			**Last Fiscal Year** पिछला वित्त वर्ष			
IPC_	IHME<br>AHA	Total number of inpatient admissions<br>अन्त:रोगियों की भर्ती की कुल संख्या				
	IHME	(a) Number of pediatric general medicine inpatient admissions<br>(b) सामान्य रोगों वाले भर्ती बाल मरीजों की संख्या				
	IHME	(c) Number of adult general medicine inpatient admissions<br>(d) सामान्य रोगों वाले भर्ती युवा मरीजों की संख्या				
	IHME	(e) Number of surgical inpatient admissions<br>(f) आपरेशन वाले भर्ती मरीजों की संख्या				
	IHME	Total number of inpatient bed days<br>भर्ती मरीजों के बेड दिनों की कुल संख्या				
	IHME	(a) Total number of pediatric general medicine bed days<br>(b) सामान्य रोगों वाले भर्ती बाल मरीजों बेड दिनों की संख्या				
	IHME	(c) Total number of adult general medicine bed days<br>(d) सामान्य रोगों वाले भर्ती युवा मरीजों बेड दिनों की संख्या				
	IHME	(e) Total number of surgical bed days<br>(f) आपरेशन वाले बेड दिनों की कुल संख्या				
	IHME<br>AHA	Total number of inpatient surgical operations<br>भर्ती मरीजों के आपरेशन की कुल संख्या				
	WHO AFRO	(a) Total number of inpatient major surgical operations<br>(b) भर्ती मरीजों के बडे आपरेशन की कुल संख्या				
		(b) Total number of inpatient minor surgical operations<br>भर्ती मरीजों के छोटे आपरेशन की कुल संख्या				
		Total number of in-facility deliveries<br>केन्द्र के अंदर होने वाली डिलीवरी (प्रसव) की कुल संख्या				

Unique Variable Name(s)	Source (Year & Item # if Known)	ENGLISH Question & Response Options with Values			Item Conditions	Platform	Site-Specific Modifications
		Total number of infant death within 24 hrs of birth<br>जन्म के 24 घण्टे के अंदर शिशु मृत्यु की कुल संख्या					
		Total number of infant deaths up to 4 weeks<br>4 सप्ताह तक के शिशु मृत्यु की कुल संख्या					
		Total number of child deaths up to 5 years<br>5 वर्ष तक के शिशु मृत्यु की कुल संख्या					
		Total number of Adolescent/Adult death<br>किशार/युवा मृत्यु की कुल संख्या					
		Total number of maternal deaths<br>मातृ मृत्यु की कुल संख्या					
	IHME	Total number of patients transferred TO THIS FACILITY from another facility for inpatient care<br>दूसरे केन्द्र से इस केन्द्र में स्थानान्तरित किये गये भर्ती होने वाले मरीजों की कुल संख्या					
	IHME	Total number of patients transferred FROM THIS FACILITY to another facility for inpatient care<br>इस केन्द्र से दूसर केन्द्र में स्थानान्तरित किये गये भर्ती होने वाले मरीजों की कुल संख्या					
		**Cardiovascular Disease and Diabetes Questions** हृदय रोग और डाइबिटीज प्रश्न<br>For this section, please answer the following questions with a facility staff member that is familiar with medical records regarding cardiovascular disease and diabetes cases.<br>इस सेक्शन के लिए कृपया केन्द्र के किसी कर्मचारी की मदद से इनका जवाब दें जो हृदय रोग और डाइबिटीज के बारे में चिकित्सा रिकार्ड से परिचित हो					
		Does this facility complete the form called "Monthly Report on Cases and Deaths Due to Non-Communicable Diseases in the State" and have it available?<br>☐ 1=Yes हां<br>☐ 0=No नहीं<br>☐ -1=Don't Know नहीं जानते<br>☐ -2=Decline to respond जवाब देने से इंकार					
		**NCD Record Form**<br>(Repated for each month of the year)<br><br>This section was included half way through data collection and was not translated to Hindi<br><br>Please fill out the following tables according to the information given in the Non-Communicable Disease Record form.<br>If a part of the form is blank or not complete, please put -4 for "missing data"					
		**Out-Patients**					
			Male	Femaile			
		1.1 Rheumatic Fever					
		1.2 Hypertension					
		1.3 Ischemic Heart Disease					
		1.4 Congenital Heart Disease					
		1.5 Other Cardiovscular Diseases					
		3.1 Diabetes Mellitus (Type 1)					
		3.2 Diabetes Mellitus (Type 2)					
		**In-Patients Referred**					

Unique Variable Name(s)	Source (Year & Item # if Known)	ENGLISH Question & Response Options with Values			Item Conditions	Platform	Site-Specific Modifications
			Male	Femaile			
		1.1 Rheumatic Fever					
		1.2 Hypertension					
		1.3 Ischemic Heart Disease					
		1.4 Congenital Heart Disease					
		1.5 Other Cardiovscular Diseases					
		3.1 Diabetes Mellitus (Type 1)					
		3.2 Diabetes Mellitus (Type 2)					
		**IPD Cases Reported Direct**					
			Male	Femaile			
		1.1 Rheumatic Fever					
		1.2 Hypertension					
		1.3 Ischemic Heart Disease					
		1.4 Congenital Heart Disease					
		1.5 Other Cardiovscular Diseases					
		3.1 Diabetes Mellitus (Type 1)					
		3.2 Diabetes Mellitus (Type 2)					
		**Total Deaths During Month**					
			Male	Femaile			
		1.1 Rheumatic Fever					
		1.2 Hypertension					
		1.3 Ischemic Heart Disease					
		1.4 Congenital Heart Disease					
		1.5 Other Cardiovscular Diseases					
		3.1 Diabetes Mellitus (Type 1)					
		3.2 Diabetes Mellitus (Type 2)					
		Does this facility use another method to record cases of cardiovasular disease and diabetes? (Such as cases of myocardial infarction, hypertension, etc.)<br><br>☐ 1=Yes हां<br>☐ 0=No नहीं<br>☐ -1=Don't Know नहीं जानते<br>☐ -2=Decline to respond जवाब देने से इंकार					
IPC_REC		Are cases of cardiovascular disease and diabetes recorded at this facility? (Such as cases of myocardial infarction, hypertention, etc.)<br>क्या इस केन्द्र में हृदय रोग और डाइबिटीज रोगों को रिकार्ड किया जाता है? (ऐसे मामले जैसे मायोकार्डियल इन्फ्रैक्शन, हाइपरटेंशन आदि)<br>☐ 1=Yes हां<br>☐ 0=No नहीं<br>☐ -1=Don't Know नहीं जानते<br>☐ -2=Decline to respond जवाब देने से इंकार					
IPC_MONTH_YEAR		Are cardiovascular disease and diabetes cases recorded by month or by year?<br>क्या हृदय रोग और डाइबिटीज रोग का रिकार्ड महीने या साल के रूप में किया जाता है?<br>☐ 1=By Month महीने के अनुसार<br>☐ 2=By Year साल के अनुसार					

Unique Variable Name(s)	Source (Year & Item # if Known)	ENGLISH<br>Question & Response Options with Values		Item Conditions	Platform	Site-Specific Modifications
		Display as a table:<br><br>**Cardiovascular diseases and diabetes:**<br>हृदय रोग और डाइबिटीज<br><br>For each category listed below, please report the total volume of services or procedures performed during the previous fiscal year.<br>नीचे दी गयी प्रत्येक कैटेगरी के लिए, पिछले वित्त वर्ष में कुल सेवाओं या किये गये प्रोसीजर की कुल मात्रा बतायें<br>Enter the following response codes if necessary: आवश्यक होने पर इन जवाब कोडों को लिखें<br>-1=Don't know नहीं जानते<br>-2=Decline to respond जवाब देने से इंकार<br>-4=Missing/Data not available गायब/जानकारी उपलब्ध नहीं				
			**Last Fiscal Year** पिछला वित्त वर्ष			
		Number of hypertension cases diagnosed<br>जांच पर पाये गये उच्च रक्तचाप के मामलों की संख्या			ALL	
		Number of hypertension patients treated<br>इलाज किये गये उच्च रक्तचाप के मरीजों की संख्या			ALL	
		Number of hypertension-related deaths<br>उच्च रक्तचाप संबंधी मृत्यु की संख्या			ALL	
		Number of myocardial infarction cases (nonSTEMI)<br>मायोकार्डियल इन्फ्रेक्शन मामलों की संख्या (गैर स्टेमी)			ALL	
		Number of myocardial infarction cases (STEMI)<br>मायोकार्डियल इन्फ्रेक्शन मामलों की संख्या (स्टेमी)			ALL	
		Number of myocardial infarction deaths<br>मायोकार्डियल इन्फ्रेक्शन मृत्यु की संख्या			ALL	
		Number of stroke cases diagnosed<br>जांच पर पाये गये आघात मामलों की संख्या			ALL	
		Number of stroke patients treated<br>इलाज किये गये आघात रोगियों की संख्या			ALL	
		Number of stroke-related deaths<br>आघात संबंधी मृत्यु की संख्या			ALL	
		Number of hypercholesterolemia cases diagnosed<br>जांच पर पाये गये हाइपरकोलेस्टेरोलीमिया मामलों की			ALL	
		Number of hypercholesterolemia cases treated<br>इलाज किये गये हाइपरकोलेस्टेरोलीमिया मामलों की संख्या			ALL	
		Number of heart failure cases<br>हार्ट फेल होने वाले मामलों की संख्या			ALL	
		Number of heart failure deaths<br>हार्ट फेल होने से होने वाली मौतों की संख्या			ALL	
		Number of diabetes cases diagnosed<br>जांच पर पाये गये डाइबिटीज मामलों की संख्या			ALL	
		Number of diabetes cases treated<br>इलाज किये गये डाइबिटीज मामलों की संख्या			ALL	

Unique Variable Name(s)	Source (Year & Item # if Known)	ENGLISH Question & Response Options with Values			Item Conditions	Platform	Site-Specific Modifications
		Number of diabetes-related deaths<br>डाइबिटीज से होने वाली मौतों की संख्या				ALL	
		Number of chronic kidney disease patients diagnosed<br>जांच पर पाये गये क्रोनिक किडनी रोगियों की संख्या				ALL	
		Number of chronic kidney disease patients treated<br>इलाज किये गये क्रोनिक किडनी रोगियों की संख्या				ALL	
		Number of chronic kidney disease-related deaths<br>क्रोनिक किडनी से होने वाली मौतों की संख्या				ALL	
		**Preventative Services** रोकथाम संबंधी सेवाएं<br><br>Ask if the facility provides the following preventative programs. If yes, ask how many patients have received this service in the last fiscal year.<br><br>सिर्फ तभी पूछें जब केन्द्र निम्न रोकथाम के कार्यक्रम देता है। यदि हां तो पूछें कि पिछले वित्त वर्ष में कितने मरीजों ने इस सेवा को लिया है।<br><br>-1=Don't know नहीं जानते<br>-2=Decline to respond जवाब से इंकार				All	None
			Is there a specific program or provider that accepts referrals for this? क्या कोई खास कार्यक्रम या कोई प्रोवाइडर है जो इसके रेफरल को स्वीकार करता है?	How many patients have received this service in the last year?पिछले साल में कितने मरीजों ने यह सेवा ली है?			
		Counseling on cessation of tobacco तम्बाकू खाना रोकने के बारे में परामर्श					
		Counseling on cessation of alcohol शराब न पीने के बारे में परामर्श					
		Counseling on weight management वजन नियंत्रित रखने के बारे में परामर्श					
		Nutrition counseling पोषण के बारे में परामर्श					
		Stress management तनाव को नियंत्रित रखना					
		Yoga योगा					
		Meditation ध्यान					
		Promotion of physical activity शारीरिक गतिविधियों को बढावा					
		Diagnosis of uncomplicated hypertension गैर जटिल तनाव की जांच					
		Diagnosis of Hyperlipidemia/high cholesterol हाइपरलिपिडेमिया/हाई कोलेस्ट्राल की जांच					
		Prescription of treatment of hyperlimiedia हाइपरलीमिडिया के इलाज का प्रेस्क्रिप्शन					
		Prescription of treatment of uncomplicated hypertension गैर जटिल हाइपरटेंशन के इलाज का प्रेस्क्रिप्शन					
		Prescription of treatment of uncomplicated diabetes गैर जटिल डाइबिटीज के इलाज का प्रेस्क्रिप्शन					

Unique Variable Name(s)	Source (Year & Item # if Known)	ENGLISH<br>Question & Response Options with Values	Item Conditions	Platform	Site-Specific Modifications
		Follow-up treatment of diagnosed cases of hypertension<br><br>जांच कर पाये गये उच्चरक्तचाप के मामलों में फालोअप इलाज			
		Connecting to quit lines for tobacco/alcohol cessation<br><br>तम्बाकू खाना छोडना/ शराब पीना बन्द करना			
		**Emergency Care Questions** आकस्मिक देखभाल संबंधी प्रश्न<br>For this section, please answer the following questions with a facility staff member that is familiar with medical records regarding emergency visits.<br>इस सेक्शन के लिए कृपया केन्द्र के किसी कर्मचारी की मदद से इनका जवाब दें जो इमर्जेन्सी विजिट के बारे में चिकित्सा रिकार्ड से परिचित हो			
EC_YES	IHME<br>AHA	Does this facility have an emergency department?<br>क्या इस केन्द्र में आपातकालीन विभाग है?<br>(SELECT ONE)<br>☐ 1= yes हां<br>☐ 0= no नहीं<br>☐ -1=Don't know नहीं जानते<br>☐ -2=Decline to respond जवाब देने से इंकार		All except SHC	
IPC_REC		Are cases of emergency department visits recorded at this facility?<br>क्या इस केन्द्र में इमर्जेन्सी विभाग के मामलों को रिकार्ड किया जाता है?<br>☐ 1=Yes हां<br>☐ 0=No नहीं<br>☐ -1=Don't Know नहीं जानते<br>☐ -2=Decline to respond जवाब देने से इंकार			
ED_PED_AGE		For accident and emergency cases, what ages are considered pediatric?<br>दुर्घटना और आपातकालीन मामलों के लिए, किस उम्र को बाल चिकित्सा माना जाता है?<br>(SELECT ONE)<br>☐ 5= 0-12 years 0-12 वर्ष<br>☐ 4= 0-14 years 0-14 वर्ष<br>☐ 3= 0-15 years 0-15 वर्ष<br>☐ 2= 0-16 years 0-16 वर्ष<br>☐ 1= 0-19 years 0-19 वर्ष<br>☐ 0= Other (specify) अन्य, स्पष्ट करें<br>☐ -1=Don't know नहीं जानते<br>☐ -2=Decline to respond जवाब देने से इंकार<br>☐ -3=Not applicable नहीं लागू	Show if EC_YES=1		
ED_PED_AGE_OSP		Please specify 'other': ___________________________<br>कृपया 'अन्य' को स्पष्ट करें			

Unique Variable Name(s)	Source (Year & Item # if Known)	ENGLISH Question & Response Options with Values		Item Conditions	Platform	Site-Specific Modifications
EC_MONTH_YEAR		Are emergency department visits recorded by month or by year?<br>क्या इमर्जेन्सी विभाग के मामलों को महीने या साल के रूप में रिकार्ड किया जाता है?<br>☐ 1=By Month महीने के अनुसार<br>☐ 2=By Year साल के अनुसार				
TAB_INSTRUCT_EC<br><br>EMERGENCY_CARE	IHME	*Display as a table:*<br><br>**Emergency Care:**<br>आकस्मिक देखभाल<br><br>For each category listed below, please report the total volume of services or procedures performed during the previous fiscal year. Include visits to the Casualty Department.<br>नीचे दी गयी प्रत्येक कैटेगरी के लिए, पिछले वित्त वर्ष में कुल सेवाओं या किये गये प्रोसीजर की कुल मात्रा बतायें। कैजुएल्टी विभाग में आने वालों को भी शामिल करें<br><br>Enter the following codes, if necessary: आवश्यक होने पर नीचे दिये गये कोड लिखें<br>-1=Don't know नहीं जानते<br>-2=Decline to respond जवाब से इंकार<br>-4=Missing/Data not available गायब/जानकारी उपलब्ध नहीं<br><br>Please note, for all tables such as the below, use the "Tab" key to move to the next cell space<br>कृपया ध्यान दें कि नीचे की सभी तालिकाओं के लिए अगले सेल में जाने के लिए 'टैब' की का प्रयोग करें।		(FOR ALL M6 OUTPUT TABLES: 12 Month Tables will be shown for up to 5 years, depending on how long facility has existed)	Show if EC_YES == 1	None
			**Last Fiscal Year** पिछला वित्त वर्ष			
		Total number of emergency department visits<br>आपातकालीन विभाग में आने वाले लोगों की कुल संख्या				
EC	IHME<br>AHA	(a) Total number of emergency department visits, adult<br>आपातकालीन विभाग में आने वाले लोगों की कुल संख्या - युवा				
	IHME	(b) Total number of emergency department visits, pediatric<br>आपातकालीन विभाग में आने वाले लोगों की कुल संख्या - बच्चे				
	IHME	Total number of patients transported FROM THIS FACILITY'S EMERGENCY DEPARTMENT to another facility for inpatient care<br>इस केन्द्र के आकस्मिक विभाग से दूसरे केन्द्र में स्थानान्तरित किये गये मरीजों की कुल संख्या				

Unique Variable Name(s)	Source (Year & Item # if Known)	ENGLISH<br>Question & Response Options with Values		Item Conditions	Platform	Site-Specific Modifications
		**Laboratory and Diagnotic Test Questions** प्रयोगशाला और जांच संबंधी प्रश्न<br>For this section, please answer the following questions with a facility staff member that is familiar with records regarding laboratory and diagnostic tests performed at this facility.<br>इस सेक्शन के लिए कृपया केन्द्र के किसी कर्मचारी की मदद से इनका जवाब दें जो इस केन्द्र पर जांच और किये जाने वाले परीक्षण के बारे में रिकार्ड से परिचित हो				
LAB_YES	IHME<br>AHA	Did this facility perform any laboratory or diagnostic tests in-facility in the previous year?<br>क्या पिछले साल में इस केन्द्र ने कोई लैब या जांच परीक्षण अपने केन्द्र में की?<br>(SELECT ONE)<br>☐ 1= yes हां<br>☐ 0= No नहीं<br>☐ -1 = Don't Know नहीं जानते<br>☐ -2= Decline to Respond जवाब से इंकार				
		Are laboratory or diagnostic tests recorded at this facility? क्या इस केन्द्र पर जांच और परीक्षण टेस्ट को रिकार्ड किया जाता है?<br>☐ 1=Yes हां<br>☐ 0=No नहीं<br>☐ -1=Don't Know नहीं जानते<br>☐ -2=Decline to respond जवाब देने से इंकार				
		Are laboratory or diagnostic tests recorded by month or by year?<br>क्या जांच और परीक्षण टेस्ट महीने के आधार पर रिकार्ड किया जाता है या साल के आधार पर?<br>☐ 1=By Month महीने के अनुसार<br>☐ 2=By Year साल के अनुसार				
TAB_INSTRUCT_TESTS<br><br>LABS_DX_TESTS	IHME	*Display as a table:*<br><br>**Laboratory and Diagnostic Tests:**<br>प्रयोगशाला एवं परीक्षण जांचें<br><br>For each category listed below, please report the total number of laboratory or diagnostic tests performed by this facility during the previou fiscal year.<br>नीचे दी गयी प्रत्येक कैटेगरी के लिए, पिछले वित्त वर्ष में कुल सेवाओं या किये गये प्रोसीजर की कुल मात्रा बतायें<br>Enter the following codes, if necessary: आवश्यक होने पर ये कोड लिखें<br>-1=Don't know नहीं जानते<br>-2=Decline to respond जवाब से इंकार<br>-4= Missing/Data not available गायब/ जानकारी उपलब्ध नहीं		(FOR ALL M6 OUTPUT TABLES: 12 Month Tables will be shown for up to 5 years, depending on how long facility has existed)	If perform lab test	None
			**Last Fiscal Year** पिछला वित्त वर्ष			
		Total number of medical imaging tests (all types including xrays, ultrasound, CT scans, etc.)<br>मेडिकल इमेजिंग टेस्ट की कुल संख्या (सभी प्रकार के एक्सरे, अल्ट्रासाउण्ड, सीटी स्कैन आदि)				
		Total number of x-rays<br>एक्सरे की कुल संख्या				

Unique Variable Name(s)	Source (Year & Item # if Known)	ENGLISH Question & Response Options with Values		Item Conditions	Platform	Site-Specific Modifications
		Total number of ultrasounds (pregnancy-related)<br>अल्ट्रासाउण्ड की कुल संख्या (गर्भावस्था संबंधी)				
		Total number of ultrasounds (non pregnancy-related excluding echos)<br>अल्ट्रासाउण्ड की कुल संख्या (इको को छोडकर गैर-गर्भावस्था संबंधी)				
		Total number of Echocardiograms<br>ईकोकार्डियोग्राम की कुल संख्या				
		Total number of Exercise treadmill stress tests<br>एक्सरसाइज ट्रेडमिल स्ट्रेस टेस्ट की कुल संख्या				
		Total number of stress echocardiograms<br>स्ट्रेस ईकोकार्डियोग्राम की कुल संख्या				
		Total number of Stress myocardial perfusion imaging<br>स्ट्रेस माइकार्डियल परफ्यूजन इमेजिंग				
		Total number of CT scans सीटी स्कैल की कुल संख्या				
		Total number of MRIs एमआरआई की कुल संख्या				
		Total number of ECGs ईसीजी की कुल संख्या				
WHO_ASSISTED	IHME	What were the positions of the respondents who helped you fill in this section of the survey?<br>उन उत्तरदाताओं का पद क्या था? जिन्होंने सर्वेक्षण के इस खंड को भरने में आपकी मदद की?<br>(SELECT ALL THAT APPLY) (एक से अधिक उत्तर सम्भव)<br>☐ 1 =Facility Administrator केन्द्र प्रशासक<br>☐ 2 = Doctor डाक्टर<br>☐ 3 = Nurse नर्स<br>☐ 4 = Pharmacist फार्मासिस्ट<br>☐ 5= Laboratory Technition लैब टेक्नीशियन<br>☐ 6= Accountant एकाउंटेन्ट<br>☐ 7= Human Resource Manager मानव संसाधन प्रबन्धक<br>☐ 8= HMIS Manager एचएमआईएस मैनेजर<br>☐ 9= Clerk क्लर्क<br>☐ 10 = Other Staff, Specify अन्य कर्मचारी, स्पष्ट करें	required;	All	None	
COMMENTS2	IHME	Please use this space to provide us with any feedback/comments/issues you encountered while completing this section.<br>इस खंड को पूरा करने में आपका सामना जिस भी प्रतिक्रिया / टिप्पणी / मुद्दों के साथ हुआ है उसे हमें प्रदान करने के लिए नीचे दिए गए स्थान का प्रयोग करें.				

Unique Variable Name(s)	Source (Year & Item # if Known)	ENGLISH Question & Response Options with Values		Item Conditions	Platform	Site-Specific Modifications
		Thank you for participating in the Facility Survey Module 1: Inputs, Finances and Management<br>केन्द्र के सर्वे माडयूल 1 : जानकारी, वित्त और प्रबन्धन में भाग लेने के लिए आपको धन्यवाद<br>Click 'Get Time' button. 'गेट टाइम' बटन पर क्लिक करें				
