# Supplementary material for: Identifying gaps in the continuum of care for hypertension and diabetes in two Indian communities
Source: BMC Health Serv Res. 2017 Dec 27;17:846. doi: 10.1186/s12913-017-2796-9 (PMC5746011; doi:10.1186/s12913-017-2796-9)
Supplement: Supplementary file 3 — Focus group discussion protocols for patients and community members. (PDF 889 kb) [file 12913_2017_2796_MOESM3_ESM.pdf]

NCD Patients

Focus Group Protocol Package

Objectives:	To assess access, experience, attitudes, and behaviors related to NCD care among NCD patients.
Methodology:	Focus Groups (8-10 participants each)
Target participants:	NCD Patients:<br><br>Group 1: Female young adults (age 18-40)<br>Group 2: Female older adults (age 41 and above)<br>Group 3: Male young adults (age 18-40)<br>Group 4: Male older adults (age 41 and above)
Recruitment strategy:	NCD patients coming into the health care facility for an appointment will be asked at reception/check-in if they would be willing to participate in a focus group as part of the study. If positively inclined, a member of the study team will be on hand (before or after the appointment) to answer any questions and to review consent.

FOCUS GROUP INFORMATION SHEET[1]

Type of focus group:

- ☐ Community Members (Non-Patients) **[CM]**
- ☑ NCD Patients **[NCDP]**
- ☐ Front-Line Health Care Providers **[HCP]**
- ☐ Community Leaders (including NGOs and CBOs) **[CL]**

Catchment area:

District: ________________________________

City/Town: ________________________________

GPS coordinates of health care facility: __________________________

Health care facility information:

**applicable to NCD Patients and Front-Line Health Care Provider groups only*

☐ Hospital	☐ Public
☐ Primary Health Centre	☐ Private
☐ Community Health Centre	
☐ Sub-Centre	

Number of focus group participants: ______________________

Group gender: ☐ Male ☐ Female

**applicable to Community Member (Non-Patient) and NCD Patient groups only*

Group age range: ☐ 18-40 years ☐ 41 years and above

**applicable to Community Member (Non-Patient) and NCD Patient groups only*

[1] **To be filled by focus group facilitator and attached to focus group transcriptions and/or notes**

Date of Focus Group: ____

Name of facilitator: ____

Name of note taker/electronic recorder: ____

Focus Group Code: **__I-FG-NCDP-XX**_______________________________________

**used to label and match all hardcopy and electronic files, transcript tapes, etc. and can be used in master file of qualitative data collection. Should include country, focus group vs. interview, and category of participants. For example, for focus groups in India with NCD Patients you use: I-FG-NCDP-1, I-FG-NCDP-2, etc.*

[FOCUS GROUP FACILITATOR AND NOTE TAKER(S) SHOULD BE WEARING A NAME TAG WITH FIRST NAME. AS PARTICIPANTS ENTER THE ROOM, THEY SHOULD BE ASKED TO FILL A NAME TAG FOR THEMSELVES WITH FIRST NAME OR INITIALS ONLY. THE IDEA IS TO HAVE AN IDENTIFIER FOR FACILITATING THE DISCUSSION AND FOR NOTE-TAKING (E.G., BEING ABLE TO NOTE WHEN SPEAKER HAS CHANGED), BUT NOT TO IDENTIFY INDIVIDUALS WITH THEIR FULL NAME. FOR NOTETAKING, USE INITIALS ONLY]

Introduction

Thank you for agreeing to participate in this focus group today. Before we get started, I would like review the overall purpose of our study and why we have asked you to come here today. I also will review how the information you provide to us today will be used, and obtain your agreement to participate, once this information is shared with you. Finally, I will review a few ground rules on how we will conduct the discussion.

[READ INFORMED CONSENT WHICH SHOULD INCLUDE THE OVERALL PURPOSE OF THE STUDY AND HOW THE FOCUS GROUPS INTERVIEWS ARE AN IMPORTANT COMPONENT. OBTAIN INFORMED CONSENT AND LEAVE THE INFORMATION SHEET ABOUT THE PROJECT WITH PARTICIPANTS, INCLUDING CONTACT INFORMATION FOR THE IN-COUNTRY STUDY LEAD FOR ANY FOLLOW-UP QUESTIONS.]

OK, just a few ground rules before we get started:

- *First, only one person should talk at a time.*
- *Second, no one person or combinations of persons should dominate the discussion. It is important to hear everyone's ideas and opinions.*
- *Third, everyone's ideas, experiences, and opinions should be respected--even if they are different than yours. It is important to express different perspectives, and it is OK to express disagreement, but to do so in a respectful way.*
- *As facilitator, I will take charge of keeping order and organization to our discussion, including keeping the discussion on track, ensuring that we have opportunities to hear from everyone, and maintaining order and respect throughout the discussion.*
- *Does anyone have any additional ground rules that they would like to add?*

[IN THE FOLLOWING INTERVIEW GUIDE, HIGH-LEVEL QUESTIONS SHOULD BE ASKED FIRST WITH OPPORTUNITIES FOR PARTICIPANTS TO ANSWER AND REPLY BEFORE MOVING ON TO PROBES. NOT ALL PROBES NEED TO BE USED. ONLY USE PROBES AS REQUIRED: 1) TO STIMULATE RESPONSES WHEN YOU ARE NOT GETTING THEM; OR 2) TO ENSURE THE TOPIC HAS BEEN COVERED COMPREHENSIVELY BEFORE MOVING ON.]

Beliefs About and Attitudes Toward Health and Health Care

We are interested in broadly understanding how people in your community view health and healthcare services.

- Can you describe in your own words what it means to be healthy?
- What does healthcare mean to you? What does it include?
- How would you describe the overall health status of people in your community?
  - Probe: General levels of health?
  - Probe: Major issues? NCD vs. CD; chronic vs. acute
- How would you describe the overall status of health care in your community?
  - Probe: Views with respect to the quality of care across different care settings (public/private/NGO; clinic/hospital; other)
  - Probe: For NCD vs. CD; chronic vs. acute
- Where do people in your community typically get information about health and health care?
  - Probe: information about health?
  - Probe: information about health care and health care services?
  - Probe: Different for different groups of people? Explain.
- Where do you typically get information about health and health care?

Care-seeking Practices

Now, we would like to better understand when, how, and where you seek health care services.

- When have you sought/do you seek care from health professionals?
  - Probe: Do you get regular check-ups or exams (preventive care) even when you are feeling OK? If no, why? If yes, where and why?
  - Probe: Did you get regular check-ups or exams (preventive care) before you learned of your condition? If no, why? If yes, where and why?
  - Probe: How did you come to learn that you had a health condition that needed treatment?
  - Probe: What were the key factors that encouraged you to seek care and treatment?
- Where do you generally go for treatment/follow-up related to your condition?
  - Probe: Different places for different situations/needs?
  - Probe: Why?
- Where do you go for other types of health care needs?
  - Probe: [ask about both types of health care needs and types of health care settings]
  - Probe: Why?
- How often do you visit health providers, in general?
  - Probe: How is this “broken down” by type of provider/type of situation?
  - Probe: Do you think that you go to the doctor/visit health professionals more often than your family and friends?
- How often do you seek care from someone other than a doctor (as a proportion of the above)?
  - Probe: From where/whom do you seek care, depending on the situation?
  - Probe: Why? (e.g., cost, trust, wait time, etc.)

Care Experiences

- What has been your experience in obtaining care here {if on site in a health facility} and in other health care settings? [or just simply: What has been your experience in obtaining care across different care settings in your community?]
  - Probe: Are you generally satisfied with the health care that you receive in your community? Why or why not?
  - Probe: In general, do you think that going to a clinic or hospital when you're sick or in need of treatment will usually result in you feeling better and being healthier?
  - Probe: How do you view private versus public hospitals or clinics in your community? How do you think they are different? How are they the same? Other types of care settings?
  - Probe: Do you feel you are treated with respect when you go to a hospital or clinic? Do you feel that the medical personnel are knowledgeable and trustworthy?
- How do you choose which health facility to go to?
  - Probe: Different for different situations?
  - Probe: Proximity to home, cost, wait times, access, etc.?
- When you seek health care, from a doctor or other type of provider, do you generally follow the advice you are given?
  - Probe: Do you adhere to your NCD treatment plan?
  - Probe: If yes, what are the key factors that encourage/support your adherence?
  - Probe: If not, why not? (e.g., cost, lack of understanding, lack of belief in efficacy, too difficult/complicated etc.)
  - Probe: Does level of adherence differ by type of health care provider? In what way and why?
- Whose medical opinion or advice do you trust most in the community?
  - Probe: Who do you think is most knowledgeable about medicine in your community? (Doctors, Nurses, CHW's, etc.)
  - Probe: Does this differ depending on the situation/need? Explain.

Access to Healthcare

- What types of healthcare do you think people in your community can access easily?
  - Probe: by types of care - preventive, acute, chronic disease management, etc.
  - Probe: by types of setting – private/public/NGO, clinics, etc.
  - Probe: Why? (e.g., cost, information, etc.)
- What types of healthcare do you think it is hard for people in your community to access?
  - Probe: by types of care - preventive, acute, chronic disease management, etc.
  - Probe: by types of setting – private/public/NGO, clinics, etc.
  - Probe: Why? (e.g., cost, travel requirements, etc.)
- What groups of people might have difficulty accessing healthcare in your community?
  - Probe: by SES, social group, age?
  - Probe: Why? (e.g., cost, transportation, work, uninformed, etc.)
- What do you see as the primary barriers to access to healthcare in your community?
  - Probe: (e.g., cost, lack of providers, wait times, etc.)

  - Probe: Does this differ by types of care - preventive, acute, chronic disease management, etc.?
  - Probe: How does this differ by setting – private/public/NGO, clinics, etc.?
  - Probe: How does this differ by population groups?
- Can you describe your experiences in accessing healthcare in your community?
  - Probe: Things that have been easy/difficult?
  - Probe: Levels of satisfaction with access, quality of care, etc.
  - Probe: Comparison of access across types of care needs and settings

General Assessment of Health Care Services and Ideas for Improvement

- What do you see as good resources in your community for maintaining good health?
- What do you see as resources lacking in your community for maintaining good health?
- What health resources could be improved in your community?
- What do you think the health care facilities, community leaders, or the government has done well in providing healthcare to the community?
  - Probe: Can you provide some examples? (e.g., specific programs or public health campaigns)
- What do you think the health care facilities, community leaders, or the government has done less well in providing healthcare to the community?
  - Probe: Can you provide some examples?
- Finally, what other kinds of things do you think could be done to improve the overall health of people in your community?
  - Probe: What kinds of programs do you think could improve health or health care in your community?
  - Probe: What ideas do you have?

Thank you all for your time and participation. The information and insights that you have provided today will be incredibly valuable to this project. If you have any questions, please feel free ask me or to contact me at the number/email listed on your study information sheet.

COMMUNITY MEMBERS (NON-PATIENTS)

FOCUS GROUP PROTOCOL PACKAGE

Objectives: To assess general beliefs, attitudes, and practices with respect to health and health care in the community. To community level assess access (barriers and opportunities) to non-communicable disease care and other health care.

Methodology: Focus Groups (8-10 participants each)

Target participants: Non-patients of different sociodemographic backgrounds[2]:

Group 1: Female young adults (age 18-40)
Group 2: Female older adults (age 41 and above)
Group 3: Male young adults (age 18-40)
Group 4: Male older adults (age 41 and above)

Recruitment strategy:

Will recruit participants for each focus group through non-health-related community organizations and institutions (e.g., educational institutions, places of worship), dependent on the target group. To assure variation on SES, may need to recruit from different institutions for the same focus group.

[2] Note that groupings by age will likely change slightly, based on country. This range given is standardized for India's protocol. In the US, HealthRise will likely add another "middle age" range to the protocol, separate from "older adults".

FOCUS GROUP INFORMATION SHEET[3]

Type of focus group:

- ☑ Community Members (Non-Patients) **[CM]**
- ☐ NCD Patients **[NCDP]**
- ☐ Front-Line Health Care Providers **[HCP]**
- ☐ Community Leaders (including NGOs and CBOs) **[CL]**

Catchment area: District: ________________________________

GPS coordinates of focus group location: ___________________________

Health care facility information:
**applicable to NCD Patients and Front-Line Health Care Provider groups only*

☐ Hospital	☐ Public
☐ Primary Health Centre	☐ Private
☐ Community Health Centre	
☐ Sub-Centre	

Number of focus group participants: ______________________

Group gender: ☐ Male ☐ Female
**applicable to Community Member (Non-Patient) and NCD Patient groups only*

Group age range: ☐ 18-40 years ☐ 41 years and above
**applicable to Community Member (Non-Patient) and NCD Patient groups only*

---

[3] **To be filled by focus group facilitator and attached to focus group transcriptions and/or notes**

Date of Focus Group: ____

Name of facilitator: ____

Name of note taker/electronic recorder: ____

Focus Group Code: __I-FG-CM-XX______________________________________

**used to label and match all hardcopy and electronic files, transcript tapes, etc. and can be used in master file of qualitative data collection. Should include country, focus group vs. interview, and category of participants. For example, for focus groups in India with Community Members (Non-Patients), you use: I-FG-CM-1, I-FG-CM-2, etc.*

[FOCUS GROUP FACILITATOR AND NOTE TAKER(S) SHOULD BE WEARING A NAME TAG WITH FIRST NAME. AS PARTICIPANTS ENTER THE ROOM, THEY SHOULD BE ASKED TO FILL A NAME TAG FOR THEMSELVES WITH FIRST NAME OR INITIALS ONLY. THE IDEA IS TO HAVE AN IDENTIFIER FOR FACILITATING THE DISCUSSION AND FOR NOTE-TAKING (E.G., BEING ABLE TO NOTE WHEN SPEAKER HAS CHANGED), BUT NOT TO IDENTIFY INDIVIDUALS WITH THEIR FULL NAME. FOR NOTETAKING, USE INITIALS ONLY]

Introduction

Thank you for agreeing to participate in this focus group today. Before we get started, I would like review the overall purpose of our study and why we have asked you to come here today. I also will review how the information you provide to us today will be used, and obtain your agreement to participate, once this information is shared with you. Finally, I will review a few ground rules on how we will conduct the discussion.

[READ INFORMED CONSENT WHICH SHOULD INCLUDE THE OVERALL PURPOSE OF THE STUDY AND HOW THE FOCUS GROUPS INTERVIEWS ARE AN IMPORTANT COMPONENT. OBTAIN INFORMED CONSENT AND LEAVE THE INFORMATION SHEET ABOUT THE PROJECT WITH PARTICIPANTS, INCLUDING CONTACT INFORMATION FOR THE IN-COUNTRY STUDY LEAD FOR ANY FOLLOW-UP QUESTIONS.]

OK, just a few ground rules before we get started:

- *First, only one person should talk at a time.*
- *Second, no one person or combinations of persons should dominate the discussion. It is important to hear everyone's ideas and opinions.*
- *Third, everyone's ideas, experiences, and opinions should be respected--even if they are different than yours. It is important to express different perspectives, and it is OK to express disagreement, but to do so in a respectful way.*
- *As facilitator, I will take charge of keeping order and organization to our discussion, including keeping the discussion on track, ensuring that we have opportunities to hear from everyone, and maintaining order and respect throughout the discussion.*
- *Does anyone have any additional ground rules that they would like to add?*

[IN THE FOLLOWING INTERVIEW GUIDE, HIGH-LEVEL QUESTIONS SHOULD BE ASKED FIRST WITH OPPORTUNITIES FOR PARTICIPANTS TO ANSWER AND REPLY BEFORE MOVING ON TO PROBES. NOT ALL PROBES NEED TO BE USED. ONLY USE PROBES AS REQUIRED: 1) TO STIMULATE RESPONSES WHEN YOU ARE NOT GETTING THEM; OR 2) TO ENSURE THE TOPIC HAS BEEN COVERED COMPREHENSIVELY BEFORE MOVING ON.]

Beliefs About and Attitudes Toward Health and Health Care [15 minutes]

OK, let's start with some broad questions about how you and people in your community view health and healthcare services.

- Can you describe in your own words what it means to be healthy?
  - Probe: What words or images come to mind when you think about being healthy?
- What does health <u>care</u> mean to you?
  - Probe: What does health care include?
  - Probe: Self-care? Preventive-care? Or only curative care?
- How would you describe the overall health status of people in your community?
  - Probe: In general, are people very healthy/somewhat healthy/not very healthy in your community? Why?
  - Probe: What are the major health issues in your community?
  - Probe: Non-communicable diseases (NCD) (like diabetes, asthma, high blood pressure) vs. communicable diseases (CD) (like HIV, hepatitis, etc.)?
  - Probe: Chronic long-term diseases vs. acute illnesses?
- How would you describe the overall status of health <u>care</u> in your community?
  - Probe: What is the general quality of health care provided?
  - Probe: Does the quality of care differ across care settings? (public vs. private vs. NGO providers?; hospitals vs. clinics?)
  - Probe: Does the quality of care differ across type of health care need? (NCD vs. CD; chronic vs. acute illness?)
- Where do people in your community typically get information about health and health care?
  - Probe: Where can you get information about ways to stay healthy?
  - Probe: Where do people typically get information about the availability of health care services?
  - Probe: Is the availability of information about health and health care different for different groups of people in your community? Explain.

Care-seeking Practices [15 minutes]

OK, let's move on to talk about both when and how people in your community seek health care services.

- To start off, under what circumstance do people tend to seek care from health professionals in your community?
  - Probe: Only when they are sick? How sick?
  - Probe: Do people get regular check-ups or exams (preventive care) even when they are feeling OK? If no, why? If yes, where and why?
- <u>Where</u> do people in your community generally go when they are not feeling well?
  - Probe: Different places for different situations/needs?
  - Probes for places: hospital, clinic, community health worker, alternative medicine provider, local pharmacy
  - Probes for different situations: minor illness or ailment, serious injury, illness lasting more than 3 days and impacting ability to function in daily life, etc.
- How often do people visit health providers, in general?

- Probe: Generally healthy people?
- Probe: People with some sort of chronic disease (e.g., asthma, CVD, diabetes)?

- How often do people seek care from someone other than a doctor (as a proportion of the above)?
  - Probe: Generally healthy people?
  - Probe: People with some sort of chronic disease (e.g., cardiovascular disease (CVD), diabetes, asthma)?
  - Probe: From where/whom do they seek care, depending on the situation? [PROVIDE EXAMPLES OF CARE GIVERS IN COMMUNITY OTHER THAN DOCTORS]
  - Probe: Why? (e.g., cost, trust, wait time, etc.)
- When you seek health care, from a doctor or other type of provider, do you generally follow the advice you are given?
  - Probe: Why or why not? (e.g., cost, lack of understanding, lack of belief in efficacy, etc.)
  - Probe: Does this differ by type of health care provider? [PROVIDE LIST OF DIFFERENT TYPES OF HEALTH CARE PROVIDERS AVAILABLE IN THE COMMUNITY]
  - Probe: In what way and why?
- Whose medical opinion or advice do you trust most in the community?
  - Probe: Who do you think is most knowledgeable about medicine in your community? [PROVIDE LIST OF DIFFERENT TYPES OF HEALTH CARE PROVIDERS AVAILABLE IN THE COMMUNITY]
  - Probe: Does this differ depending on the situation/need? Explain.

Access to Healthcare [30 minutes]

- What types of healthcare do you think people in your community can access easily?
  - Probe by types of care:
    - Preventive care?
    - Acute care?
    - Chronic disease management? -
  - Probe by types of setting:
    - Private hospitals and clinics?
    - Public hospitals and clinics?
    - Non-governmental and community based organizations
  - Probe: Why? (e.g., cost, information, etc.)
- What types of healthcare do you think it is hard for people in your community to access?
  - Probe by types of care:
    - Preventive care?
    - Acute care?
    - Chronic disease management? -
  - Probe by types of setting:
    - Private hospitals and clinics?
    - Public hospitals and clinics?
  - Non-governmental and community based organizations
  - Probe: Why? (e.g., cost, travel requirements, etc.)

- What groups of people might have difficulty accessing healthcare in your community?
  - Probe: by socioeconomic status, social group, age [maybe add specific groups of interest as probes here]
  - Probe: Why? (e.g., cost, transportation, work, uninformed, etc.)
- What do you see as the primary barriers to access to healthcare in your community?
  - Probe: (e.g., cost, lack of providers, wait times, etc.)
  - Probe: Does this differ by types of care - preventive, acute, chronic disease management, etc.?
  - Probe: How does this differ by setting – private vs. public vs. NGO and CBO clinics; tier levels?
  - Probe: How does this differ by population groups? [add specific groups here as examples/probes]
- Can you describe your own experiences in accessing healthcare in your community?
  - Probe: Things that have been easy to access? (in terms of both care settings and types of care)
  - Probe: Things that have been difficult to access? (in terms of both care settings and types of care)
  - Probe: What is your general level of satisfaction with access to care?
  - Probe: What is your general level of satisfaction with quality of care?
    - Probe: By type of care setting?
    - Probe: By types of care?

General Assessment of Health Care Services and Ideas for Improvement [15 minutes]

- What do you see as good resources in your community for maintaining good health?
  - Probe: Mobile health programs? Public health campaigns?
- What health resources could be improved in your community?
  - Probe: Mobile health programs? Public health campaigns?
- What do you think the health care facilities, community leaders, or the government has done well in providing healthcare to the community?
  - Probe: Can you provide some examples? (e.g., specific programs or public health campaigns)
- What do you think the health care facilities, community leaders, or the government has done less well in providing healthcare to the community?
  - Probe: Can you provide some examples?
- Finally, what other kinds of things do you think could be done to improve the overall health of people in your community?
  - Probe: What kinds of programs do you think could improve health or health care in your community?
  - Probe: What ideas do you have?

Thank you all for your time and participation. The information and insights that you have provided today will be incredibly valuable to this project. If you have any questions, please feel free ask me or to contact me at the number/email listed on your study information sheet.
