# Supplementary material for: Identifying gaps in the continuum of care for hypertension and diabetes in two Indian communities
Source: BMC Health Serv Res. 2017 Dec 27;17:846. doi: 10.1186/s12913-017-2796-9 (PMC5746011; doi:10.1186/s12913-017-2796-9)
Supplement: Supplementary file 4 — Key informant interview protocols for physicians, health system officials/policymakers and nurses/ASHAs. (PDF 943 kb) [file 12913_2017_2796_MOESM4_ESM.pdf]

FRONT-LINE HEALTH CARE PROVIDERS: DOCTORS/PHYSICIANS

KEY INFORMANT INTERVIEW PROTOCOL PACKAGE

Objectives: To gather information on access to NCD care and experiences in the provision of that care from the perspective of NCD care givers.

Methodology: Interviews

Target participants: 1 Doctor/Physician from each facility type: Private clinic, public district hospital, Primary Health Centre, Community Health Centre, Sub-Centre.

If no doctor or physician is available at smaller facilities, the highest-ranking health official should be targeted.

Recruitment strategy:

An initial communication about the overall study will come from facility management, explaining the purpose of the study and that both patients, providers, and administrators/management at the facility will be asked to participate. Follow-up communication to relevant staff will come from either facility management or the research team directly, depending on the arrangements made facility by facility. Volunteers will be asked schedule the interview on a specified date and time and will be informed of the purpose of the discussion as well as the nature of the questions (i.e., non-sensitive and non-personal) ahead of time—and that the session will be audio recorded for transcription and study. Oral informed consent will be obtained at the start of the interview.

KEY INFORMANT INTERVIEW INFORMATION SHEET[1]

Type of interview:

☑ Physicians/Doctors **[PD]**

☐ Nurses and ASHAs **[NASH]**

☐ Health Officials **[HO]**

Catchment area:

District: ________________________________

City/Town: ________________________________

Name of health care facility: __________________________

Health care facility information:

☐ Hospital

☐ Primary Health Centre

☐ Community Health Centre

☐ Sub-Centre

☐ Public

☐ Private

Interviewee job title: ____________________

Date of Interview: ____

Name of facilitator: ____

Name of note taker/electronic recorder: ____

Interview Code: **__I-KII-PD-XX______________________________________**

**used to label and match all hardcopy and electronic files, transcript tapes, etc. and can be used in master file of qualitative data collection. Should include country, focus group vs. interview, and category of participants. For example, for interviews with Physicians or doctors in India, you use: I-KII-PD-1, I-KII-PD-2, etc.*

[1] **To be filled by focus group facilitator and attached to focus group transcriptions and/or notes**

KEY INFORMANT INTERVIEW GUIDE: DOCTORS AND PHYSICIANS

Introduction

Thank you for agreeing to participate in this key informant interview today. Before we get started, I would like review the overall purpose of our study and why we have asked you to come here today. I also will review how the information you provide to us today will be used, and obtain your agreement to participate, once this information is shared with you.

[READ INFORMED CONSENT WHICH SHOULD INCLUDE THE OVERALL PURPOSE OF THE STUDY AND HOW THE FOCUS GROUPS INTERVIEWS ARE AN IMPORTANT COMPONENT. OBTAIN INFORMED CONSENT AND LEAVE THE INFORMATION SHEET ABOUT THE PROJECT WITH PARTICIPANT, INCLUDING CONTACT INFORMATION FOR THE IN-COUNTRY STUDY LEAD FOR ANY FOLLOW-UP QUESTIONS.]

[IN THE FOLLOWING INTERVIEW GUIDE, HIGH-LEVEL QUESTIONS SHOULD BE ASKED FIRST WITH OPPORTUNITIES FOR PARTICIPANTS TO ANSWER AND REPLY BEFORE MOVING ON TO PROBES. NOT ALL PROBES NEED TO BE USED. ONLY USE PROBES AS REQUIRED: 1) TO STIMULATE RESPONSES WHEN YOU ARE NOT GETTING THEM; OR 2) TO ENSURE THE TOPIC HAS BEEN COVERED COMPREHENSIVELY BEFORE MOVING ON.]

Beliefs About and Attitudes Toward Health and Health Care

We are interested in broadly understanding how people in your community view health and healthcare services.

- How would you describe the overall health status of people in your community?
  - Probe: General levels of health?
  - Probe: Major issues? NCD vs. CD; chronic vs. acute
- How would you describe the overall status of health care in your community?
  - Probe: Views with respect to the quality of care across different care settings (public/private/NGO; clinic/hospital; other)
  - Probe: For NCD vs. CD; chronic vs. acute
- What are the key issues that support or hinder the quality of care across different care settings?
  - Probe: Specifically in providing care for NCDs?
- Where do people in your community typically get information about health and health care?
  - Probe: information about health?
  - Probe: information about health care and health care services?
  - Probe: Different for different groups of people? Explain.

Care-seeking Practices

We also would like to better understand when and how people in your community seek health care services.

- Under what circumstance do people seek care from health professionals?
  - Probe: Do people get regular check-ups or exams (preventive care) even when they are feeling OK? If no, why? If yes, where and why?
- Where do people in your community generally go when they are not feeling well?
  - Probe: Different places for different situations/needs?
- How often do people visit health providers, in general?
  - Probe: Generally healthy people?
  - Probe: People with some sort of chronic disease (e.g., asthma, CVD, diabetes)?
- How often do people seek care from someone other than a doctor (as a proportion of the above)?
  - Probe: Generally healthy people?
  - Probe: People with some sort of chronic disease (e.g., CVD, diabetes, asthma)?
  - Probe: From where/whom do they seek care, depending on the situation?
  - Probe: Why? (e.g., cost, trust, wait time, etc.)

Access to Healthcare

- What types of healthcare do you think people in your community can access easily?
  - Probe: by types of care - preventive, acute, chronic disease management, etc.
  - Probe: by types of setting – private/public/NGO, clinics, etc.
  - Probe: Why? (e.g., cost, information, etc.)
- What types of healthcare do you think it is hard for people in your community to access?
  - Probe: by types of care - preventive, acute, chronic disease management, etc.
  - Probe: by types of setting – private/public/NGO, clinics, etc.

    - Probe: Why? (e.g., cost, travel requirements, etc.)
- What groups of people might have difficulty accessing healthcare in your community?
    - Probe: by SES, social group, age?
    - Probe: Why? (e.g., cost, transportation, work, uninformed, etc.)
- What do you see as the primary barriers to access to healthcare in your community?
    - Probe: (e.g., cost, lack of providers, wait times, etc.)
    - Probe: Does this differ by types of care - preventive, acute, chronic disease management, etc.?
    - Probe: How does this differ by setting – private/public/NGO, clinics, etc.?
    - Probe: How does this differ by population groups?

Provision of Healthcare and Other Health-Related Services

<u>Broad community questions</u>

- Can you (each) provide a brief overview of your role in providing care to NCD patients – both here within the facility and any outside community activities/responsibilities?
- What do you find to be the most challenging aspect of working in healthcare in your community?
    - Probe: Specifically with respect to the identification and care (treatment/care management) of NCDs?
- How would you describe the relationship between healthcare workers and the community?
    - Probe: Health care professional trusted? Sought for advice and guidance?
- How would you describe the relationship between healthcare workers at different facilities?
    - Probe: Are there forma/informal cross-institution relationship for continuity across the continuum of care?

<u>Identification and treatment of NCDs within the community</u>

- How do NCD patients typically come to you for care?
    - Probe: Referrals? Self-initiated?
- How are NCDs typically identified/diagnosed in your community?
    - Probe: community outreach and education, leading to self-identification or CHW identification and a health care visit?
    - Probe: regular check ups with PCP?
- Do you believe your community is doing a good job in identifying people with NCDs and getting them in for treatment/care management?
    - Probe: What are the barriers to diagnosis, treatment, and care management?
    - Probe: What could be done to improve diagnosis, treatment, and care management?

<u>Identification and treatment of NCDs within your facility</u>

- Does your facility partake in any outreach activities within the community to help educate, inform, and identify NCDs? If yes, please describe.
    - Probe: If applicable, can we get copies of any program material?
- Are there specific protocols on treatment and follow up for patients who are diagnosed with such conditions as high blood pressure or diabetes? If so, what are they?
    - Probe: Were they developed by your facility, or do they come from elsewhere? Explain.

    - Probe: Do they include both medical regimens + lifestyle/behavioral change components?
    - Probe: How valuable do you find these protocols, and how widely are they utilized/followed by health care professionals both inside and outside (if applicable) your facility?
    - Probe: Can we get copies of any treatment or disease management protocols?
- Does your facility provide any counseling or coaching services to patients with NCDs in addition to medical treatment?
    - Probe: If not, is this available elsewhere?
- Can you describe general levels of adherence to treatment and care management programs among NCD patients at your facility?
    - What are the barriers to adherence?
    - What is in place that supports adherence (in your facility or community)?
- Have any refresher trainings on treatment for patients with NCDs been offered in your facility or in your area?
    - How many trainings? When?
    - Who organized the trainings?
    - If you attended, what was covered?
- What are the greatest challenges and opportunities for identifying, treating, and managing NCDs within your health care facility?
- If the budget at your health facility were doubled, what do you think would be the best way to spend the money?

General Assessment of Health Care Services and Ideas for Improvement

- Have any new programs or campaigns targeting prevention of NCDs been initiated in your community in the past five years?
    - What kind of programs?
    - Who runs them?
    - Do you have any documentation or materials you can share?
- Have any new programs or campaigns targeting patients living with NCDs been initiated in your community in the past five years?
    - What kind of programs?
    - Who runs them?
    - Do you have any documentation or materials you can share?
- What do you see as the greatest challenges in identifying and treating health care needs in your community?
    - Probe: How do you think these challenges could best be addressed?
    - Probe: What would be required to address them? (e.g., financial/human resources, institution responsible for filling particular gap, etc.)
- What do you see as good resources in your community for citizens to maintain good health?
- What do you see as resources lacking in your community for citizens to maintain good health?
- How could the health resources in your community be further developed and improved?

- What do you think the health care facilities, community leaders, or the government has done well in providing healthcare to the community?
  - Probe: Can you provide some examples? (e.g., specific programs or public health campaigns; community outreach and educational programs; policies for cross-institution relationship and continuity of care)
- What do you think the health care facilities, community leaders, or the government has done less well in providing healthcare to the community?
  - Probe: Can you provide some examples?
- Finally, what other kinds of things do you think could be done to improve the overall health of people in your community?
  - Probe: What ideas do you have?

Thank you all for your time and participation. The information and insights that you have provided today will be incredibly valuable to this project. If you have any questions, please feel free ask me or to contact me at the number/email listed on your study information sheet.

HEALTH SYSTEM OFFICIALS/POLICYMAKERS

FOCUS GROUP PROTOCOL PACKAGE

Objectives: To assess broad challenges and opportunities with access to and the provision of NCD care in the community; To gather specific information on local priorities, as well as current and future planned programs and policies.

Methodology: Interviews

Target participants:

- 1 Head health official at district health office
- 1 District hospital administrator
- 1 Official from an NGO/CBO providing health services (for NCDs if possible)
- Either:
    - 1 NCD-specific health official at district health office (first priority)
    - 1 Non-health-related government official at the district level

Recruitment strategy:

We will ask our local collaborators as well as the chief executive, medical, and operations officers at facilities recruited into the study to help us to identify appropriate health officials for interview, and possibly to assist us with introductions and/or contact information.

KEY INFORMANT INTERVIEW INFORMATION SHEET[2]

Type of interview:

☐ Physicians/Doctors **[PD]**

☐ Nurses and ASHAs **[NASH]**

☑ Health Officials **[HO]**

Catchment area:

District: ________________________________

City/Town: ________________________________

Interviewee job title: ____________________

Interviewee institution: ____________________

Date of Interview: ____

Name of facilitator: ____

Name of note taker/electronic recorder: ____

Interview Code: __I-KII-HO-XX______________________________________

**used to label and match all hardcopy and electronic files, transcript tapes, etc. and can be used in master file of qualitative data collection. Should include country, focus group vs. interview, and category of participants. For example, for interviews with Physicians or doctors in India, you use: I-KII-HO-1, I-KII-HO-2, etc.*

[2] **To be filled by focus group facilitator and attached to focus group transcriptions and/or notes**

KEY INFORMANT INTERVIEW GUIDE: HEALTH OFFICIALS

Introduction

Thank you for agreeing to participate in this key informant interview today. Before we get started, I would like review the overall purpose of our study and why we have asked you to come here today. I also will review how the information you provide to us today will be used, and obtain your agreement to participate, once this information is shared with you.

[READ INFORMED CONSENT WHICH SHOULD INCLUDE THE OVERALL PURPOSE OF THE STUDY AND HOW THE FOCUS GROUPS INTERVIEWS ARE AN IMPORTANT COMPONENT. OBTAIN INFORMED CONSENT AND LEAVE THE INFORMATION SHEET ABOUT THE PROJECT WITH PARTICIPANT, INCLUDING CONTACT INFORMATION FOR THE IN-COUNTRY STUDY LEAD FOR ANY FOLLOW-UP QUESTIONS.]

[IN THE FOLLOWING INTERVIEW GUIDE, HIGH-LEVEL QUESTIONS SHOULD BE ASKED FIRST WITH OPPORTUNITIES FOR PARTICIPANTS TO ANSWER AND REPLY BEFORE MOVING ON TO PROBES. NOT ALL PROBES NEED TO BE USED. ONLY USE PROBES AS REQUIRED: 1) TO STIMULATE RESPONSES WHEN YOU ARE NOT GETTING THEM; OR 2) TO ENSURE THE TOPIC HAS BEEN COVERED COMPREHENSIVELY BEFORE MOVING ON.]

Beliefs About and Attitudes Toward Health and Health Care

We are interested in broadly understanding the health status of the community and the various factors that contribute to the health of the community.

- How would you describe the overall health status of people in your community?
  - Probe: General levels of health?
  - Probe: Major issues? NCD vs. CD; chronic vs. acute
  - Probe: How has this changed (for better or worse) over time?
- How would you describe the overall status of health <u>care</u> in your community?
  - Probe: Views with respect to the quality of care across different care settings (public/private/NGO; clinic/hospital; other)
  - Probe: For NCD vs. CD; chronic vs. acute
  - Probe: How has this changed (for better or worse) over time?
- What are the top priorities that you have as a policymaker for improving the health and quality of health care in the community? Explain.

Access to Healthcare

- Can you broadly describe the access that people in your community have to healthcare?
  - Probe: What types of healthcare do you think people in your community can access easily?
  - Probe: What types of healthcare do you think it is hard for people in your community to access?
  - Probe: What groups of people might have difficulty accessing healthcare in your community?
- What do you see as the primary barriers to access to healthcare in your community?
  - Probe: (e.g., cost, lack of providers, wait times, etc.)
  - Probe: Does this differ by types of care - preventive, acute, chronic disease management, etc.?
  - Probe: How does this differ by setting – private/public/NGO, clinics, etc.?
  - Probe: How does this differ by population groups?
- What types of policies or programs have been introduced – or are being planned – to address any access issues in the community?
  - Probe: Who is planning/implementing these programs?
  - Probe: Who is funding these programs?
  - Probe: Do these programs target specific populations?

Provision of Healthcare and Other Health-Related Services

<u>Broad community questions</u>

- How would you describe the relationship between different health care facilities and other providers of health-related programs in the community?
  - Probe: Are there formal/informal cross-institution relationships for continuity across the continuum of care?
  - Probe: Are such relationships supported through policies and programs?
- How would you describe the relationship between health care facilities/programs in the community and health policymakers/decisionmakers [at the city/district/regional/state level]?

- Probe: Is there an open line of communication or a mechanism for facilities/programs to influence policies and other decisions that affect them? Do facilities/programs reach out to policymakers with ideas, and policymakers reach out to care givers for exchange of information and ideas? In what ways? Examples?

Identification and treatment of NCDs within the community

- Do you believe your community is doing a good job in identifying people with NCDs and getting them in for treatment/care management?
  - Probe: What are the barriers to diagnosis, treatment, and care management?
  - Probe: What could be done to improve diagnosis, treatment, and care management?
- Are there specific protocols on treatment and follow up for patients who are diagnosed with NCDs, such as high blood pressure or diabetes, at the community level?
  - Probe: If yes, by whom were they developed?
  - Probe: Do they include both medical regimens + lifestyle/behavioral change components?
  - Probe: How valuable do you find these protocols, and how widely are they followed by health care professionals in your community?
  - Probe: Can we get copies of any treatment or disease management protocols?
- What policies or programs have been put into place – or are being planned – to improve the diagnosis, treatment, and management of NCDs in your community?
  - Probe: Who is planning/implementing these programs?
  - Probe: Who is funding these programs?
  - Probe: Do these programs target specific populations?

General Assessment of Health Care Services and Ideas for Improvement

- What do you see as the greatest challenges in identifying and treating health care needs in your community?
  - Probe: How do you think these challenges could best be addressed?
  - Probe: What would be required to address them? (e.g., financial/human resources, institution responsible for filling particular gap, etc.)
- What do you see as good resources in your community for citizens to maintain good health?
- What do you see as resources lacking in your community for citizens to maintain good health?
- How could the health resources in your community be further developed and improved?
- What do you think the health care facilities, community leaders, or the government has done well in providing healthcare to the community?
  - Probe: Can you provide some examples? (e.g., specific programs or public health campaigns; community outreach and educational programs; policies for cross-institution relationship and continuity of care)
- What do you think the health care facilities, community leaders, or the government has done less well in providing healthcare to the community?
  - Probe: Can you provide some examples?
- What are your ideas and priorities for improving the overall health of people in your community?
  - Probe: What do you see as current major priority areas for health policy in your community?

- Probe: What do you see as key priority areas for health policy in your community in the future?
- Probe: If your budget was doubled, how would you choose to utilize the extra resources for improvements in the health of your community/constituency?

Document request

- Can you share with us any policy or program documentation related to NCD identification, treatment, and care management?

Thank you all for your time and participation. The information and insights that you have provided today will be incredibly valuable to this project. If you have any questions, please feel free ask me or to contact me at the number/email listed on your study information sheet.

FRONT-LINE HEALTH CARE PROVIDERS: NURSES/ASHAS

KEY INFORMANT INTERVIEW PROTOCOL PACKAGE

Objectives: To gather information on access to NCD care and experiences in the provision of that care from the perspective of NCD care givers.

Methodology: Interviews

Target participants: 2 Nurses and 3 ASHAs from facilities involved in the quantitative survey. Ideally drawn from a mix of the facility types: Private clinic, public district hospital, Primary Health Centre, Community Health Centre, Sub-Centre.

Recruitment strategy:

An initial communication about the overall study will come from facility management, explaining the purpose of the study and that both patients, providers, and administrators/management at the facility will be asked to participate. Follow-up communication to relevant staff will come from either facility management or the research team directly, depending on the arrangements made facility by facility. Volunteers will be asked schedule the interview on a specified date and time and will be informed of the purpose of the discussion as well as the nature of the questions (i.e., non-sensitive and non-personal) ahead of time—and that the session will be audio recorded for transcription and study. Oral informed consent will be obtained at the start of the interview.

KEY INFORMANT INTERVIEW INFORMATION SHEET[3]

Type of interview:

☐ Physicians/Doctors **[PD]**

☑ Nurses and ASHAs **[NASH]**

☐ Health Officials **[HO]**

Catchment area:

District: ________________________________

City/Town: ________________________________

Name of health care facility: __________________________

Health care facility information:

☐ Hospital	☐ Public
☐ Primary Health Centre	☐ Private
☐ Community Health Centre	
☐ Sub-Centre	

Interviewee job title: ___________________

Date of Interview: ____

Name of facilitator: ____

Name of note taker/electronic recorder: ____

Interview Code: __I-KII-NASH-XX______________________________________

**used to label and match all hardcopy and electronic files, transcript tapes, etc. and can be used in master file of qualitative data collection. Should include country, focus group vs. interview, and category of participants. For example, for interviews with Physicians or doctors in India, you use: I-KII-NASH-1, I-KII-NASH-2, etc.*

[3] **To be filled by focus group facilitator and attached to focus group transcriptions and/or notes**

KEY INFORMANT INTERVIEW GUIDE: NURSES AND ASHAS

Introduction

Thank you for agreeing to participate in this key informant interview today. Before we get started, I would like review the overall purpose of our study and why we have asked you to come here today. I also will review how the information you provide to us today will be used, and obtain your agreement to participate, once this information is shared with you.

[READ INFORMED CONSENT WHICH SHOULD INCLUDE THE OVERALL PURPOSE OF THE STUDY AND HOW THE FOCUS GROUPS INTERVIEWS ARE AN IMPORTANT COMPONENT. OBTAIN INFORMED CONSENT AND LEAVE THE INFORMATION SHEET ABOUT THE PROJECT WITH PARTICIPANT, INCLUDING CONTACT INFORMATION FOR THE IN-COUNTRY STUDY LEAD FOR ANY FOLLOW-UP QUESTIONS.]

[IN THE FOLLOWING INTERVIEW GUIDE, HIGH-LEVEL QUESTIONS SHOULD BE ASKED FIRST WITH OPPORTUNITIES FOR PARTICIPANTS TO ANSWER AND REPLY BEFORE MOVING ON TO PROBES. NOT ALL PROBES NEED TO BE USED. ONLY USE PROBES AS REQUIRED: 1) TO STIMULATE RESPONSES WHEN YOU ARE NOT GETTING THEM; OR 2) TO ENSURE THE TOPIC HAS BEEN COVERED COMPREHENSIVELY BEFORE MOVING ON.]

Beliefs About and Attitudes Toward Health and Health Care

We are interested in broadly understanding how people in your community view health and healthcare services.

- How would you describe the overall health status of people in your community?
  - Probe: General levels of health?
  - Probe: Major issues? NCD vs. CD; chronic vs. acute
- How would you describe the overall status of health care in your community?
  - Probe: Views with respect to the quality of care across different care settings (public/private/NGO; clinic/hospital; other)
  - Probe: For NCD vs. CD; chronic vs. acute
- What are the key issues that support or hinder the quality of care across different care settings?
  - Probe: Specifically in providing care for NCDs?
- Where do people in your community typically get information about health and health care?
  - Probe: information about health?
  - Probe: information about health care and health care services?
  - Probe: Different for different groups of people? Explain.

Care-seeking Practices

We also would like to better understand when and how people in your community seek health care services.

- Under what circumstance do people seek care from health professionals?
  - Probe: Do people get regular check-ups or exams (preventive care) even when they are feeling OK? If no, why? If yes, where and why?
- Where do people in your community generally go when they are not feeling well?
  - Probe: Different places for different situations/needs?
- How often do people visit health providers, in general?
  - Probe: Generally healthy people?
  - Probe: People with some sort of chronic disease (e.g., asthma, CVD, diabetes)?
- How often do people seek care from someone other than a doctor (as a proportion of the above)?
  - Probe: Generally healthy people?
  - Probe: People with some sort of chronic disease (e.g., CVD, diabetes, asthma)?
  - Probe: From where/whom do they seek care, depending on the situation?
  - Probe: Why? (e.g., cost, trust, wait time, etc.)

Access to Healthcare

- What types of healthcare do you think people in your community can access easily?
  - Probe: by types of care - preventive, acute, chronic disease management, etc.
  - Probe: by types of setting – private/public/NGO, clinics, etc.
  - Probe: Why? (e.g., cost, information, etc.)
- What types of healthcare do you think it is hard for people in your community to access?
  - Probe: by types of care - preventive, acute, chronic disease management, etc.

- Probe: by types of setting – private/public/NGO, clinics, etc.
  - Probe: Why? (e.g., cost, travel requirements, etc.)
- What groups of people might have difficulty accessing healthcare in your community?
  - Probe: by SES, social group, age?
  - Probe: Why? (e.g., cost, transportation, work, uninformed, etc.)
- What do you see as the primary barriers to access to healthcare in your community?
  - Probe: (e.g., cost, lack of providers, wait times, etc.)
  - Probe: Does this differ by types of care - preventive, acute, chronic disease management, etc.?
  - Probe: How does this differ by setting – private/public/NGO, clinics, etc.?
  - Probe: How does this differ by population groups?

Provision of Healthcare and Other Health-Related Services

<u>Broad community questions</u>

- Can you (each) provide a brief overview of your role in providing care to NCD patients – both here within the facility and any outside community activities/responsibilities?
- What do you find to be the most challenging aspect of working in healthcare in your community?
  - Probe: Specifically with respect to the identification and care (treatment/care management) of NCDs?
- How would you describe the relationship between healthcare workers and the community?
  - Probe: Health care professional trusted? Sought for advice and guidance?
- How would you describe the relationship between healthcare workers at different facilities?
  - Probe: Are there forma/informal cross-institution relationship for continuity across the continuum of care?

<u>Identification and treatment of NCDs within the community</u>

- How do NCD patients typically come to you for care?
  - Probe: Referrals? Self-initiated?
- How are NCDs typically identified/diagnosed in your community?
  - Probe: community outreach and education, leading to self-identification or CHW identification and a health care visit?
  - Probe: regular check ups with PCP?
- Do you believe your community is doing a good job in identifying people with NCDs and getting them in for treatment/care management?
  - Probe: What are the barriers to diagnosis, treatment, and care management?
  - Probe: What could be done to improve diagnosis, treatment, and care management?

<u>Identification and treatment of NCDs within your facility</u>

- Does your facility partake in any outreach activities within the community to help educate, inform, and identify NCDs? If yes, please describe.
  - Probe: If applicable, can we get copies of any program material?

- Are there specific protocols on treatment and follow up for patients who are diagnosed with such conditions as high blood pressure or diabetes? If so, what are they?
  - Probe: Were they developed by your facility, or do they come from elsewhere? Explain.
  - Probe: Do they include both medical regimens + lifestyle/behavioral change components?
  - Probe: How valuable do you find these protocols, and how widely are they utilized/followed by health care professionals both inside and outside (if applicable) your facility?
  - Probe: Can we get copies of any treatment or disease management protocols?
- Does your facility provide any counseling or coaching services to patients with NCDs in addition to medical treatment?
  - Probe: If not, is this available elsewhere?
- Can you describe general levels of adherence to treatment and care management programs among NCD patients at your facility?
  - What are the barriers to adherence?
  - What is in place that supports adherence (in your facility or community)?
- What are the greatest challenges and opportunities for identifying, treating, and managing NCDs within your health care facility?
- If the budget at your health facility were doubled, what do you think would be the best way to spend the money?

General Assessment of Health Care Services and Ideas for Improvement

- Have any refresher trainings on treatment for patients with NCDs or prevention of NCDs for healthy individuals been offered in your facility or in your area?
  - How many trainings? When?
  - Who organized the trainings?
  - If you attended, what was covered?
- Have any new programs or campaigns targeting prevention of NCDs been initiated in your community in the past five years?
  - What kind of programs?
  - Who runs them?
  - Do you have any documentation or materials you can share?
- Have any new programs or campaigns targeting patients living with NCDs been initiated in your community in the past five years?
  - What kind of programs?
  - Who runs them?
  - Do you have any documentation or materials you can share?
- What do you see as the greatest challenges in identifying and treating health care needs in your community?
  - Probe: How do you think these challenges could best be addressed?
  - Probe: What would be required to address them? (e.g., financial/human resources, institution responsible for filling particular gap, etc.)
- What do you see as good resources in your community for citizens to maintain good health?

- What do you see as resources lacking in your community for citizens to maintain good health?
- How could the health resources in your community be further developed and improved?
- What do you think the health care facilities, community leaders, or the government has done well in providing healthcare to the community?
    - Probe: Can you provide some examples? (e.g., specific programs or public health campaigns; community outreach and educational programs; policies for cross-institution relationship and continuity of care)
- What do you think the health care facilities, community leaders, or the government has done less well in providing healthcare to the community?
    - Probe: Can you provide some examples?
- Finally, what other kinds of things do you think could be done to improve the overall health of people in your community?
    - Probe: What ideas do you have?

Thank you all for your time and participation. The information and insights that you have provided today will be incredibly valuable to this project. If you have any questions, please feel free ask me or to contact me at the number/email listed on your study information sheet.
